# New InhA Inhibitors Based on Expanded Triclosan and *Di*-Triclosan Analogues to Develop a New Treatment for Tuberculosis

**DOI:** 10.3390/ph14040361

**Published:** 2021-04-14

**Authors:** Sarentha Chetty, Tom Armstrong, Shalu Sharma Kharkwal, William C. Drewe, Cristina I. De Matteis, Dimitrios Evangelopoulos, Sanjib Bhakta, Neil R. Thomas

**Affiliations:** 1 Biodiscovery Institute, School of Chemistry, University of Nottingham, University Park, Nottingham NG7 2RD, UK; Sarentha.Chetty@wits.ac.za (S.C.); tom.armstrong@nottingham.ac.uk (T.A.); shalu.sharma02@gmail.com (S.S.K.); Will.Drewe@lhasalimited.org (W.C.D.); 2 School of Pharmacy, University of Nottingham, University Park, Nottingham NG7 2RD, UK; Pazcm@exmail.nottingham.ac.uk; 3 Department of Biological Sciences, Birkbeck, Institute of Structural and Molecular Biology, University of London, London WC1E 7HX, UK; d.evangelopoulos@ucl.ac.uk (D.E.); s.bhakta@bbk.ac.uk (S.B.)

**Keywords:** tuberculosis, structure-based drug-design, molecular modelling, InhA, triazole, triclosan, isoniazid, *Mycobacterium tuberculosis*, *Mycobacterium bovis* BCG

## Abstract

The emergence of multidrug-resistant (MDR) and extensively drug-resistant (XDR) tuberculosis (TB) has reinforced the need for the development of new anti-TB drugs. The first line drug isoniazid inhibits InhA. This is a prodrug requiring activation by the enzyme KatG. Mutations in KatG have largely contributed to clinical isoniazid resistance. We aimed to design new ‘direct’ InhA inhibitors that obviate the need for activation by KatG, circumventing pre-existing resistance. In silico molecular modelling was used as part of a rational structure-based drug-design approach involving inspection of protein crystal structures of InhA:inhibitor complexes, including the broad spectrum antibiotic triclosan (TCS). One crystal structure exhibited the unusual presence of two triclosan molecules within the *Mycobacterium tuberculosis* InhA binding site. This became the basis of a strategy for the synthesis of novel inhibitors. A series of new, flexible ligands were designed and synthesised, expanding on the triclosan structure. Low Minimum Inhibitory Concentrations (MICs) were obtained for benzylphenyl compounds (**12**, **43** and **44**) and *di*-triclosan derivative (**39**), against *Mycobacterium bovis* BCG although these may also be inhibiting other enzymes. The ether linked *di*-triclosan derivative (**38**) displayed excellent in vitro isolated enzyme inhibition results comparable with triclosan, but at a higher MIC (125 µg mL^−1^). These compounds offer good opportunities as leads for further optimisation.

## 1. Introduction

Tuberculosis (TB) has been a major cause of morbidity and mortality for centuries. It is one of the top 10 causes of death worldwide and is the number one killer amongst the infectious diseases [1,2]. A deadly synergy exists between HIV and TB [2], and in recent years, HIV has significantly contributed to the increase in the number of people infected with and dying from TB [3].

Drug susceptible TB can be successfully treated using a three or four drug combination. Current directly observed treatment (short course) (DOTs) consists of a two-month course of first-line drugs: isoniazid (INH), rifampicin, pyrazinamide and/or ethambutol followed by a four-month course of isoniazid and rifampicin [4,5]. Over the past five decades, varying degrees of resistance to existing TB drugs have emerged. Multidrug-resistant tuberculosis (MDR-TB), characterized as resistant to rifampicin and isoniazid is now widespread and extensively drug resistant tuberculosis (XDR-TB) defined as MDR-TB together with resistance to second-line drugs such as quinolones and aminoglycosides is increasing [1,2]. It is estimated that one third of the world’s population is already infected with the latent form of the disease, which carries a lifelong risk of activation [1,2]. The high infection rates and growing resistance means the hunt for new drugs has become a global priority.

Mycolic acids are an essential component of the mycobacterial cell wall and are synthesized by the fatty acid biosynthetic pathway. Fatty acid biosynthesis in *Mycobacterium tuberculosis* (*Mtb*) occurs via two discrete pathways (FASI and FASII). In the ubiquitous FASI pathway, fatty acids of lengths C_16_ and C_24/26_ are biosynthesized from acetyl CoA and malonyl CoA. Elongation of these fatty acids into full length mycolic acids occurs in the FASII cycle. The steps in the FASI pathway are carried out by one large, multifunctional fatty acid synthase enzyme. This enzyme is also found in eukaryotes and advanced prokaryotes. The enzymes in the FASII pathway, however, are encoded by discrete genes which are not present in eukaryotes, thereby making the enzymes in this pathway clinically validated drug targets [6,7,8]. 

Isoniazid was first synthesized in 1952 and is one of the most effective drugs in the current TB arsenal [8,9]. It is a prodrug and its activation requires oxidation by the enzyme KatG, resulting in an unstable acyl radical intermediate or an acyl anion [10,11,12]. This, in turn, forms a covalent adduct with nicotinamide adenosine dinucleotide (NAD^+^). The INH-NAD adduct then binds to the active site of the enoyl reductase (ENR) InhA, its main target, and exerts its inhibitory action [13,14]. Prevention of fatty acyl substrate reduction stops full length mycolate production, resulting in cell lysis [15,16].

The majority of INH-resistance is attributed to point mutations in the KatG enzyme rather than InhA [8,12,16]. The S315 mutation of KatG accounts for 50–95% of INH-resistant clinical isolates [17,18]. This mutation does not prevent the natural catalase activity and mutant strains retain fitness and virulence [18]. INH resistance can also occur through mutations in the InhA active site (S94A, I47T, and I21V) preventing INH-NAD binding. Mutations in the InhA promoter region also result in overexpression of the protein operon [19]. While these InhA mutations do account for some resistance, KatG mutations are responsible for the vast majority of clinically observed INH-resistance [18]. 

The development of an inhibitor that acts directly on InhA without the requirement for prior activation would bypass KatG-mediated resistance. Triclosan (TCS) is a broad-spectrum antimicrobial that until recently has been found in several commercial products including toothpaste and mouthwashes. The withdrawal of TCS was due to its potential to disrupt endocrine function and in particular reproductive hormones [20]. These inherent problems could be present in the development of new TCS derivatives which will need to be evaluated for this activity. TCS has been shown to directly inhibit InhA at low concentrations, with higher concentrations resulting in disruption of bacterial protein synthesis [7,21,22]. The chlorine atoms on the TCS structure, however, make it highly lipophilic thereby lowering its oral bioavailability and thus limiting its potential as an anti-tuberculosis drug [22,23,24]. TCS also has a lower potency in comparison to INH thereby requiring significantly higher concentrations to elicit its effect [22]. 

### SAR and Design of Compounds

**Figure 1:**
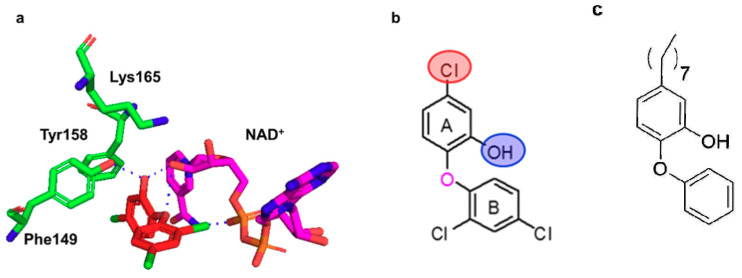
(**a**) Representation of TCS (red) bound to the active site of InhA. (Active site residues (green) and NAD^+^(magenta) (derived from PDB.2B35). (**b**) The constituents of the TCS structure important for activity as identified by Sivaraman et al. [26] are highlighted. (**c**) Structure of inhibitor 8PP.

The TCS:InhA crystal structure (PDB:1P45) reveals the key protein interactions [25]. The A-ring engages in л-stacking interactions with both the nicotinamide group on the cofactor and Phe149. The hydroxyl group forms a H-bonding network with the 2′-OH group on the ribose group of the NADH cofactor and Tyr158. An additional H-bond is made between the ether linkage and the ribose 2′-hydroxyl group [26]. These essential interactions are summarised in Figure 1b.

SAR studies by Sivaraman et al. revealed that for the *E. coli* enoyl reductase FabI, the two Cl substituents on the B-ring are not essential for activity as their removal causes a 7-fold decrease in the K_i_ value from 7 pM for TCS to 1 pM for 5-chloro-2-phenoxyphenol (CPP). This compound has also been characterized as a slow, tight-binding inhibitor with a significant delay in the onset of inhibition, probably due to a large conformational change in the FabI/NAD/TCS (or CPP) ternary complex. Both X-ray crystal and docked structures indicate that the chlorine at the ortho position on the B ring of TCS is in direct contact with the NAD^+^ phosphates, whilst simulations of the FabI/NAD^+^/CPP ternary complex indicate a significant change in the position of the NAD^+^ [26]. The A-ring Cl group is important for van der Waals interactions as well as decreasing the pK_a_ of the phenol in ring A from 9.12 to 7.8. Its removal therefore significantly affects the binding affinity to FabI [26,27]. The binding of CPP to InhA has not been reported, but Sullivan et al. demonstrated that potent inhibition of InhA could be achieved through replacement of the A-ring Cl atom with aliphatic chains of varying lengths, whilst only having an unsubstituted phenyl as the B-ring. The best analogue from the diphenyl ether series of compounds featured an octyl chain (8PP) (Figure 1) [28]. The chain was shown to engage the substrate binding loop, matching the arrangement seen by Rozwarski et al. with their C16 substrate mimic [28,29]. In the quest for new inhibitors, other research groups have since explored different modifications of the core TCS structure [23,25,26,28,30,31,32,33,34,35,36,37,38,39,40,41,42,43,44,45,46,47]. Rodriguez et al. explored combining two TCS molecules and synthesised a novel macrocyclic InhA inhibitor [48]. A recent review by Vosátka et al. provides a comprehensive summary of efforts to date [22]. More recently groups have employed computational techniques including virtual screening, density functional theory (DFT) calculations and QSAR to identify potential InhA inhibitors [23,49].

**Figure 2:**
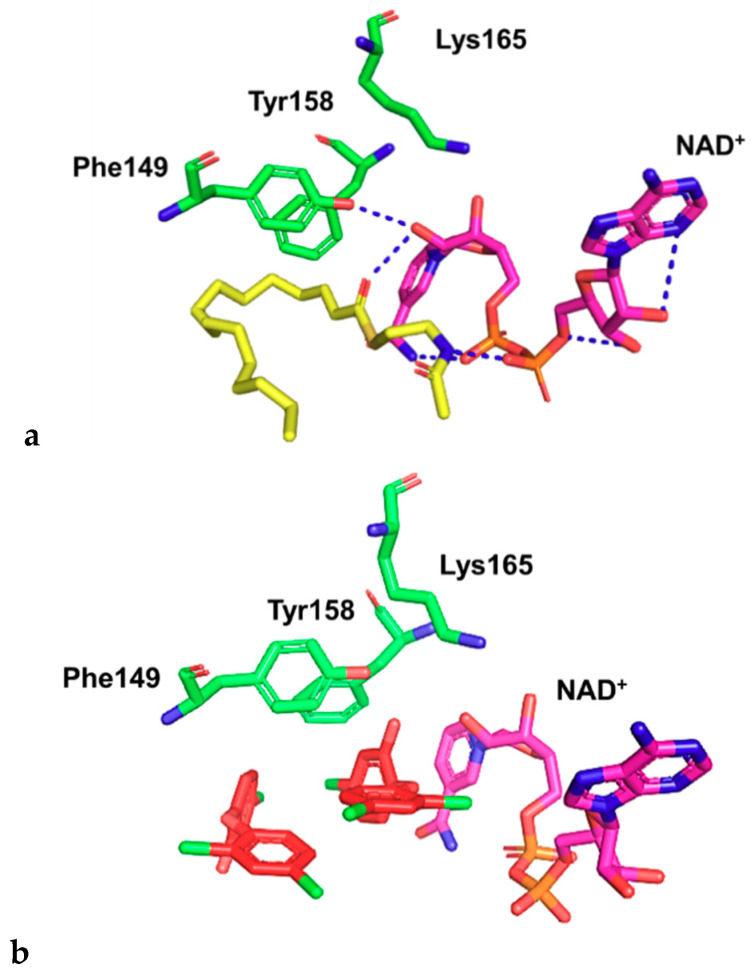
(**a**) A representation of the interaction of a C16 fatty acyl substrate (yellow) with the InhA active site residues (green) and the cofactor NAD^+^ (magenta), H-bonds (blue) (from PDB.1BVR) [29]. (**b**) A view of the key interactions of two molecules of TCS within the active site. TCS molecules (red), the active site residues (green) and NAD^+^ (magenta) (PDB.1P45). The two TCS molecules are separated by a distance of 4.6 Å between the two carbons attached to the B-ring *para*-chlorine atoms.

Interestingly, the X-ray crystallography work (PDB:1P45) conducted by Kuo et al. [25] revealed the presence of two TCS molecules within the InhA active site. This was unique to *Mycobacterial* InhA and not found in the crystal structures of enoyl reductases from any other organisms solved to date. This occurrence was presumably due to InhA having a longer substrate binding loop to accommodate a larger substrate. One molecule occupies the same space and is in the same orientation as that of all the other TCS-ENR complexes. The second TCS molecule lies adjacent to the first, with its A- and B-rings aligned with those of the first TCS, engaging the substrate binding loop and promoting loop ordering (Figure 2) [25].

**Figure 3:**
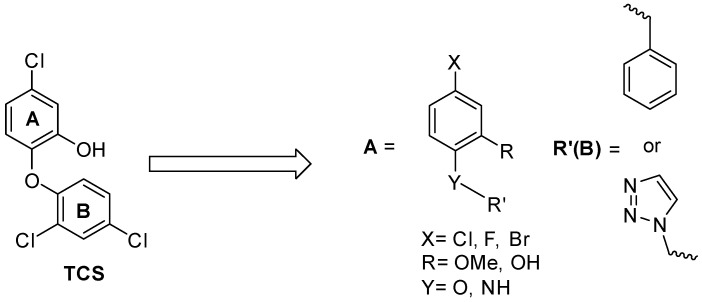
Overall target summary.

We reasoned that a *di*-triclosan molecule would be able to preferentially fit in the InhA active site and have reduced affinity for the enoyl reductases of other organisms, thereby having the potential to be selective for InhA. This was the basis of our drug design approach which aimed to bridge the distance of 4.6 Å between the 4′-carbon atoms of the two TCS molecules by a symmetrical and flexible ether linkage. We hypothesised that either a longer more flexible chain or a well-chosen rigid linker (see also reference [47] for a series of rotational restricted compounds based on the 1,5-triazole group) may allow the enlarged molecule to adopt a conformation similar to that of the fatty acylCoA substrate or the two TCS molecules within the loop. By retaining the most essential features of TCS that give it antibacterial activity, we generated three scaffolds (Figure 3). 

The Cl atom on the A-ring was either retained or substituted for by another halogen (F, Br). The hydroxyl substituent on the A-ring was replaced with a methoxy group. It was envisaged that the hydroxylated derivatives would also be tested but attempts to selectively demethylate this were unsuccessful. We retained the inclusion of the methoxy compounds as this functional group could still act as a H-bond acceptor with the co-factor and the active site residues. The B-ring was either retained as an aryl group or replaced by a heterocyclic ring (Figure 3). For the benzyl phenyl ether and aniline derivatives, the B-ring was kept similar to TCS, but both the chloride atoms were removed, and the *para*-Cl substituent was replaced with a methoxy substituent. The ether linkage was maintained or replaced with an amine linkage and further extended by one carbon unit. The reason for choosing these linkers was their ease of synthesis by a nucleophilic substitution reaction and to provide rotational flexibility which would allow the molecules to explore more of the binding site occupied by the second TCS and form stronger interactions with the flexible binding loop. The hypothesis was that the amine linkage should have the same stabilizing activity to that of the ether linkage in the TCS molecule in the TCS: InhA complex. The replacement of the ether group for an amine group could also affect the H-bonding as a secondary amine can function as both a H-bond donor and acceptor. For the triazole linked derivatives, the B-ring was replaced with a 1,4-triazole ring. The basis of the different scaffold was to provide opportunity to easily extend the scaffold further by one or two rings. It was hoped that a larger molecule would mimic the second TCS and form more interactions within the loop, and cause loop ordering. The triazole is also a bio-isostere of benzene and could adopt a similar orientation to that of the B-ring in TCS. It was anticipated that one of the triazole nitrogens could also form an additional H-bond with the cofactor. It was envisaged that the additional aromatic ring would increase the interactions with the cofactor and other hydrophobic residues in this region. 

We chose to incorporate different halogens as their different properties with regards to electronegativity and size determine their impact on the structure-activity relationship (SAR), binding to the receptor and cellular uptake. Sivaraman et al. demonstrated that even subtle changes in the steric and electronic properties of the ring can have a major effect on the binding potential [26,27]. 

A series of benzylphenyl aniline, triazole linked biaryl anilines and *di*-triclosan analogues were developed to find suitable new lead compounds and optimise them further following biological and SAR analyses. The long-term goal is to obtain inhibitors with good selectivity for InhA whilst being mindful of the requirements for compounds to be orally active and be prepared at low cost given the funding constraints of the healthcare systems of many of those affected by TB.

## 2. Results and Discussion

### 2.1. Drug-Like Properties

Assessments of the compounds from the three series of compounds for drug-like properties (Table 1) revealed all comply with Lipinski’s rule of five except compound **31** which is non-compliant with regards to both MW and ClogP [50] and **38** with regards to ClogP. These compounds were found to be soluble at 100 and 50 μM concentrations respectively, as used in the screening procedure. Calculations of topological polar surface area (TPSA) using a web-based molecular descriptors calculator (http://www.molinspiration.com, accessed on 25 March 2019) revealed that all compounds designed had a TPSA < 140 Å which is indicative of a reasonable potential to penetrate membranes. 

### 2.2. Docking Studies

Docking of the designed analogues into the active site gave a prediction of the binding mode. Overall, all molecules fitted well into the active site with the *di*-triclosan analogues having the best fitness scores followed by the triazole linked analogues, and then the benzyl phenyl ether or aniline analogues. This was not surprising as the *di*-triclosan molecules, having larger surface areas, made more favourable van der Waals and hydrophobic interactions with the residues and cofactor within the active site and hence gave higher scores. A summary of the docking scores for the individual compound series together with interactions with active site residues is provided in the Supplementary Information (Tables S1–S4; Figures S1–S4). The docking results suggest that the halogen in the *para* position on ring A, could have a significant effect on binding. Fitness scores were very similar for the benzyl phenyl and triazole ether-linked diaryl series with the trend being Br > Cl > F. For the benzylphenyl aniline and triazole-linked diaryl aniline analogues it was observed that Cl > F.

### 2.3. Synthesis

The three scaffolds for the target molecules were synthesized according to the general routes as follows: The benzyl phenyl analogues were prepared via a nucleophilic substitution reaction, the triazole-linked diaryl derivatives via a copper (I) catalysed alkyne-azide cycloaddition (CuAAC) ‘click’ reaction and the *di*-triclosan target molecules via a Buchwald coupling reaction to generate the methylated analogues. 

**Scheme 1:**
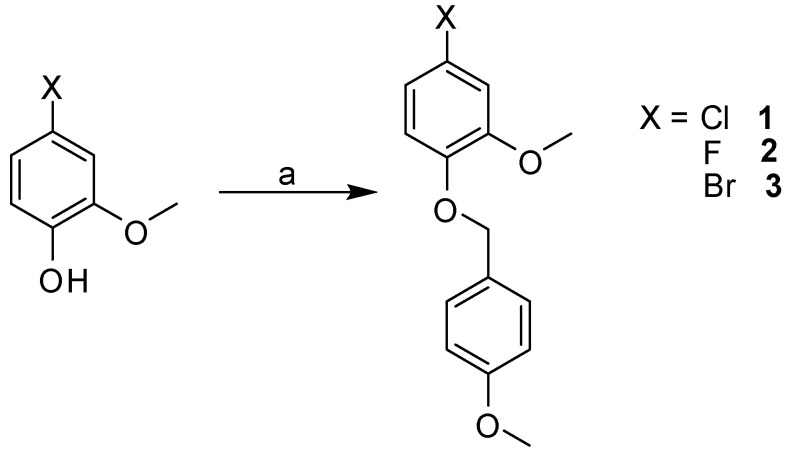
Synthesis of benzylphenylether derivatives. *Reagents and conditions*: (**a**) PMBCl, NaH, DMF, 0 °C to r.t., 4–6 h, X = Cl (29%), F (47%), Br (23%).

The synthesis of the benzylphenyl ether compounds **1**, **2**, **3** with the ether linkage is described in Scheme 1. Moderate yields from 23–47% were achieved.

**Scheme 2:**
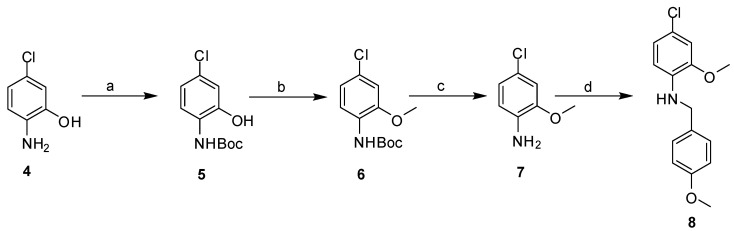
Synthesis of the chlorinated *N*-benzylphenyl aniline derivative **8**. *Reagents and conditions*: (**a**) Boc_2_O, NaHCO_3_, H_2_O, 0 °C to r.t., 56%; (**b**) MeI, K_2_CO_3_, acetone, 60 °C, 6.5 h, 82%; (**c**) TFA, DCM, 0 °C to r.t., 2 h, 85%; (**d**) PMBCl, NEt_3_, DCM, 0 °C to r.t., 5.5 h, 12%.

**Scheme 3:**
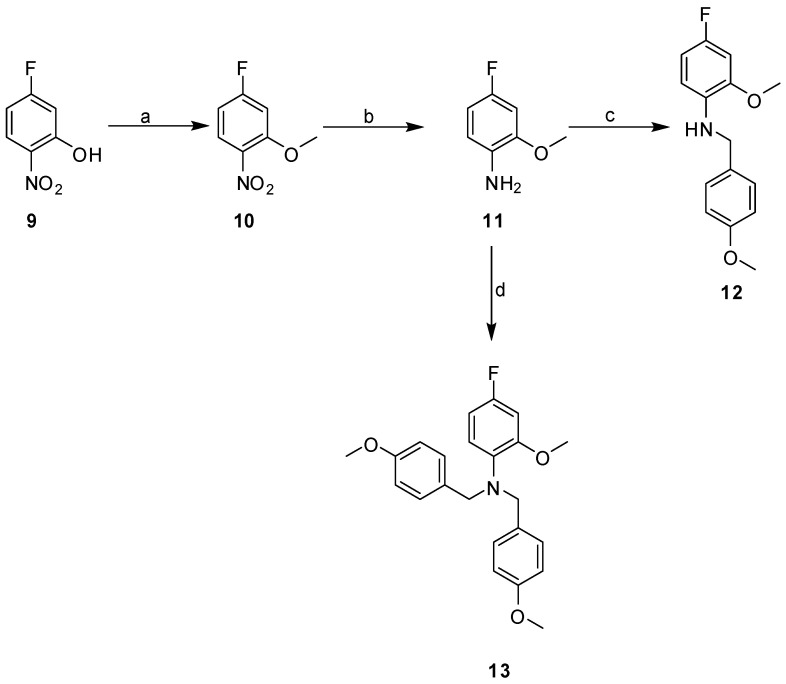
Synthesis of the fluorinated *N*-benzylphenylaniline derivatives **12** and **13**. *Reagents and conditions*: (**a**) MeI, K_2_CO_3_, 67–69 °C, 6 h; 81%; (**b**) NH_4_HCO_2_, 10% Pd/C, MeOH, r.t.; overnight, 100%; (**c**) PMBCl, K_2_CO_3_, DMF, 55 °C, 4 h, 14%; (**d**) PMBCl (3 equiv.), K_2_CO_3_, DMF, 55 °C, 4 h, 36%.

The benzylated aniline compounds were synthesized as detailed in Scheme 2 and Scheme 3. The commercially available starting materials for the chlorinated and fluorinated derivatives were different; requiring slightly different synthetic routes. The 2-methoxy-4-chloroaniline was generated from an amine starting material **4**. The free amine was initially protected with a Boc group. Methylation of the free hydroxyl group followed by removal of the Boc group generated the desired 2-methoxy-4-chloroaniline (**7**, Scheme 2). Nucleophilic substitution with *p*-methoxybenzyl chloride (PMBCl) gave the final compound **8** in 12% yield.

The fluorinated derivative **12**, was prepared from 4-fluoro-6-nitrophenol (**9**) as described in Scheme 3. The phenol group was first converted to its methyl ether followed by a reduction of the nitro group to generate amine **11**. Amine **11** was then subjected to nucleophilic substitution to generate target molecule **12** in 14% yield. In addition, a nucleophilic substitution reaction with 3 equivalents of PMBCl yielded an *N*, *N*-substituted amine **13** in 36% yield. The synthesis of this compound has been reported previously [51].

**Scheme 4:**
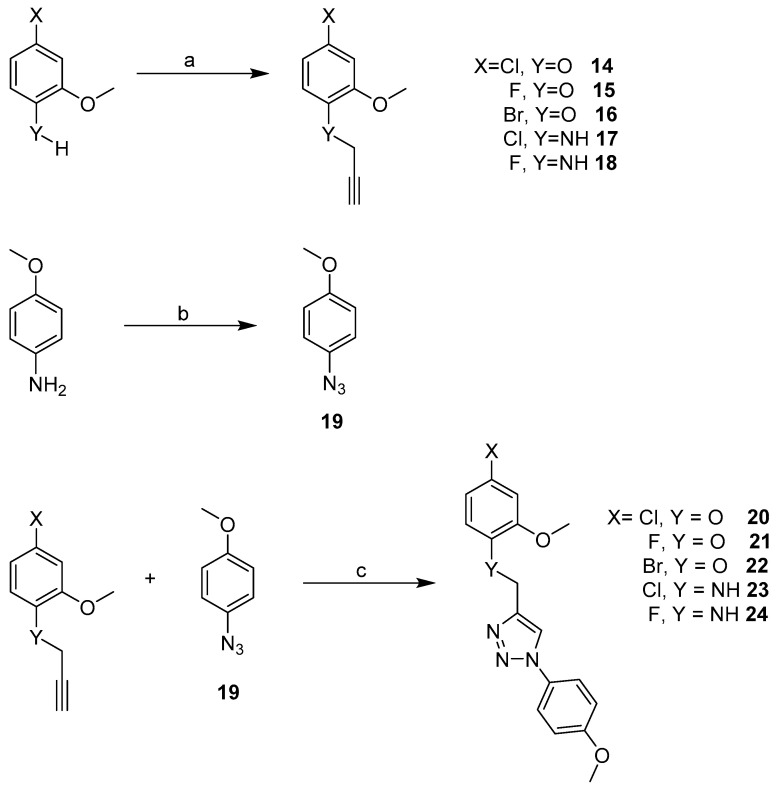
Synthesis of the triazole-linked diaryl compounds via ‘click’ chemistry. *Reagents and conditions*: (**a**) Propargyl bromide, K_2_CO_3_, DMF, 55 °C to r.t., 4 h, Y = O, X = Cl (75%), F (75%), Br (71%), Y = NH, X = Cl (7%), F (30%) ; (**b**) H_2_SO_4_, NaNO_2_, H_2_O, 0°C to r.t., 0.5 h followed by NaN_3_, H_2_O, r.t., 3 h, 82%; (**c**) ^t^BuOH, sodium ascorbate, CuSO_4_, H_2_O, r.t., overnight, Y = O, X = Cl (74%), F (77%), Br (83%), Y = NH, X = Cl (84%), F (77%).

The next set of target compounds (triazole-linked diaryl target molecules) was synthesised using a CuAAC ‘click’ chemistry strategy, as shown in Scheme 4. The triazole-linked target molecules were synthesised by subjecting the respective aniline or phenol to a nucleophilic substitution with propargyl bromide to generate the terminal alkynes: **14**–**18**. The azide **19** was formed by diazotisation of the commercially available aniline, followed by the CuAAC to generate the target molecules **20** (74%), **21** (77%), **22** (83%), **23** (84%), **24** (77%), respectively, in excellent yields.

**Scheme 5:**
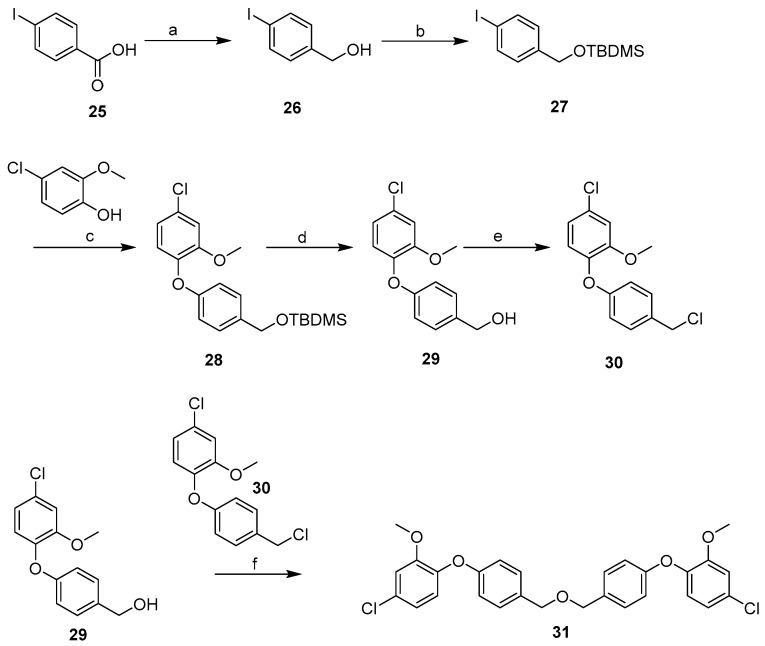
Synthesis of the di-triclosan dimethyl ether **31**. *Reagents and conditions*: (**a**) BH_3_, THF, r.t., overnight, 91%; (**b**) TBDMSCl, imidazole, DCM, r.t, overnight, 94% (**c**) CuI, Cs_2_CO_3_, naphthoic acid, toluene, r.t., 3 days, 41%; (**d**) TBAF, THF, 0 °C to r.t., 4.5 h, 79%; (**e**) MsCl, Et_3_N, 0 °C to r.t., 4 h, 65%; (**f**) NaH, THF, r.t. to 78 °C, 18 h, 79%.

**Scheme 6:**
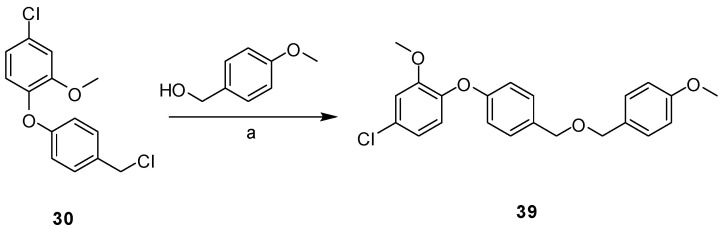
Synthesis of *di*-triclosan derivative **39.** *Reagents and conditions*: (**a**) NaH, DMF, r.t. to 0 °C to r.t., 4.5 h, 67%.

For the *di-*triclosan derivatives, the synthesis (Scheme 5) started with an initial Buchwald coupling followed by a nucleophilic substitution reaction to join symmetrical halves to generate *di*-triclosan analogue **31** in a 79% yield. The same strategy as outlined in Scheme 5 was applied to obtain unsymmetrical compound **39** in a 67% yield (Scheme 6). 

**Scheme 7:**
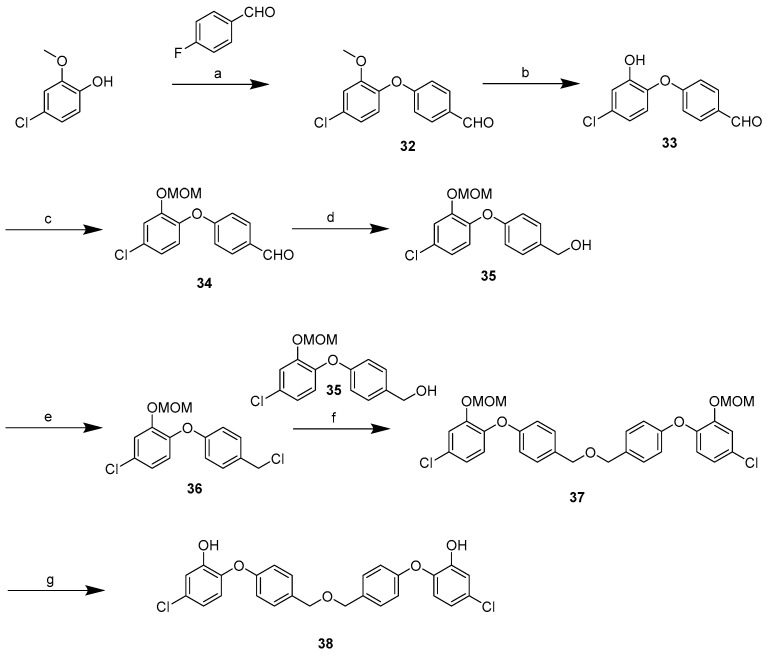
Synthesis of *di*-triclosan derivative **38**. *Reagents and conditions*: (**a**) K_2_CO_3_, DMF, 130 °C, 18 h, 89%, (**b**) AcOH, 47% HBr_aq_, 110 °C, 18 h, 29%, (**c**) DIPEA, MOMCl, r.t., 18 h, 92%, (**d**) NaBH_4_, MeOH, 0 °C to r.t., 4 h, 86%, (**e**) Et_3_N, MsCl, 0 °C to rt, 4 h, 56%, (**f**) NaH, DMF, 0 °C, 1 h, 45%, (**g**) 6 M HCl, MeOH, 70 °C 2 h, 44%**.**

Attempts to demethylate derivative **31** were unsuccessful, therefore another approach was adopted. The hydroxylated symmetrical *di*-triclosan derivative **38** followed a similar synthetic route (Scheme 7), but had different starting materials, demethylation and reprotection of the resulting alcohol with a MOM protecting group followed by reduction of the aldehyde to generate the alcohol **35**. A nucleophilic reaction followed by deprotection generated the final hydroxylated *di*-triclosan derivative **38** in a 44% yield. The three *di*-triclosan derivatives were thus successfully synthesized in moderate yields. 

**Scheme 8:**
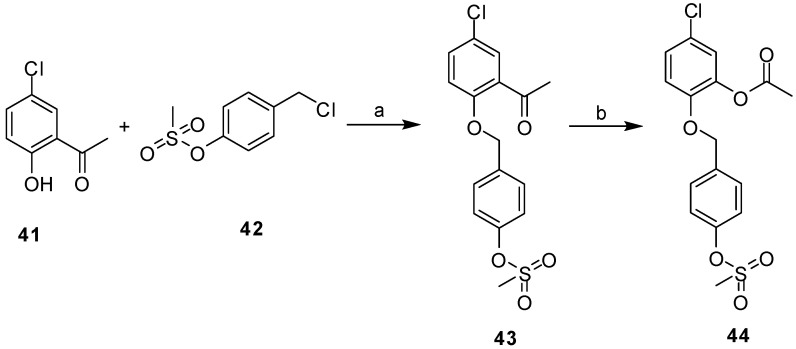
Synthesis of the benzylphenyl compounds via a Baeyer-Villiger oxidation. *Reagents and conditions*: (**a**) K_2_CO_3_, DMF, 55 °C to r.t., 4 h, 51%; (**b**) *m*CPBA, CHCl_3_, r.t., overnight, 68%.

The dibenzylether **40** was obtained in two steps from iodobenzoic acid by reduction with BH_3_ followed by an S_N_1 reaction with 4-iodobenzyl bromide to give **40** in 71% yield. This intermediate was produced *en route* to finding a suitable method to generate the *di*-triclosan derivatives. We thought it might be useful to test against *Mtb*. This compound has been previously synthesized for other purposes [52]. Ketone **41** was subjected to a nucleophilic substitution reaction followed by a Baeyer-Villiger oxidation which converted the ketone **43** to an ester **44** (Scheme 8).

### 2.4. Inhibition Studies with Purified InhA

**Figure 4:**
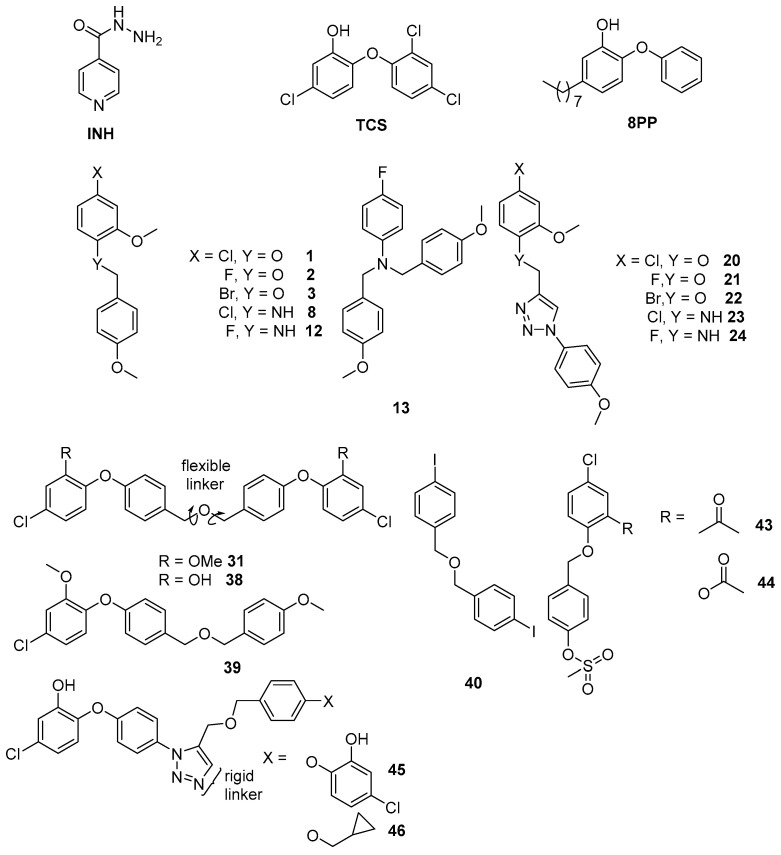
Compounds that were evaluated for biological activity together with compounds **45** and **46** from our previous study on a related series of triazole-linked *di*-triclosan analogues (47) and compound 8PP that is one of the most potent triclosan derivatives reported to date (IC_50_ 5.0 ± 0.3 nM; (*M. tuberculosis* H37_RV_)) [28].

Initial screening was carried out on all the TCS analogues and some of the intermediates at 100 μM, with the exception of compounds **1**, **3**, **20** and **38** (50 μM) and **22** (25 μM) due to their limited solubility (Table 1 with structures in Figure 4). The percentage inhibition results revealed that those compounds with good inhibition (>50% against InhA) were compounds **8**, **21**, and **38,** moderate inhibition (20–50%) was observed with **3**, **13**, **20**, **23**, **24**, **32**, **39**, **40**, **43** and poor inhibition (<20%) with **2** and **44**. Compounds **1**, **22** and **12** were inactive. 

### 2.5. Minimum Inhibitory Concentration (MIC) 

The triclosan analogues showed moderate to good inhibition of mycobacterial growth. The MIC data suggested that **12**, **39**, **43** and **44** has good inhibition properties, but in vitro assays revealed that **39** and **43** had only moderate inhibition of InhA, with compound **12** not inhibiting the enzyme. Likewise, **44** had reasonable inhibition properties against whole cells, but the in vitro assays revealed that this compound had poor inhibition properties against InhA. There is a possibility that these compounds inhibit other enzymes in the micro-organism given their MIC values. At high concentrations TCS has been reported to act as a mitochondrial uncoupling agent, cell membrane disruptor and to cause perturbations to lipid and protein biosynthesis.

### 2.6. Correlation of the Docking and Isolated Enzyme Results 

**Figure 5:**
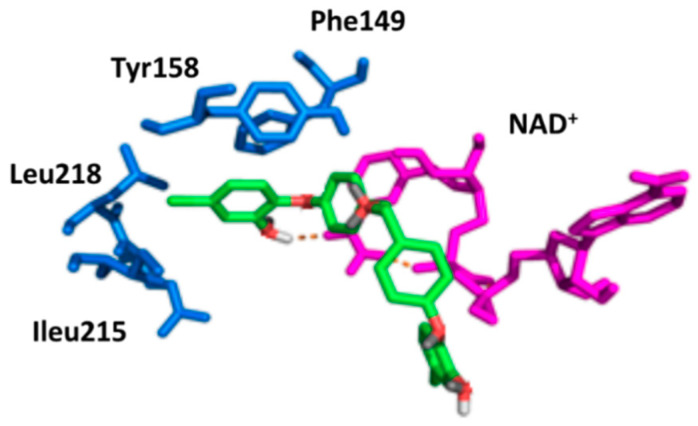
A view of the docking of **38** into the InhA active site. The active site residues (blue), NAD^+^ (magenta) and ligand (green).

The docking data for the hydroxylated di-triclosan derivative **38** correlated well with the inhibition data. Compound **38** displayed 100% inhibition at just 50 µM and the docked structure predicts a hydrogen bond as well as a л-л stacking with the cofactor. An additional л-л stacking interaction was predicted to occur with Phe 149 as well as favourable van der Waals interactions with residues Leu 218 and Ile 215 (Figure 5).

### 2.7. Structure Activity Relationships for the Compounds Evaluated 

The mycobacterial cell envelope is known to be a significant barrier to drugs therefore it is interesting to observe that compounds given in Table 1 with a ClogP range of −0.67 to 7.88, all have some activity in the whole cell assay. The largest compound tested here is still considerably smaller than rifampicin (MW 822.95; 6 HBD; 16 HBA; CLogP 3.71; TPSA 220.15) which is used in the clinical treatment of tuberculosis and is active against the latent form of the disease.

The compounds that have methyl ethers on the A- and B-rings (**1**–**3**, **8**, **12**, **20**–**24**, **31**) all show poor activity against whole *M. bovis*, even when active against InhA directly. Their level of activity against the isolated InhA is lower than those triclosan analogues that have a free hydroxyl group on the ‘A’-ring such as **38** and 8PP. This may be due to the methoxy group on the ‘A-ring’ being unable to behave as an efficient hydrogen bond acceptor with Tyr158 in the same way that the hydroxyl group on one of the triclosan molecules. Compound **39** has the methoxy ether and is only a moderate inhibitor of InhA but has one of the lowest MIC values of the compounds tested suggesting it may have other targets in mycobacteria. Extending this compound to give the full ether linked *di*-triclosan structure **38** whilst improving the activity against the isolated enzyme when compared with its dimethyl ether **31** is detrimental to its activity against whole cells. The compounds in which the B-ring of triclosan is replaced by a triazole group, whilst exhibiting moderate activity against the isolated enzyme, have poor activity against the whole cells suggesting that the introduction of this group is impacting on their uptake. The removal of the methyl ethers from compound **31** significantly improves its activity against both InhA and *M. bovis*.

A number of points that can be gleaned from examining the structure activity relationships for closely related compounds. Whilst compound **12** exhibits a low MIC value (7.8 µg mL^−1^), it does not inhibit the isolated InhA. Replacing the chlorine with a fluorine gives compound **8**. This change increases the MIC to >50 mM, but does generate a compound that inhibits InhA (63% at 40 µM). Given the smaller size of the fluorine, the change in activity may be due to the increase in basicity of the amine in this compound compared with the chlorine rather than any steric effects as compound **12** is predicted to bind tighter to InhA than **8**.

Compounds **43** and **44** differ in the nature of the substituent *ortho* to the phenolic ether, with the mesomerically electron donating ester substituent which would increase the HBA ability of the phenolic ether giving slightly better binding to InhA, but a higher MIC, whilst these properties are reversed with the electron withdrawing keto-substituent, suggesting that groups that retaining an electron withdrawing group in this position is preferred.

## 3. Materials and Methods

### 3.1. Molecular Docking and Design studies

The 2H7I.PDB [33] crystal structure (resolution 1.62 Å), containing InhA in complex with its respective ligand (1-cyclohexyl-5-oxo-*N*-phenylpyrrolidine-3-carboxamide) was retrieved from the Protein Data Bank (PDB). Prior to docking, the protein structure was prepared using the SYBYL X [53] structure preparation tool. The Amber force field FF99 was used to assign atom types. Gasteiger-Hückel charges were assigned to the ligand and cofactor. The cofactor was kept rigid during protein minimization. Protein minimization was done in three consecutive steps. The first step was hydrogen atom minimization keeping the NAD^+^ and heavy atoms rigid, followed by side-chain minimization keeping the NAD^+^ and backbone rigid. The last step was the whole protein minimization keeping the NAD^+^ rigid. Minimization was carried out with a maximum of 1000 iterations, gradient of 0.05 kcalmol^−1^, no initial optimization and using the Tripos force field. 

Molecular docking studies were performed using Genetic Optimization for Ligand Docking (GOLD) version 3.1. (CCDC, Cambridge, UK) [54,55]. Crystallographic waters were removed from the minimized complex structure and the ligand was extracted from the active site. The protein (with cofactor) was loaded with the corresponding ligand as a reference point into GOLD. The active site was defined by a 10 Å radius around the reference ligand.

The designed molecules were sketched in SYBYL X [53], assigned Gasteiger Hückel charges and minimized with a maximum of 5000 iterations, no initial optimization and using the Tripos forcefield. Docking was carried out using the default speed in GOLD. The binding site was defined by x (43.4601), y (51.7939), z (−82.8350) coordinates established in SYBYL using the reference ligand from pdb.2H7I. Fitness scores were determined using GOLDSCORE. For simplicity, the top scoring pose of each designed molecule was assessed.

ACD Labs [50] version 12 was used to calculate the ClogP of all the designed compounds and TPSA was calculated using the Molinspiration Cheminformatics server [56].

### 3.2. Synthesis

#### 3.2.1. Materials and General Methods

All reagents were supplied by either Sigma-Aldrich (part of Merck Life Science UK Limited, Gillingham, Dorset, UK), Alfa Aesar (Avocado, Lancashire, UK), or Fisher Scientific UK Ltd. (Loughborough, UK). Flash chromatography was performed on silica gel (35–70 µm, 60 Å (Fluorochem, Glossop, UK)). Elution was achieved under forced flow with the indicated reagent grade solvent mixtures. These are given in *v*/*v* ratios. All fractions containing the desired product were pooled together, with solvent removal under reduced pressure and dried under high vacuum to give the product. Melting points were obtained using a SMP_3_ melting point apparatus (Stuart Scientific, Stone, UK) and are uncorrected. Infrared spectra were recorded on the Tensor 27 FT-IR instrument (Bruker, Coventry, UK) using diluted solutions in spectroscopic grade chloroform or within a KBr disc or in solid state using a IR 200 FT-IR instrument (Nicolet, ThermoFisher, Loughborough, UK) ^1^H-NMR spectra and ^13^C-NMR spectra were recorded on an AV400 spectrometer (Bruker, Coventry, UK) at 400 MHz and 100 MHz, respectively, and were carried out at ambient temperature and measured using CDCl_3_ (δ_H_ = 7.26), D_2_O (δ_H_ = 4.87), d_4_-MeOH (δ_H_ = 3.31) or d_6_-DMSO (δ_H_ = 2.50) as solvent internal reference. ^1^H-NMR spectra are reported as follows: chemical shifts in ppm on a δ scale; multiplicity (app. = apparent, b = broad, s = singlet, d = doublet, t = triplet, q = quartet, m = multiplet); coupling constant *J* reported in Hz and quoted to the nearest 0.1 Hz. ^13^C-NMR spectra were recorded on a Bruker AV400 spectrometer at 100 MHz, at ambient temperature and were measured using d_3_-CDCl_3_ (δ_c_ = 77.0), d_4_-MeOH (δ_H_ = 49.0), or d_6_-DMSO (δ_c_ = 39.5) as a solvent internal reference and proton decoupled. ^13^C NMR spectra are reported in ppm on the δ scale. ^19^F NMR spectra were recorded on the Bruker AV400 spectrometer at 376.5 MHz. High performance liquid chromatography (HPLC) was performed using Agilent 1200 and Agilent 1100 series systems (Agilent, Santa Clara, CA, USA). The columns used were the Agilent Eclipse XDB-C18 column (150 × 4.6 mm, 5 µm) or an Agilent Eclipse XDB-C18 column (150 × 9.4 mm, 5 µm). Mass spectra were recorded on a Micromass LCT spectrometer (Micromass Ltd, Manchester, UK) using electrospray ionization (ESI) at 3000 eV, Micromass Autospec (EI), or a Bruker MicroTOF (ESI). Elemental analysis was recorded on a CE-440 Elemental Analyzer (Exeter Analytical, Coventry, UK). Analytical information on individual synthesized compounds is given in the Supplementary Information.

#### 3.2.2. General Procedure for Library Synthesis

The General Procedures (A-E) listed below were used as indicated in the Supplementary Information Experimental Section to prepare the compounds evaluated in this paper.

- General Procedure for Synthesis of the Benzylphenyl ether Compounds (A)

The general method was carried from an adapted literature procedure [57]. To a suspension of NaH (60% dispersion in mineral oil) (1.5 eq) in DMF (1.6 mL/1.0 mmol) cooled to 0 °C and flushed with nitrogen, a solution of the alcohol (1 eq) in DMF was added slowly via a syringe. The suspension was stirred at room temperature for 20 min and then cooled to 0 °C, followed by the dropwise addition of *p*-methoxybenzyl chloride (PMBCl) (3 eq) via a syringe. The pale-yellow solution was stirred for 4–6 h at room temperature, followed by neutralisation with saturated NH_4_Cl_(aq)_ solution. The solvent was removed by evaporation and the residue was dissolved in DCM. The organic layer was washed with water (2 × 50 mL) and brine (2 × 50 mL) and dried over MgSO_4_, before the solvent was removed *in vacuo* to give the crude product.

- General Procedure for the Synthesis of the Propargyl Analogues (B)

The general method was carried out from an adapted literature procedure [58]. Under an inert atmosphere of nitrogen, the required aniline/alcohol (1 eq) was dissolved in anhydrous DMF (0.5 mmol/1 mL), followed by the addition of K_2_CO_3_ (2 eq), before heating to between 53–55 °C for 30 min. The reaction mixture was allowed to cool to room temperature and *p*-methoxybenzyl alcohol/propargyl bromide (1 eq) was added. The solution was stirred at room temperature for 4–6 h, monitoring for completion using TLC and then poured into ice-cold water. The aqueous layer was extracted with EtOAc (3 × 30 mL) and the combined organic phases were dried over MgSO_4_ before removal of the solvent *in vacuo*.

- General Procedure for the Synthesis of the Azide (C)

This was adapted from a literature procedure [59]. To a suspension of *p*-anisidine/4-aminophenol (1 eq) in 4 M H_2_SO_4(aq)_ (30 mL) at 0 °C, a solution of NaNO_2_ (1.5 eq) in water (0.5 mL/mol), was added. The cloudy suspension was left stirring at 0 °C for 15 min open to air till all the solids had disappeared. A solution of NaN_3_ (1.5 eq) in water (0.5 mL/mol) was added very slowly with gas evolution. The brown solution was left stirring at room temperature for 3 h, followed by extraction with Et_2_O (3 × 50 mL). The combined organic phases were dried over MgSO_4_, before removal of the solvent *in vacuo* to give crude brown oil. Note: Azides should be handled with CAUTION as potentially EXPLOSIVE. Store below room temperature (18 °C) and protect from light.

- General Procedure for the Synthesis of the Triazole-Linked Biaryl Anilines and Ethers via Click Chemistry (D)

This was adapted from a literature procedure [60]. The propargyl analogue (1 eq), sodium ascorbate (0.5 eq) and CuSO_4_ (0.05 eq) were added to a solution of azide (1.5 eq) in *t*BuOH (1.0 mol/5 mL) and water (1 mol/5 mL). The cream suspension was stirred at room temperature overnight. The brown slurry was diluted with water (10 mL) and extracted with EtOAc (3 × 10 mL). The combined organic layers were dried over MgSO_4_ before the solvent was removed *in vacuo*.

- Purification by Precipitation (E)

Flash column chromatography was inadequate to obtain pure benzylphenyl ether and benzylphenyl aniline analogues; therefore, an additional purification (cold precipitation) was carried out. The required compound for purification was dissolved in the minimum amount of EtOAc required for complete dissolution, followed by the dropwise addition of hexane until precipitation took place. The precipitate was filtered and washed with the minimum amount of cold hexane to remove any soluble impurities, followed by drying of the residual precipitate under high vacuum to give the desired product.

### 3.3. InhA Enzyme Assay

The InhA:pET15a construct was kindly provided by Prof. Peter Tonge [61] (Stony Brook University, Stony Brook, NY, USA). This construct had the *InhA* gene attached downstream to the 6-His coding sequence. The protein was induced with 1 mM IPTG in a Luria Bertani (LB) broth (containing ampicillin 100 µg/mL), over-expressed in *E. coli* BL21-DE3 cells and grown at 30 °C for 6h until the OD_600_ reached 0.6. This was followed by extraction from the cell pellet and purification using a Ni-affinity His-Trap 5 mL column (GE Healthcare, Chicago, IL, USA). The column was washed with 150 mL binding buffer [Tris HCl (20 mM), NaCl (300 nM), imidazole (500 mM), pH 7.5] and the protein was eluted with elution buffer [Tris HCl (20 mM, NaCl, (300 nM), imidazole (500 mM), pH 7.5] in a 0–100% gradient of the elution buffer. The protein was analysed with SDS-PAGE and then exchanged to a storage buffer (PIPES, 30 mM, pH 6.8) and concentrated to 10 mg/mL using a 10 kDa Amicon centrifugal filter (Millipore, Watford, UK). Aliquots were then mixed with 10% glycerol and stored at −80°C. The molecular weight (29.806 Da) and pI (6.10) and extinction coefficient was determined using the ExPasy translate tool. The extinction co-efficient 37.5 mM^−1^cm^−1^ at λ_280_ was used in calculation of the protein concentration.

In vitro enzyme inhibition studies were carried out where the compounds were screened at a concentration of 100 μM using the thermostated UV-Vis. spectrophotometer (Varian Cary Bio 100, Agilent, Santa Clara, CA, USA) at 340 nm as described above. To overcome the problem of aqueous solubility of some of the compounds, the compound concentration was reduced to 50 or 25 μM.

The assay reaction conditions were as follows for a 400 μL volume: water (to volume), PIPES (30 mM), Inhibitor (varying concentrations), OCoA (400 μM) (see SI for synthesis), NADH (100 μM) and InhA (150 nM). All compounds were dissolved in DMSO. DMSO was found to cause inhibition of the enzyme and therefore its concentration in the final assay reaction was 1% *v*/*v*. The vehicle control consisted of water (to volume), PIPES (30 mM), DMSO (1%), OCoA (400 μM), NADH (100 μM) and InhA (150 nM).

To obtain the percentage of enzyme inhibition the initial velocity (v) for the first minute was established and this was compared to the velocity of the blank (v_0_). The initial velocity was calculated from the gradient of the absorbance vs time plots for each of the inhibitors and blanks. Inhibitory activities of the compounds were calculated as a percentage using the formula (1 − v/v_0_) × 100%, where v/v_0_ is the residual activity of the enzyme.

### 3.4. Minimum Inhibitory Concentration Determination

The MIC was calculated for some of the compounds. This was achieved using a Spot Culture Growth assay with *M. bovis* BCG, the vaccine strain for TB and a good model for drug screening [62,63]. Glycerol (0.2%) and 10% OADC was added to a 5 mL aliquot of Middlebrook 7H10 (BD Diagnostics, Franklin Lakes, NJ, USA) and placed in six-well microplate. 5 µL of the test compounds in a solution of DMSO at a concentration of 100 µM and 50 µM was added to the microplate. INH was used as a reference standard and was tested in the following concentrations 1, 0.5, 0.2, 0.1, 0.05, and 0 g/L. 5 µL of a mid-log phase (10^6^ cfu/mL) *M. bovis* BCG culture was added to each well. Results were obtained following an incubation period of two weeks at 37 °C. The MIC was determined as the lower concentration where there was no visually growth in the well. 

### 3.5. MIC Determination for Di-Triclosan Derivative ***39***

The measurement of the MIC_99_ was performed in 96-well flat bottom, black polystyrene microtiter plates (Greiner Bio-One, Kremsmünster, Austria) in a final volume of 200 μL. Two-fold drug dilutions in neat DMSO were performed and added to the microtiter plate at a final concentration of 1% *v*/*v* DMSO. DMSO (1% *v*/*v*) was used as a positive control and rifampicin as a negative control. The inoculum of *M. bovis* BCG culture was standardised at OD600 0.05 in Middlebrook 7H9 medium (Difco labs, Franklin Lakes, NJ, USA) and added to the plate. Plates were incubated without shaking at 37 °C, 5% CO_2_ for 7 days. Following incubation, 42 μL of resazurin (0.02% *v*/*v* in dH_2_O) was added to each well and incubated for a further 24 h. Fluorescence was measured using a Polarstar omega plate reader (BMG LABTECH SARL, Ortenberg, Germany) using λ_ex_ 544, λ_em_ 590 to determine the MIC value. 

## 4. Conclusions

The rational design and synthesis of a series of ligands (benzylphenyl, 1,4-triazole- linked diaryl, ether-linked *di*-triclosan) were based on TCS which is a known inhibitor of InhA. All molecules synthesized complied with Lipinski’s rule of 5 regarding good drug-like properties and oral availability except the *di*-triclosan analogue **31** which violates two of the rules. Compounds **13**, **38**, **39** and **40** which all have a ClogP > 5, should still have some oral availability as they only violate one of rules. All molecules fitted well into the active site when docked in silico with the *di*-triclosan analogues having the best fitness scores, followed by the triazole-linked analogues and the benzyl phenyl ether or aniline analogues. The isolated enzyme assays indicate that benzylphenyl aniline derivatives (**8**, **13**), 1,4-triazole-linked diaryl aniline analogues **21**, **23** and *di*-triclosan derivatives **31** and **38** display promise as possible leads. This inhibitory activity, however, requires further investigation as it does not correlate well with the whole cell activity suggesting that the compounds that are active against whole cells may have targets other than InhA. Preliminary biological screening reveals that there is evidence that some compounds in each group display moderate inhibitory activity. Compounds **12**, **39**, **43** and **44** had good MICs when tested against the whole cell bacteria. The differences observed between the MIC’s measured on whole cells and isolated enzyme assays suggest that compounds **12**, **39** and **44** may inhibit enzymes other than InhA in mycobacteria. 

In our recently published complementary study of *di*-triclosan analogues that incorporated a 1,4- or 1,5-triazole as a more rigid linker between the groups that should occupy the two triclosan binding sites in InhA [47], we identified two compounds, **45** which exhibited 100% inhibition of InhA at [50 µM] (MIC > 80 µM) and **46** which gave 11% inhibition at 50 µM (MIC 12.9 µM). These results also reflect a lack of correlation between the results from the isolated enzyme assays and the whole cell MIC values observed. Replacing the B-ring of the second TCS with a smaller structure as seen in compounds **39** and **46** does lead to a lower MIC value when compared with the ‘complete’ *di*-triclosans **38** and **45**, respectively. Given that these structures show comparable activity to TCS in the whole cell assays, it would be worth exploring which essential target(s) in *Mycobacterium tuberculosis* in addition to InhA, they may be inhibiting.

## Figures and Tables

**Table 1:** Summary of the physical properties of the compounds reported in this paper and their percentage inhibition of InhA, MIC, GOLD fitness scores and drug like properties for the benzyl phenyl analogues, triazole linked derivatives and *di*-triclosan analogues as well as intermediate compounds. Molecular weight (MW) Total polar surface area (TPSA), Hydrogen bond donors (HBD) and Hydrogen bond acceptors (HBA) are also reported.

Cpd	MW	ClogP	TPSA	HBD	HBA	Fitness Scores	% Inhibition	MIC (µg mL^−1^)
**TCS**	289.54	5.53	29.46	1	2	-	92	20 [48]
**INH**	137.14	−0.67	68.01	2	4	N/A	N/A	0.05
**8PP**	298.42	7.53	29.46	1	2	-	-	1.9 [28]
**1**	278.73	4.44 ± 0.27	27.70	0	3	57.22	NI	>50
**2**	262.28	3.70 ± 0.62	27.70	0	4	53.86	18	50
**3**	323.18	4.68 ± 0.52	27.70	0	3	58.66	25	>50
**8**	277.75	4.27 ± 0.56	30.50	1	3	60.28	63	>50
**12**	261.29	3.76 ± 0.60	30.50	1	4	55.62	NI	7.8
**13**	381.44	5.85 ± 0.80	21.71	0	5	69.25	43	>125
**20**	345.78	4.03 ± 0.82	58.42	0	6	75.59	36	>50
**21**	329.32	3.29 ± 1.05	58.42	0	7	71.55	56	>50
**22**	390.23	4.26 ± 0.94	58.42	0	6	75.80	NI	>50
**23**	344.80	3.86 ± 0.96	61.21	1	6	76.22	45	>50
**24**	328.34	3.34 ± 1.03	61.21	1	7	72.63	23	>125
**31**	511.39	8.21 ± 0.50	46.17	0	5	90.79	42	>125
**38**	483.34	7.88 ± 0.54	68.16	2	5	86.50	100	125
**39**	384.85	5.40 ± 0.48	27.70	0	4	72.92	27	7.8
**40**	450.05	6.01 ± 0.45	9.23	0	1	59.03	30	>50
**43**	354.80	3.50 ± 0.28	69.68	0	5	68.54	35	7.8
**44**	370.81	2.92 ± 0.26	78.92	0	6	62.44	14	15.6

All the compounds were tested at concentrations of 100 μM, with the exception of **1**, **3**, **20** and **39** and TCS (50 μM) and **22** (25 μM). NI (No Inhibition).

## Data Availability

Not applicable.

## Supplementary Materials

The following are available online at https://www.mdpi.com/article/10.3390/ph14040361/s1, Table S1: Fitness scores and rank list (high to low) for designed compounds and intermediates; Table S2: Summary of the interactions of the benzylphenyl analogues with the InhA active site.; Table S3: Summary of the interactions of the triazole linked analogues with the InhA active site.; Table S4: Summary of the interactions of the *di*-triclosan analogues with the InhA active site. Figure S1: Compounds tested. Figure S2: Interactions with the active site with benzylphenyl derivative 2. Figure S3: A view of the docking of 8 into the InhA active site. The active site residues (blue), NAD+ (magenta) and ligand (green). Figure S4: Docking of 23 into the InhA active site. Figure S5: A view of the docking of 31 into the InhA active site. The active site residues (blue), NAD+ (magenta) and ligand (green). Scheme S1: Synthesis of Octenyl CoA. Experimental details of compounds synthesised in this paper together with associated analytical data and NMR spectra.

## Abbreviations

CuAAC	Copper(I) Azide-Alkyne Cycloaddition
ENR	Enoyl reductase
XDR	Extensively drug-resistant
INH	Isoniazid
GOLD	Genetic Optimization for Ligand Docking
HBA	Hydrogen bond acceptors
HBD	Hydrogen bond donors
InhA	*M. tuberculosis* enoyl reductase
MDR	Multidrug-resistance
Mtb	*Mycobacterium tuberculosis*
NAD^+^	Nicotinamide adenosine dinucleotide
r.t.	Room temperature
SAR	Structure-activity relationship
TPSA	Topological polar surface area
TCS	Triclosan
TB	Tuberculosis
