# Supplementary material for: New InhA Inhibitors Based on Expanded Triclosan and Di-Triclosan Analogues to Develop a New Treatment for Tuberculosis
Source: Pharmaceuticals (Basel). 2021 Apr 14;14(4):361. doi: 10.3390/ph14040361 (PMC8070701; doi:10.3390/ph14040361)
Supplement: Supplementary file 1 [file pharmaceuticals-14-00361-s001.pdf]

Treating tuberculosis: New InhA inhibitors based on expanded triclosan and *di*-triclosan analogues

Supplementary Information

Author information:

Sarentha Chetty[1], Tom Armstrong[1], Shalu Sharma Kharkwal[1], William C. Drewe[1], Cristina de Matteis[2], Dimitris Evangelopoulos,[3] Sanjib Bhakta,[3] Neil R. Thomas[1*]

[1]Biodiscovery Institute, School of Chemistry, University of Nottingham, University Park, Nottingham NG7 2RD, UK

[2]School of Pharmacy, University of Nottingham, University Park, Nottingham NG7 2RD, UK

[3]Department of Biological Sciences, Birkbeck, University of London, London, WC1E 7HX, UK

Contents

Docking of designed ligands 2
Interaction with the active site residues 5
Synthesis 10
2-*trans*-Octenyl CoA 10
Experimental Section 10
NMR Data 36
References 75

Docking of designed ligands

A list of the docking results for all compounds including intermediates (Figure S1) is found in table S1.

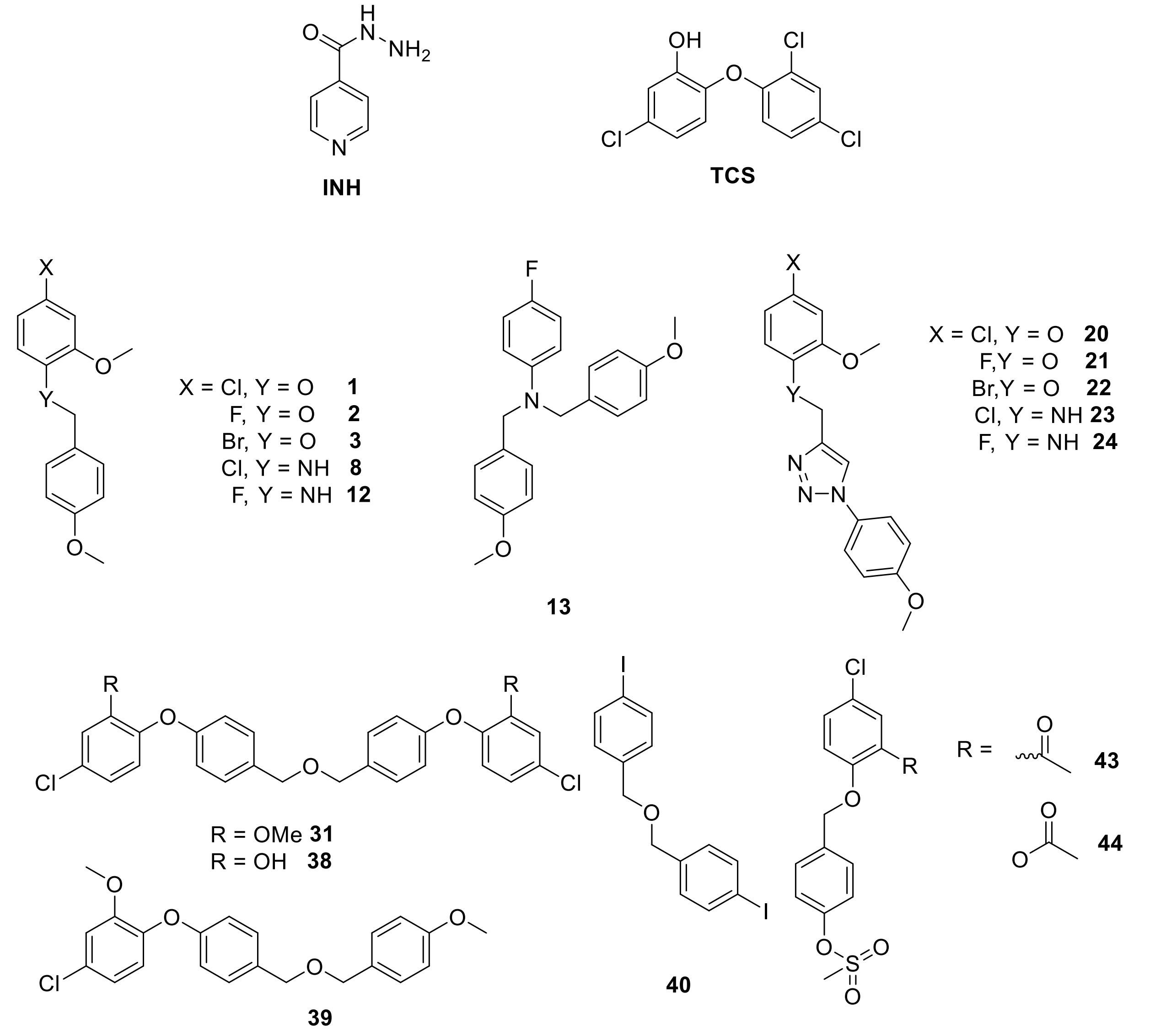

Figure S1 Compounds tested.

GOLD CONFIGURATION FILE

```
  AUTOMATIC SETTINGS
autoscale = 1

  POPULATION
popsiz = auto
select_pressure = auto
n_islands = auto
maxops = auto
niche_siz = auto
  GENETIC OPERATORS
pt_crosswt = auto
allele_mutatewt = auto
migratewt = auto
  FLOOD FILL
radius = 10
origin = 43.8601 51.7939 -82.835
do_cavity = 1
floodfill_atom_no = 0
cavity_file = /users/sarentha/2H7IcorA/cavity.atoms
floodfill_center = point

  DATA FILES
ligand_data_file /users/sarentha/molecules-designed2/cmpd1.mol2 10
ligand_data_file /users/sarentha/molecules-designed2/cmpd2.mol2 10
ligand_data_file /users/sarentha/molecules-designed2/compound_5.mol2 10
ligand_data_file /users/sarentha/SN2_analogues/SN2_M1.mol2 10
ligand_data_file /users/sarentha/SN2_analogues/SN2_M2.mol2 10
ligand_data_file /users/sarentha/SN2_analogues/SN2_M3.mol2 10
ligand_data_file /users/sarentha/SN2_analogues/SN2_M4.mol2 10
ligand_data_file /users/sarentha/SN2_analogues/SN2_M5.mol2 10
ligand_data_file /users/sarentha/SN2_analogues/SN2_M6.mol2 10
ligand_data_file /users/sarentha/SN2_analogues/SN2_M7.mol2 10
ligand_data_file /users/sarentha/SN2_analogues/SN2_M8.mol2 10
ligand_data_file /users/sarentha/SN2_analogues/SN2_M9.mol2 10
ligand_data_file /users/sarentha/SN2_analogues/SN2_M10.mol2 10
ligand_data_file /users/sarentha/Methylated_cmpds/me001.mol2 10
ligand_data_file /users/sarentha/Methylated_cmpds/me002.mol2 10
ligand_data_file /users/sarentha/Methylated_cmpds/me003.mol2 10
ligand_data_file /users/sarentha/Methylated_cmpds/me004.mol2 10
ligand_data_file /users/sarentha/Methylated_cmpds/me005.mol2 10
ligand_data_file /users/sarentha/Methylated_cmpds/me006.mol2 10
ligand_data_file /users/sarentha/Methylated_cmpds/me007.mol2 10
ligand_data_file /users/sarentha/Methylated_cmpds/me008.mol2 10
ligand_data_file /users/sarentha/Methylated_cmpds/me009.mol2 10
ligand_data_file /users/sarentha/Methylated_cmpds/me010.mol2 10
ligand_data_file /users/sarentha/Methylated_cmpds/me012.mol2 10
ligand_data_file /users/sarentha/Methylated_cmpds/me013.mol2 10
ligand_data_file /users/sarentha/Non_methyl_alt_mols/NMA001.mol2 10
ligand_data_file /users/sarentha/Non_methyl_alt_mols/NMA002.mol2 10
ligand_data_file /users/sarentha/Non_methyl_alt_mols/NMA003.mol2 10
param_file = DEFAULT
set_ligand_atom_types = 1
set_protein_atom_types = 0
directory = /users/sarentha/Final_Goldruns
tordist_file = DEFAULT
make_subdirs = 1
save_lone_pairs = 1
fit_points_file = fit_pts.mol2
read_fitpts = 0
  FLAGS
internal_ligand_h_bonds = 0
flip_free_corners = 0
match_ring_templates = 0
flip_amide_bonds = 0
flip_planar_n = 1 flip_ring_NRR flip_ring_NHR
```

flip_pyramidal_n = 0
rotate_carboxylic_oh = flip
use_tordist = 1
postprocess_bonds = 1
rotatable_bond_override_file = DEFAULT
solvate_all = 1
  TERMINATION
early_termination = 1
n_top_solutions = 3
rms_tolerance = 1.5
  CONSTRAINTS
force_constraints = 0
  COVALENT BONDING
covalent = 0
  SAVE OPTIONS
save_score_in_file = 1
save_protein_torsions = 1
  FITNESS FUNCTION SETTINGS
initial_virtual_pt_match_max = 3
relative_ligand_energy = 1
gold_fitfunc_path = consensus_score
start_vdw_linear_cutoff = 6
score_param_file = DEFAULT
docking_fitfunc_path = goldscore
docking_param_file = DEFAULT
rescore_fitfunc_path = chemscore
rescore_param_file = DEFAULT
  RUN TYPE
run_flag = CONSENSUS
  PROTEIN DATA
protein_datafile = /users/sarentha/2H7IcorA/______protein.mol2

Table S1 Fitness scores and rank list (high to low) for designed compounds and intermediates

Mol	Fitness	S(hb_ext)	S(vdw_ext)	S(hb_int)	S(int)
**31**	90.79	0.85	70.20	0.00	-7.97
**38**	86.50	0.27	65.31	0.00	-3.57
**23**	76.22	0.83	56.00	0.00	-1.61
**22**	75.80	2.74	56.47	0.00	-4.59
**20**	75.59	3.18	55.00	0.00	-3.21
**39**	72.92	0.77	58.14	0.00	-7.79
**24**	72.63	4.72	52.89	0.00	-4.81
**21**	71.55	4.07	51.52	0.00	-3.36
**43**	68.54	1.21	52.99	0.00	-5.53
**44**	62.44	1.59	47.91	0.00	-5.03
**8**	60.28	2.18	43.18	0.00	-1.28
**40**	59.03	0.00	40.14	0.00	59.03
**3**	58.66	0.00	44.99	0.00	-3.20
**1**	57.22	0.00	44.89	0.00	-2.59
**12**	55.62	0.00	43.42	0.00	-3.09
**2**	53.86	0.04	40.62	0.00	-2.04

Interaction with the active site residues

For the benzylphenyl ether/aniline target molecules, all the compounds displayed the correct orientation within the active site with the exception of **2** which had an inverted orientation. Compound **2** made an H-bond, in this case the lone pairs on the oxygen act as a HBA (Figure S2). This compound had an inverse orientation to what was expected.

Please note that the docked structures below include the lone-pairs on the hetero atoms of ligands.

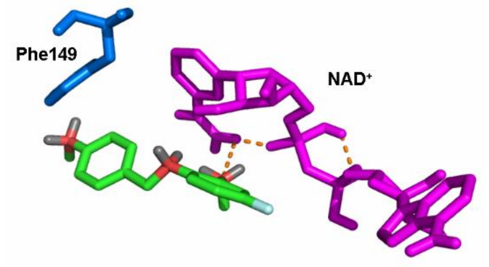

Figure S2 Interactions with the active site with benzylphenyl derivative 2.

The active site residues (blue), NAD^+ (magenta) and ligand (Atoms, C-green, O-red, H-white). Hydrogen bonds (orange).

Compound **8** had the best docking and inhibition data from the benzyl phenyl ether and benzyl phenyl aniline analogue series. It displayed 63 % inhibition in the isolated enzyme assay. Inspection of the docked structures, revealed that compound **8** (Figure 5) had an orientation similar to TCS, with a hydrogen bond occurring between the amine linker and the 2’-OH of the cofactor, where the amine acts as an H-bond donor. Beside H-bond interactions, a л-stacking interaction with NAD^+ and a van der Waals interaction with Phe149 (cation-л interaction) was also observed.

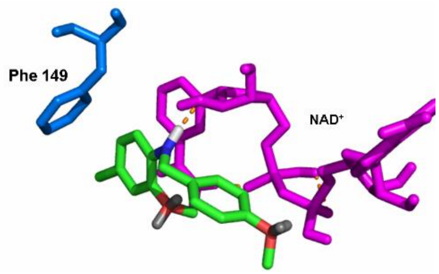

Figure S3. A view of the docking of **8** into the InhA active site. The active site residues (blue), NAD^+ (magenta) and ligand (green).

Mol	Fitness score	Tyr158[a]	Phe149[b]	NAD^{+}[a]	NAD^{+}[c]	Additional interactions
**12**	55.21	N	Y	Y	Y	N
**1**	57.22	N	Y	N	Y	N
**2**[e]	53.86	N	Y	Y	N	N
**3**	58.66	N	Y	N	Y	Leu218[d]
**8**	60.28	N	Y	Y	Y	N

Table S2 Summary of the interactions of the benzylphenyl analogues with the InhA active site.

[a] Hydrogen bond, [b] van der Waals interaction, [c] л-interaction, [d] hydrophobic interactions,

[e] Inverted orientation

Overall similar fitness scores were displayed for the triazole linked series of compounds. The compound that had the best docking results was **23** (Figure S3).

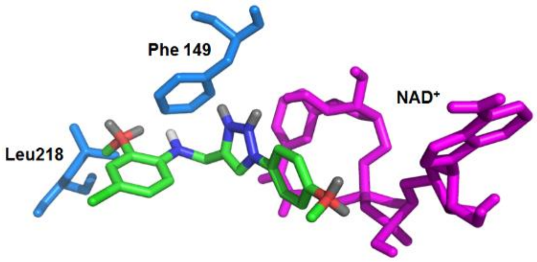

Figure S4 Docking of 23 into the InhA active site.
The active site residues (blue), NAD^{+} (magenta) and ligand.

A summary of the docking results is shown in Table S3.

Mol	Fitness score	Tyr158[a]	Phe149[b]	NAD[a]	NAD[c]	Additional interactions
**20**	75.59	N	Y	N	Y	Leu218[d]
**21**	71.55	N	Y	N	Y	Leu218[d]
**22**	75.80	N	Y	N	Y	Leu218[d]
**23**	76.22	N	Y	N	Y	Leu 218[d]
**24**	72.63	N	Y	Y	Y	Leu 218[d]

Table S3 Summary of the interactions of the triazole linked analogues with the InhA active site.

[a] Hydrogen bond, [b] van der Waals interaction, [c] л-interaction, [d] hydrophobic interactions.

Overall similar fitness scores were displayed for the bi-triclosan series of compounds. Compound **31** had the best fitness score (Figure S4).

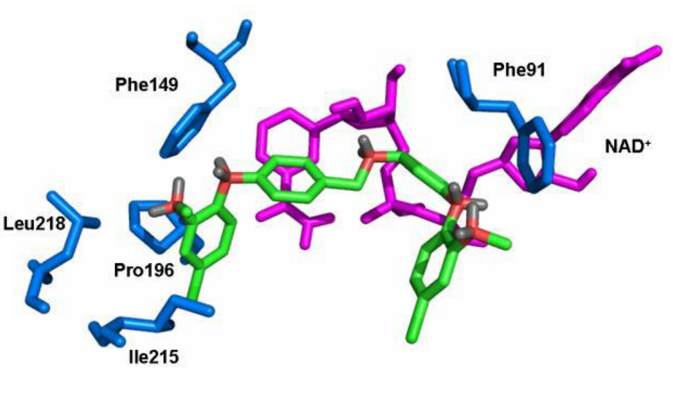

Figure S5. A view of the docking of **31** into the InhA active site. The active site residues (blue), NAD^+ (magenta) and ligand (green).

A summary of the docking results is shown in Table S4

Mol	Fitness score	Tyr158[a]	Phe149[b]	NAD[a]	NAD[c]	Additional interactions
**31**	90.79	N	Y	N	Y	Phe91[b], Leu218[d], Glu219 [d], Pro196[b],
**38**	86.50	N	Y	Y	Y	Leu218[d], Ile215[d]
**39**	72.92	N	Y	N	Y	Phe91[b]

Table S4 Summary of the interactions of the di-triclosan analogues with the InhA active site.

[a] Hydrogen bond, [b] van der Waals interaction, [c] л-interaction, [d] hydrophobic interactions

Synthesis

2-*trans*-Octenyl CoA

The substrate octenyl CoA (Scheme S1) was synthesized using a one pot synthesis using the peptide coupling agent PyBOP. This substrate was used in the isolated enzyme assays.

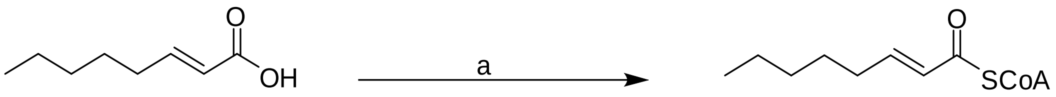

Scheme S1 Synthesis of Octenyl CoA

a) K_2CO_3, H_2O, CoA, THF, PyBOP, rt, 5hr, 38 %

Experimental Section

4-Chloro-2-methoxy-1-[(4-methoxybenzyl)oxy]benzene (1)

Following general procedure A (1), compound **1** was synthesised from 4-chloro-2-methoxybenzyl alcohol (1.00 g, 6.31 mmol) and *p*-methoxybenzyl chloride (2.96 g, 18.92 mmol). The cream crude product was purified by flash column chromatography EtOAc/Petrol (1:19 v/v), followed by further purification by precipitation using general method E, to give white crystals (0.52 g, 29 %); M.p. 92-94°C; IR ($CHCl_3$): V_{max} = 1250, 1515, 1249 cm^{-1}; ^1H NMR (400 MHz, $CDCl_3$): δ = 7.34 (2 H, d, *J* = 8.7 Hz, H-Ar), 6.89 (2 H, d, *J* = 8.7 Hz, H-AR), 6.86 (1 H, d, *J* = 1.8 Hz, H-Ar), 6.81 (2 H, m, H-Ar), 5.04 (2 H, s, CH_2), 3.86, 3.81 (2 x 3 H, s, 2 x CH_3); ^{13}C NMR (100 MHz, $CDCl_3$): δ = 159.5 (C–Ar), 150.4 (C-Ar), 146.9 (C-Ar), 129.1 (C-Ar), 128.1 (C-Ar), 126.2 (C-Ar), 120.3 (C-Ar), 115.1 (C-Ar), 113.98 (C-Ar), 112.5 (C-Ar), 71.2 (CH_2), 56.1, 55.3 (2 x CH_3), HRMS

(ESI) required for $C_{15}H_{15}{}^{35}ClO_3{}^+$ ($[MNa]^+$) m/z = 301.0607 found 301.0613; $C_{15}H_{15}ClO_3$, requires C, 64.64; H, 5.42 % found C, 64.39; H,5.39 %.

4-Fluoro-2-methoxy-1-[(4-methoxybenzyl)oxy]benzene (2)

Following general procedure A, (1) compound **2** was synthesized from 4-fluoro-2-methoxyphenol (0.30 g, 2.11 mmol) and *p*-methoxybenzylchloride (0.99 g, 6.34 mmol). Purification was achieved by flash column chromatography EtOAC/Petrol (1:19 v/v), followed by further purification by precipitation using general method E, to give white crystals (0.26 g, 47 %); M.p. 84-85 °C; IR ($CHCl_3$): V_{max} = 3007, 1514, 1034 cm^{-1}; ^1H NMR (400 MHz, $CDCl_3$): δ = 7.34 (2 H, d, *J* =8.7 Hz, H-Ar), 6.89 (2 H, d, *J* = 8.7 Hz, H-Ar), 6.80 (1 H, dd, *J* = 8.6 Hz, 6.4 Hz, H-Ar), 6.64 (1 H, dd, *J* = 6.4 Hz, 3.0 Hz), 6.52 (1 H, ddd, *J* = 8.6 Hz, 3.0 Hz, 1Hz), 5.01 (2 H, s, CH_2), 3.85, 3.81 (2 x 3 H, s, 2 x CH_3); ^{13}C NMR (100 MHz, $CDCl_3$): δ = 159.2 (C-Ar), 157.8 (d, *J* = 239.3 Hz, C-Ar), 150.8 (d, *J* = 9.9 Hz, C-Ar), 144.3 (C-Ar), 144.3 (d, *J* = 2.9 Hz, C-Ar), 129.2, 129.1, 115.3 (d, *J* = 22.5 Hz, C-Ar), 113.9 (C-Ar), 100.4 (d, *J* = 27.3 Hz, C-Ar), 71.8 (CH_2), 56.05, 55.29 (2 x CH_3); ^{19}F NMR (376 MHz, $CDCl_3$): δ = 120.1 (m, 1 F); HRMS (ESI) required for $C_{15}H_{15}FO_3{}^+$ ($[M+Na]^+$) *m/z* = 285.0903, found 285.0897; $C_{15}H_{15}FO_3$ requires C, 68.69; H, 5.76 % found C, 68.52; H, 5.75 %.

4-Bromo-2-methoxy-1-[(4-methoxybenzyl)oxy]benzene (3)

Following general procedure A, (1) compound **3** was synthesized from 4-bromo-2-methoxyphenol (0.30 g, 1.50 mmol) and *p*-methoxybenzylalcohol (0.69 g, 4.50 mmol). Purification was done by flash column chromatography EtOAc/Petrol (1:19 v/v), followed by further purification by precipitation using general method E to give white crystals (0.11 g, 23 %); M.p. 110-112 °C; IR

($CHCl_3$): V_{max} = 3008, 1615, 1586, 1248 cm^{-1}; ^1H NMR (400 MHz, $CDCl_3$): δ = 7.34 (2 H, d, *J* = 8.6 Hz), 6.99 (1 H, d, *J* = 2.5 Hz, H-Ar), 6.96 (1 H, dd, *J* = 8.5 Hz, 2.5 Hz, H-Ar), 6.89 (2 H, d, *J* = 8.6 Hz, H-Ar), 6.74 (1 H, d, *J* = 8.5 Hz, H-Ar), 5.04 (2 H, s, CH_2), 3.86, 3.81 (2 x 3 H, s, 2 x CH_3); ^{13}C NMR (100 MHz, $CDCl_3$): δ = 159.5 (C-Ar), 150.6 (C-Ar), 147.4 (C-Ar), 129,1 (C-Ar), 128.8 (C-Ar), 123.3 (C-Ar), 115.6 (C-Ar), 115.3 (C-Ar), 113.9 (C-Ar), 113.3 (C-Ar), 71.1 (CH_2), 56.1, 55.3 (CH_3); HRMS (ESI) required for $C_{15}H_{15}{}^{79}BrO_3{}^+$ ($[MNa]^+$) *m/z* = 345.0102, found 345.0103.

tert-Butyl(4-chloro-2-hydroxyphenyl)carbamate (5)

A literature procedure (2) was modified to synthesise compound **5**. To a suspension of 2-amino-5-chlorophenol (1.50 g, 10.50 mmol) in dioxane (30 mL), Boc anhydride (2.41 g, 11.00 mmol) was added. The solution was cooled to 0 °C, followed by the dropwise addition of a solution of $NaHCO_3$ (3.5 g, 41.7 mmol) in H_2O (30 mL). The brown solution was allowed to warm to room temperature and stirred overnight. Water (30 mL) was added and the solution was extracted with DCM (3 x 30 mL). The combined organic layers were washed with 1M $HCl_{(aq)}$ (100 mL), brine (100 mL), and dried over $MgSO_4$, before the solvent was removed *in vacuo*. The dark orange crude product was purified by flash column chromatography with EtOAc/Petrol (1:19-1:9 v/v) to give a pale orange solid (1.44 g, 56 %); M.p. 136-138 °C; IR ($CHCl_3$): V_{max} = 3630, 1691, 1515, 1157 cm^{-1}; ^1H NMR (400 MHz, $CDCl_3$): δ = 8.42 (1 H, s, NH), 6.96 (2 H, m, H- Ar H-Ar), 6.82 (1 H, dd, 8.4 Hz, 2.3 Hz, H- Ar), 6.59 (1 H, s, OH), 1.53 (9 H, s, CH_3); ^{13}C NMR (100 MHz, $CDCl_3$): δ = 155.0 (C-Ar), 148.4 (C-Ar), 130.52 (C-Ar), 124.3 (C-Ar), 122.13 (C-Ar), 120.67 (C-Ar), 119.23 (C-Ar), 82.59, 28.19 (CH_3); HRMS (ESI) required for $C_{11}H_{14}{}^{35}ClNO_4{}^+$ ($[MNa]^+$) *m/z* =266.0560, found 266.0547.

tert-Butyl (4-chloro-2-methoxyphenyl)carbamate (6)

A literature procedure (3, 4) was modified to synthesise compound **6**. Under a nitrogen atmosphere K_2CO_3 (4.08 g, 29.60 mol) and iodomethane (7.37 g, 51.10 mmol) were added to a solution of the Boc protected aniline **5** (1.44 g, 5.90 mmol) in anhydrous acetone (25 mL). The suspension was heated to reflux for 6.5 h then allowed to cool to room temperature, followed by the addition of saturated $NH_4Cl_{(aq)}$ (20 mL). The solvent was removed by evaporation and the mixture was extracted with EtOAc (2 x 20 mL). The combined organic layers were washed with water (100 mL) and dried over $MgSO_4$, and the solvent was removed *in vacuo*. Purification of a portion of the crude (100 mg) by flash column chromatography EtOAc/Petrol (1:9 v/v) gave a yellow oil (0.88 g, 82 %); IR ($CHCl_3$): V_{max} = 3432, 3010, 1722, 1517, 1247, 1156 cm^{-1}; ^1H NMR (400 MHz, $CDCl_3$): δ = 8.01 (1 H, d, *J* = 8.5 Hz, H-Ar), 6.99 (1 H, s, NH), 6.91 (1 H, dd, *J* = 8.5 Hz, 2.2 Hz, H- Ar), 6.82 (1 H, d, *J* = 2.2 Hz, H-Ar), 3.858 (3H, s, CH_3), 1.517 (9 H, s, CH_3); ^{13}C NMR (100 MHz, $CDCl_3$): δ = 152.6 (C=O), 147.9 (C-Ar), 127.1 (C-Ar), 126.8 (C-Ar), 120.8 (C-Ar), 118.7 (C-Ar), 110.7 (C-Ar), 80.60 (C-8), 55.89 (C-10), 28.34 (CH_3); HRMS (ESI) required for $C_{12}H_{16}{}^{35}ClNO_3^+$ ($[MNa]^+$) *m/z* =280.0716, found 280.0703.

4-Chloro-2-methoxyaniline (7)

A literature procedure(5, 6) was modified to synthesise compound **7**. To a solution of **6** (1.38 g, 5.40 mmol) in DCM (0.8 mL) cooled to 0 °C, TFA (1.19 g, 10.7 mmol) was added slowly dropwise *via* a syringe. The brown solution was allowed to warm to room temperature and left stirring for 2 h. The solvent and TFA were removed by evaporation. Purification by flash column chromatography EtOAc/Petrol (1:9-3:7 v/v) gave a brown oil (0.71 g, 85 %); IR ($CHCl_3$): V_{max} = 3459, 1504, 1614, 1584, 1278, 880 cm^{-1}; ^1H NMR (400 MHz, $CDCl_3$): δ = 6.77 (2 H, m,

H-Ar, H-Ar), 6.60 (1 H, d, $J = 8.8$ Hz, H-Ar), 3.84 (3 H, s, CH_3) which is consistent with the literature(7); ^{13}C NMR (100 MHz, $CDCl_3$): δ = 148.0 (C-Ar), 133.8 (C-Ar), 123.6 (C-Ar), 115.8 (C-Ar), 111.15 (C-Ar), 55.7 (CH_3); HRMS (ESI) required for $C_7H_8{}^{35}ClNO^+$ ($[MH]^+$) m/z = 158.0373, found 158.0367.

4-Chloro-2-methoxy-*N*-(4-methoxybenzyl) aniline (8)

To a solution of aniline **7** (0.20 g, 1.3 mmol) in anhydrous DCM (13 mL) cooled to 0 °C, triethylamine (0.26 g, 2.5 mmol) was added dropwise *via* a syringe. The solution was stirred at 0 °C for 10 min followed by dropwise addition of *p*-methoxybenzyl chloride (0.27 g, 1.3 mmol). The solution was allowed to warm to room temperature and was stirred for 5.5 h, followed by the addition of saturated $NH_4Cl_{(aq)}$ solution (15 mL). The phases were separated and the organic phase was then washed with water (15 mL) and brine (15 mL). The combined aqueous phases were extracted with DCM (2 x 30 mL). The combined organic phases were dried over $MgSO_4$, before the solvent was removed *in vacuo*. Purification by flash column chromatography EtOAc/Petrol (1:19 v/v), gave a waxy cream solid (43.2 mg, 12 %); M.p. 97-98 °C, IR ($CHCl_3$): V_{max} = 3062, 1512, 1246; ^1H NMR (400 MHz,$CDCl_3$): δ = 7.31 (2 H, d, $J = 8.7$ Hz, H-Ar), 6.92 (2 H, d, $J = 8.7$ Hz, H-Ar), 6.82 (1 H, dd, $J = 8.6$ z, 2.2 Hz, H-Ar), 6.77 (1 H, d, $J = 2.2$ Hz, H-Ar), 6.51 (1 H, d, $J = 8.6$ Hz, H-Ar), 4.53 (1 H, s, NH), 4.28 (2 H, s), 3.86, 3.84 (2 x 3 H, s); ^{13}C NMR (100 MHz, $CDCl_3$): δ = 158.9 (C-Ar), 147.1 (C-Ar), 136.8 (C-Ar), 131.11 (C-Ar), 128.8 (C-Ar), 121.1 (C-Ar), 120.8 (C-Ar), 114.1 (C-Ar), 110.33 (C-Ar), 110.13 (C-Ar), 55.7, 55.3 (2 x CH_3); 47.5 (CH_2); HRMS (EI) required for $C_{15}H_{16}{}^{35}ClNO_2^+$ m/z = 277.0870 found 277.0864.

4-Fluoro-2-methoxy-1-nitrobenzene (10)

A literature procedure (3, 4) was modified to synthesise compound **10**. Under a nitrogen atmosphere, K_2CO_3 (13.20 g, 95.5. mol) and iodomethane (27.11 g, 190.9 mmol) were added to a solution of compound **9** (3 g, 19.0 mmol) in anhydrous acetone (81 mL). The mixture was heated to reflux for 5 h. The yellow solution was allowed to cool to room temperature, followed by the addition of saturated $NH_4Cl_{(aq)}$ solution (100 mL). The solution was extracted with EtOAc (3 x 100 mL). The combined organic layers were washed with water (100 mL) and dried over $MgSO_4$, before the solvent was removed *in vacuo*. Purification by flash column chromatography EtOAc/Petrol (1:4 v/v) gave a bright yellow powder (2.64 g, 81 %); M.p. 97-98 °C; IR ($CHCl_3$): V_{max} = 1623, 1527, 1352, 1291 cm^{-1} which is consistent with literature values(8); ^{1}H NMR (400 MHz, $CDCl_3$): δ = 7.97 (1 H, app. dd, *J* = 9.0, 5.9 Hz, H-Ar), 6.79 (1 H, app. dd, *J* = 10.2, 2.5, H-Ar), 6.73 (1 H, m, H-Ar), 3.97 (3 H, s, CH_3); ^{13}C NMR (100 MHz, $CDCl_3$): δ = 165.8 (d, *J* = 256.1 Hz, C-Ar), 155.3 (d, *J* = 11.3 Hz, C-Ar), 135.9 (C-Ar), 128.2 (d, *J* = 11.3 Hz, C-Ar), 107.3 (d, *J* = 23.6 Hz, C-Ar), 56.8 (CH_3); ^{13}C and ^{1}H NMR data is consistent with literature values considering difference in chemical shifts due to the usage of a different solvent (acetone).(8) ^{19}F NMR (376 MHz): δ = -100.4 (1 F); HRMS (ESI) required for $C_7H_6FNO_3^+$ ($[MNa]^+$) *m/z* = 194.0229, found *m/z* = 194.0209.

4-Fluoro-2-methoxyaniline (11)

A literature procedure (9) was modified to synthesise compound **11**. To a solution of **10** (2.30 g, 13.44 mmol) in MeOH (230 mL), ammonium formate (8.48 g, 13.4 mmol) was added followed by the addition of 10 % Pd/C catalyst (0.23 g, 0.1 w/w equiv) (added very slowly with **CAUTION**). The solution was allowed to stir overnight at room temperature then filtered through a bed of celite.

The solvent was removed by evaporation and the residue was dissolved in chloroform (10 mL) and washed with 5 % aqueous ammonia (3 x 10 mL) and brine (3 x 10 mL). The organic layer was dried over $MgSO_4$, before the solvent was removed *in vacuo* to give an orange oil (1.90 g, 13.4 mmol, 100 %); IR ($CHCl_3$): V_{max} = 3452, 3369, 1614, 1591, 1034, 946.29, 834 cm^{-1} which is consistent with literature values(10); ^1H NMR (400 MHz,$CDCl_3$): δ = 6.21 (1 H, dd, *J* = 8.5 , 5.7 Hz, H-Ar), 6.56 (1H, dd, *J* = 2.6 Hz, 10.3 Hz, H-Ar), 6.49 (1 H, m, H-Ar), 3.84 (3 H, s, CH_3), 3.62 (2 H, s, NH_2) which is consistent with literature values(7); ^{13}C NMR (100 MHz, $CDCl_3$): δ = 156.4 (d, *J* = 236.2 Hz, C-Ar), 147.8 (d, *J* = 9.5 Hz, C-Ar), 131.9 (C-Ar), 114.7 (d, *J* = 9.5 Hz, C-Ar), 106.4 (d, *J* = 21.9 Hz, C-Ar), 98.6 (d, *J* = 26.7 Hz, C-Ar), 55.7 (CH_3) which is consistent with literature values(11); ^{19}F NMR (376 MHz): δ = -124.2 (1 F); HRMS (ESI) required for $C_7H_7FNO^+$ ($[MH]^+$) *m/z* = 142.0668, found *m/z* = 142.0654.

4-Fluoro-2-methoxy-*N*-(4-methoxybenzyl) aniline (12)

Following general procedure B,(12) compound **12** was synthesised from aniline **11** (0.30 g, 2.03 mmol) and *p*-methoxybenzylchloride (0.33 g, 2.1 mmol). Purification was performed in two steps with initial purification by flash column chromatography EtOAc/Petrol (1:9 to 3:7 v/v) to give a waxy cream solid which was further purified by precipitation using general method E to give white crystals (0.08 g, 14 %); M.p. 96-97 °C; IR ($CHCl_3$): V_{max} = 3007, 1515, 1249, 1035 cm^{-1}; ^1H NMR (400 MHz, $CDCl_3$): δ = 7.29 (2 H, d, *J* = 8.7 Hz, H-Ar), 6.88 (2 H, d, *J* = 8.7 Hz, H-Ar), 6.52 (3 H, m,3 x H-Ar), 4.33 (1 H, s, NH), 4.24 (2 H, s, CH_2), 3.82, 3.81 (2 x 3 H, s, 2 x CH_3); ^{13}C NMR (100 MHz, $CDCl_3$): δ = 157.7 (d, *J* = 226.6 Hz, C-Ar), 154.26 (C-Ar), 147.3 (d, 9.5 Hz, C-Ar), 134.4 (C-Ar), 131.4 (C-Ar), 128.8 (C-Ar), 113.9 (C-Ar), 109.7 (d, *J* = 9.0 Hz, C-Ar), 106.1 (d, *J* = 21.5 Hz, C-Ar), 98.5 (d, *J* = 27.2 Hz, C-Ar), 55.6, 55.30 (2 x CH_3), 47.9 (CH_2); ^{19}F NMR

(376 MHz): δ = 126.4 (1 F) HRMS (ESI) required for $C_{15}H_{16}FNO^+$ ($[MH]^+$) *m/z* = 262.1243, found *m/z* = 262.1231.

4-Fluoro-2-methoxy-*N*-(4-*bis*-methoxybenzyl) aniline (13)

Following general method B, (12) compound **13** was synthesised from aniline **11** (0.30 g, 2.03 mmol) and *p*-methoxybenzylchloride (0.99 g, 6.38 mmol). Purification was done in two steps with initial purification by flash column chromatography EtOAc/Petrol (1:9 to 3:7 /vv) gave a yellow oil, followed by a second purification by flash column chromatography EtOAc/Petrol (1:9 v/v) gave a colourless oil (0.29 g, 36 %); IR ($CHCl_3$): V_{max} = 3009, 1611, 1511, 1247, 1035 cm^{-1}; ^{1}H NMR (400 MHz, $CDCl_3$): δ = 7.15 (4 H, d, *J* = 8.8 Hz, H-Ar), 6.79 (4 H, d, *J* = 8.8 Hz, H-Ar), 6.63 (2 H, m, 2 x H-Ar), 6.42 (1 H, m, H-Ar), 4.08, (4 H, s, CH_2), 3.90 (3 H, s, CH_3), 3.77 (6 H, s, CH_3); ^{13}C NMR (100 MHz, $CDCl_3$): δ = 159.0 (d, *J* = 240.0 Hz, C-Ar), 154.5 (d, *J* = 9.9 Hz, C-Ar), 135.6 (d, *J* = 3.1 Hz, C-Ar), 130.8 (C-Ar), 129.7 (C-Ar), 122.9 (d, *J* = 9.9 Hz, C-Ar), 113.4, 105.9 (d, *J* = 21.5 Hz), 99.9 (d, *J* = 26.3 Hz), 55.7 (2 x CH_2, 2 x CH_3), 55.2 (CH_3); ^{19}F NMR (376 MHz): δ = -118.9 (m, 1 F); HRMS (ESI) required for $C_{23}H_{24}FNO_3^+$ ($[MH]^+$) *m/z* = 382.1818, found *m/z* = 382.1816.

4-Chloro-2-methoxy-1-(prop-2-yn-1-yloxy) benzene (14)

Following general method B, (12) compound **14** was synthesised from 4-chloro-2-methoxyphenol (1.0 g, 7.20 mmol) and propargyl bromide (1.06 g, 8.70 mmol) Purification was by flash column chromatography EtOAc/Petrol (1:9 v/v), to give white crystals (1.07 g, 75 %); M.p. 43-44°C; IR ($CHCl_3$): V_{max} = 3307, 3011, 1594, 1252 cm^{-1}; ^{1}H NMR (400 MHz, $CDCl_3$): δ = 6.95 (1 H, m, H-Ar), 6.88 (2 H, m, H-Ar), 4.73 (2 H, d, 2.4 Hz, CH_2), 3.86 (s, 3 H, H-10), 2.51 (t, 2.4 Hz, 1 H,

CH); ^{13}C NMR (100 MHz, $CDCl_3$): δ = 150.4 (C-Ar), 145.5 (CH), 127.2 (C-Ar), 120.2 (C-Ar), 115.5 (C-Ar), 112.5 (C-Ar), 78.2 (C-Ar), 76.1 (CH), 57.1 (CH_2), 56.1 (CH_3); HRMS (EI) required for $C_{10}H_9{}^{35}ClO_2^+$ *m/z* = 196.0291, found 196.0279.

4-Fluoro-2-methoxy-1-(prop-2-yn-1-yloxy) benzene (15)

Following general method B, (12) compound **15** was synthesised from 4-fluoro-2-methoxyphenol (0.50 g, 3.5 mmol) and propargyl bromide (0.50 g, 4.2 mmol). Purification by flash column chromatography EtOAc/Petrol (1:9 v/v), gave a white solid (0.38 g, 75 %); M.p. 36-37 °C; IR ($CHCl_3$): V_{max} = 3308, 3010, 1612, 1506, 1467, 1192 cm^{-1}; ^{1}H NMR (400 MHz, $CDCl_3$): δ = 6.97 (1 H, dd, *J* = 8.8 Hz, H-Ar), 6.65 (1 H, dd, *J* = 2.8 Hz, 10.1 Hz, H-Ar), 6.59 (1 H, m, H-Ar), 4.71 (2 H, d, *J* = 2.4 Hz, CH_2), 3.85 (3 H, s, CH_3), 2.49 (1 H, t, *J* = 2.4 Hz, CH) which are consistent with literature values(15); ^{13}C NMR (100 MHz, $CDCl_3$): δ = 158.4 (d, *J* = 240.0 Hz , C-Ar), 150.9 (d, *J* = 9.9 Hz, C-Ar), 142.9 (d, *J* = 2.6 Hz, C-Ar), 116.0 (d, *J* = 9.9 Hz, C-Ar), 105.9 (d, *J* = 22.7 Hz, C-Ar), 100.4 (d, 24.5 Hz, C-Ar), 78.6 (CH), 75.8, 57.6 (CH_2), 56.0 (CH_3); ^{19}F NMR (376 MHz, $CDCl_3$): -118.79 (m, 1 F); HRMS (EI) required for $C_{10}H_9FO_2^+$ *m/z* = 180.0587, found *m/z* = 180.0587; $C_{10}H_9FO_2$ requires C, 66.66, H, 5.03 %, found C, 66.71 %; H, 5.03 %.

4-Bromo-2-methoxy-1-(prop-2-yn-1-yloxy) benzene (16)

Following general method B, (12) compound **16** was synthesised from 4-bromo-2-methoxyphenol (0.50 g, 2.50 mmol) and propargyl bromide (0.35 g, 2.90 mmol). Purification by column chromatography EtOAc/Petrol (1:9 v/v), gave a cream solid (0.43 g, 71 %); M.p. 43-44 °C, IR ($CHCl_3$): V_{max} = 3307, 3011, 1592, 1501, 1250 cm^{-1}; ^{1}H NMR (400 MHz, $CDCl_3$): δ = 7.02 (2 H, m, H-Ar, H-Ar), 6.91 (1 H, d, *J* = 8.4 Hz, H-Ar), 4.74 (2 H, d, *J* = 2.4 Hz, CH_2), 3.86 (3 H, s, CH_3),

2.51 (1 H, t, *J* = 2.4 Hz, CH); ^{13}C NMR (100 MHz, $CDCl_3$): δ = 150.5 (C-Ar), 145.9 (C-Ar), 123.3 (C-Ar), 123.3 (C-Ar), 115.9 (C-Ar), 115.3 (C-Ar), 114.4 (C-Ar), 78.1, 76.1 (CH), 56.9 (CH_2), 56.1 (CH_3); HRMS (EI) required for $C_{10}H_9{}^{79}BrO_2^+$ *m/z* = 239.9786, found $[C_{10}H_9O_2]^+$ $[M^+\text{-}Br^-]$, *m/z* = 161.0587; $C_{10}H_9BrO_2$ requires C, 49.82; H, 3.76 % found C, 49.99, H, 3.77 %.

4-Chloro-2-methoxy-*N*-(prop-2-yn-1-yl) aniline (17)

Following general method B, (12) compound **17** was synthesised from 4-chloro-2-methoxyaniline (0.30 g, 1.9 mmol) and propargyl bromide (0.27 g, 2.3 mmol). Purification by flash column chromatography EtOAc/Petrol (1:9 v/v) gave a yellow oil (0.09 g, 71 %); IR ($CHCl_3$): V_{max} = 3461, 3377, 3008, 1613, 1505, 1278, 879 cm^{-1}; ^{1}H NMR (400 MHz, $CDCl_3$): δ =6.87 (1 H, dd, *J* = 8.4 Hz, 2.2 Hz, H-Ar), 6.76 (1 H, d, *J* = 2.2 Hz, H-Ar), 6.59 (1 H, d, *J* = 8.4 Hz, H-Ar), 4.42 (1 H, bs, NH), 3.94 (2 H, s, CH_2), 3.84 (3 H, s, CH_3), 2.21 (1 H, t, *J* = 2.5 Hz, CH); ^{13}C NMR (100 MHz, $CDCl_3$): δ = 147.7 (C-Ar), 135.4 (C-Ar), 120.7 (C-Ar), 111.1 (C-Ar), 110.4 (C-Ar), 80.7, 71.3 (CH), 55.7 (CH_3), 33.2 (CH_2); HRMS (EI) required for $C_{10}H_{10}{}^{35}ClNO^+$ *m/z* = 195.0451, found 195.0441.

4-Fluoro-2-methoxy-*N*-(prop-2-yn-1-yl) aniline (18)

Following general procedure B, (12) compound **18** was synthesised from 4-fluoro-2-methoxyaniline (0.30 g, 2.10 mmol) and propargyl bromide (0.31 g, 2.50 mmol). Purification was by flash column chromatography EtOAc/Petrol (1:9 v/v), followed by a second purification of a portion (0.072 g) of the crude by column chromatography DCM/Petrol (2:8 v/v) to give a yellow oil (0.06 g, 30 %); IR ($CHCl_3$): V_{max} = 3425, 3307, 3011, 1610, 1518, 1454, 1252, 1119 cm^{-1}; ^{1}H NMR (400 MHz, $CDCl_3$): δ = 6.59 (3 H, m, H-Ar), 4.25 (1 H, bs, NH), 3.94 (2 H, d, *J* = 2.4 Hz,

CH_2), 3.83 (3 H, s, H-10), 2.20 (1 H, t, *J* = 2.4 Hz, H-9); ^{13}C NMR (100 MHz, $CDCl_3$): δ = 156.1 (d, *J* = 235 Hz, C-Ar), 147.9 (d, *J* = 9.5 Hz, C-Ar), 132.9 (C-Ar), 110.7 (d, *J* = 9.3 Hz, C-Ar), 106.2 (d, *J* = 21.5 Hz, C-Ar), 98.7 (d, *J* = 27.2 Hz, C-Ar), 81.0, (C), 81.2 (CH_3), 55.7 (CH), 33.7 (CH_2); ^{19}F NMR (376 MHz, $CDCl_3$): -124.9 (m, 1 F); HRMS (EI) required for $C_{10}H_{10}FNO^+$ *m/z* = 179.0746, found 179.0739.

1-Azido-4-methoxybenzene (19)

Following general procedure C, (13) compound **19** was synthesised from *p*-methoxyaniline, $NaNO_2$ (2.52 g, 36.6 mmol) and NaN_3 (2.38 g, 36.60 mmol). Purification by column chromatography EtOAc/Petrol (1:9 v/v) gave a brown solid (2.99 g, 82 %); M.p. 40-42 °C; IR (KBr): V_{max} = 2106, 1505, 1245 cm^{-1}; ^{1}H NMR (400 MHz,$CDCl_3$): δ = 6.94 (2 H, d, *J* = 9.0 Hz, 2 x H-Ar), 6.89 (2 H, d, *J* =9.0 Hz, H-Ar), 3.79 (3 H, s, CH_3), which is consistant with literature values(14); ^{13}C NMR (100 MHz, $CDCl_3$): δ = 156.9 (C-Ar), 132.3 (C-Ar), 119.9 (C-Ar), 115.1 (C-Ar), 55.5 (CH_3).

4-[(4-Chloro-2-methoxyphenoxy) methyl]-1-(4-methoxyphenyl)-1-*H*-1,2,3 triazole (20)

Following general method D, (9) compound **20** was synthesised from the propargyl analogue **14** (0.10 g, 0.6 mmol) and azide **19** (0.25 g, 1.7 mmol). Purification was done by flash column chromatography EtOAc/Petrol [1:9 v/v to EtOAc/MeOH (one drop)] to give an orange powder (0.14 g, 74 %); M.p. 144-145 °C; IR ($CHCl_3$): V_{max} = 3009, 1519, 1503, 1254, 1037, 834 cm^{-1}; ^{1}H NMR (400 MHz, $CDCl_3$): δ = 7.99 (1 H, s, CH), 7.61 (2 H, d, *J* = 9.0 Hz, H-Ar), 7.0 (2 H, m, 2 x H-Ar), 6.87 (2 H, m, 2 x H-Ar), 5.32 (2 H, s, CH_2), 3.86 (2 x 3H, s, 2 x CH_3), ^{13}C NMR (100 MHz, $CDCl_3$): δ = 159.9 (C-Ar), 150.2 (C-Ar), 146.3 (C-Ar), 130.4 (C-Ar), 126.8 (C-Ar), 122.28 (C-

Ar), 121.4 (CH), 120.4 (C-Ar), 115.1 (C-Ar), 114.8 (C-Ar), 112.5 (C-Ar), 63.3 (CH_2), 56.1, 55.6 (2 x CH_3); HRMS (ESI) required for $C_{17}H_{16}{}^{35}ClN_3O_3^+$ ($[MNa]^+$) *m/z* = 368.0778, found *m/z* = 368.0768.

4-[(4-Fluoro-2-methoxyphenoxy) methyl-1-(4-methoxyphenyl)-1*H*-1,2,3-triazole (21)

Following general method D, (9) compound **21** was synthesised from propargyl analogue **15** (0.20 g, 1.2 mmol) and azide **19** (0.54 g, 3.6 mmol) Purification by flash column chromatography EtOAc/Petrol [(1:9 v/v) to EtOAc/MeOH (one drop)] gave an orange powder (0.14 g, 77 %); M.p. 127-128 °C; IR ($CHCl_3$): V_{max} = 3087, 1612, 1466, 1519, 1035, 835 cm^{-1}; ^1H NMR (400 MHz, $CDCl_3$): δ = 8.01 (1 H, s, CH), 7.64 (2 H, d, *J* = 9.0 Hz, H-Ar), 7.03 (3 H, m, H-Ar), 6.68 (1 H, dd, *J* = 10.1 Hz, 2.9 Hz, H-Ar), 6.61 (1 H, m, H-Ar), 5.33 (2 H, s, CH_2), 3.89, 3.86 (2 x 3 H, s, 2 x CH_3); ^{13}C NMR (100 MHz, $CDCl_3$): δ = 159.9 (C-Ar), 158.1 (d, *J* = 240.0 Hz, C-Ar), 150.7 (d, *J* = 9.9 Hz, C-Ar), 143.8 (d, *J* = 3.0 Hz, C-Ar), 130.6 (C-Ar), 122.3 (CH), 115.4 (d, *J* = 9.9 Hz, C-Ar), 106.0 (d, *J* = 22.7 Hz, C-Ar), 100.7 (d, *J* = 27.4 Hz, C-Ar), 63.9 (CH_2), 56.1, 55.6 (2 x CH_3); ^{19}F NMR (376 MHz, $CDCl_3$): -119.3 (m, 1 F); HRMS (ESI) required for $C_{17}H_{16}FN_3O_3^+$ ($[MNa]^+$) *m/z* = 352.1073, found *m/z* = 352.1058.

4-[(4-Bromo-2-methoxyphenoxy) methyl-1-(4-methoxyphenyl)-1*H*-1,2,3-triazole (22)

Following general method D, (9) compound **22** was synthesised from propargyl analogue **16** (0.20 g, 0.9 mmol) and azide **19** (0.39 g, 2.6 mmol). Purification by flash column chromatography EtOAc/Petrol (1:9v/v) to EtOAc, gave an orange powder (0.29 g, 83 %); M.p. 145-146 °C; IR ($CHCl_3$): V_{max} = 3012, 1680, 1465, 1519, 1252 cm^{-1}; ^1H NMR (400 MHz, $CDCl_3$): δ = 7.97 (1 H, s, CH), 7.61 (2 H, d, *J* = 6.9 Hz, H-Ar), 6.99 (4 H, m, H-Ar), 5.33 (2 H, s, CH_2), 3.86 (6 H, s, 2 x

CH_3); ^{13}C NMR (100 MHz, $CDCl_3$): δ = 159.9 (C-Ar), 150.33 (C-Ar), 146.8 (C-Ar), 144.4 (C-Ar), 130.4 (C-Ar), 123.5 (C-Ar), 122.3 (C-Ar), 121.4 (C-9), 115.5 (C-Ar), 115.2 (C-Ar), 114.8 (C-Ar), 113.9 (C-Ar), 63.3 (CH_2), 56.1, 55.6 (2 x CH_3); HRMS (ESI) required for $C_{17}H_{16}{}^{79}BrN_3O_3^+$ ($[MNa]^+$) *m/z* = 412.0273, found *m/z* = 412.0257.

4-Chloro-2-methoxy-*N*-[(1-4-methoxyphenyl)-1*H*-1,2,3 triazol-4-yl]methyl aniline (23)

Following general method D, (9) compound **23** was synthesised from propargyl analogue **17** (0.08 g, 0.4 mmol) and azide **19** (0.09 g, 0.6 mmol). Purification by flash column chromatography EtOAc/Petrol [(1:9 v/v) to EtOAc/ MeOH (one drop)] gave an orange powder (0.12 g, 84 %); M.p. 130-131°C; IR ($CHCl_3$): V_{max} = 3011, 1679, 1519, 1256 cm^{-1}; ^1H NMR (400 MHz, $CDCl_3$): δ = 7.79 (1 H, s, CH), 7.59 (2 H, d, *J* = 9.1 Hz, H-Ar), 6.99 (2 H, d, *J* = 9.1 Hz, H-Ar), 6.82 (1 H, dd, *J* = 2.2 Hz, 8.4 Hz, H-Ar), 6.75 (1 H, d, *J* = 2.2 Hz, H-Ar), 6.58 (1 H, d, *J* = 8.4 Hz, H-Ar), 4.75 (1 H, s, NH), 4.53 (2 H, s, CH_2), 3.85, 3.84 (2 x 3 H, s, 2 x CH_3); ^{13}C NMR (100 MHz, $CDCl_3$): δ = 159.8 (C-Ar), 147.5 (C-ar), 146.7 (C-Ar), 136.2 (C-Ar), 130.5 (C-Ar), 122.2 (C-Ar), 121.9 (CH), 120.8 (C-Ar), 119.9 (C-Ar), 114.8 (C-Ar), 110.6 (C-Ar), 110.3 (C-Ar), 55.7, 55.6 (2 x CH_3), 39.7 (CH_2); HRMS (EI) required for $C_{17}H_{17}{}^{35}ClN_4O_2^+$ *m/z* = 344.1040, found 344.1023.

4-Fluoro-2-methoxy-*N*-[(1-4-methoxyphenyl)-1*H*-1,2,3 triazol-4-yl]methyl aniline (24)

Following general method D, (9) compound **24** was synthesised from propargyl analogue **18** (0.14 g, 0.9 mmol) and azide **19** (0.2 g, 1.31 mmol). Purification by flash column chromatography EtOAc/Petrol [(1:9 v/v) to (1:1 v/v)] gave a cream solid (0.22 g, 77 %); M.p. 138-139 °C; IR ($CHCl_3$): V_{max} = 1609, 1519, 1287, 1036, 834 cm^{-1}; ^1H NMR (400 MHz, $CDCl_3$): δ = 7.79 (1 H, s, CH), 7.59 (2 H, d, *J* = 9.1 Hz, H-Ar), 6.99 (2 H, d, *J* = 9.1 Hz, H-Ar), 6.57 (3 H, m, H-Ar), 4.68

(1 H, s, NH), 4.52 (2 H, s, CH_2), 3.86, 3.84 (2 x 3 H, s, 2 x CH_3); ^{13}C NMR (100 MHz, $CDCl_3$): δ = 159.8 (C-Ar), 155.8 (d, *J* = 236.6 Hz, C-Ar), 147.7 (d, *J* = 9.3 Hz, C-Ar), 146.9 (C-Ar), 133.8 (C-Ar), 130.6 (C-Ar), 122.2 (CH), 120.0 (C-Ar), 114.7 (C-Ar), 110.1 (d, *J* = 9.0 Hz, C-Ar), 106.2 (*J* = 21.8 Hz, C-Ar), 98.7 (d, *J* = 27.2 Hz, C-Ar), 40.21 (CH_2), 55.7-55.6 (2 x CH_3); ^{19}F NMR (376 MHz, $CDCl_3$): -125.4 (m, 1 F); HRMS (ESI) required for $C_{17}H_{17}FN_4O_2^+$ ($[MNa]^+$) *m/z* = 351.1233, found *m/z* = 351.1222.

4-Iodo-phenyl-methanol (26)

Compound **26** was prepared following literature procedure.(16) Under a nitrogen atmosphere, 4-iodobenzoic acid (**25**) (2.00 g, 8.10 mmol, 1.0 eq.) was dissolved in anhydrous THF (16 mL). To this reaction mixture a 1 M solution of BH_3.THF (16 mL, 1.37 g, 0.016 mmol) was added slowly dropwise *via* a cannula over 15 minutes. The solution was left stirring at room temperature overnight followed by quenching by the dropwise addition of 2 M HCL (10 mL). The product was extracted with DCM (2 x 15 mL). The combined organic layers were washed with saturated $NaHCO_3$ solution (2 x 16 mL) and brine (2 x 16 mL) and then dried over $MgSO_4$ before removal of the solvent *in vacuo*. The crude product was purified by flash column chromatography EtOAc/Petrol (1:9 v/v) to yield a white powder (1.72 g, 91 %); M.p. 74-77˚C; IR ($CHCl_3$): V_{max} = 3610, 3010, 1485, 1006 cm^{-1}; ^{1}H NMR (400 MHz; $CDCl_3$): δ = 7.68 (2 H, d, *J* = 8.0 Hz, H-Ar), 7.11 (2 H, d, *J* = 8.0 Hz, H-Ar), 4.65 (2 H, d, *J* = 5.8 Hz, CH_2), 1.66 (1 H, t, *J* = 5.8 Hz, OH) which is consistent with literature values(16); ^{13}C NMR (100MHz, $CDCl_3$): δ = 140.4 (C-Ar), 137.6 (C-Ar), 128.8 (C-Ar), 93.0 (C-Ar), 64.6 (CH_2) which is consistent with literature values(16); HRMS (ESI) required for $C_7H_6IONa^+$($[M+Na]^+$) *m/z* = 256.9439, found 256.9436.

tert-Butyl-[4-iodo-benzyloxy)dimethylsilane (27)

A solution of (4-iodophenyl)methanol (**26**) (3.00 g, 12.82 mmol) in DCM (128 mL) was cooled to 0 °C followed by the addition of TBDMSCl (2.02 g, 15.38 mmol) and imidazole (1.92 g, 28.00 mmol). The solution was brought to room temperature and left stirring overnight. Saturated $NH_4Cl_{(aq)}$ solution (100 mL) was added and the phases were separated. The organic phase was washed with brine (2 x 100 mL). The combined aqueous phases were extracted with DCM (2 x 100 mL). The combined organic phases were dried over $MgSO_4$, before the solvent was removed *in vacuo*. The product was purified by flash column chromatography EtOAc/Petrol (1:10 v/v) and concentrated *in vacuo* to give white crystals (4.20 g, 12.04 mmol, 94 %); M.p. 36-38 °C; IR ($CHCl_3$): V_{max} ($CHCl_3$): 3010, 2456, 2859, 1483, 1084, 1472, 840 cm^{-1}; ^1H NMR (400 MHz, $CDCl_3$): δ = 7.65 (2 H, d, *J* = 8.5 Hz, H-Ar), 7.07 (2 H, d, *J* = 8.5 Hz, H-Ar), 4.67 (2 H, s, CH_2), 0.93 (9 H, s, 3 x CH_3), 0.09 (6 H, s, 2 x CH_3); ^{13}C NMR (100MHz, $CDCl_3$): δ = 141.1 (C-Ar), 137.2 (C-Ar), 128.0 (C-Ar); 91.9 (C-Ar), 64.3 (C-Ar), 25.9 (CH_3), 18.3 (CH_3), -5.3; HRMS (EI) required for $C_{13}H_{21}IOSi$ is 348.0406 found $[M-Bu]^+$, 290.9689; $C_{13}H_{21}IOSi$ required C,44.83; H,6.08 %, found C,44.69; H,6.04 %.

tert-Butyl-[4-(4-chloro-2-methoxy-phenoxy)-benzyloxy-dimethylsilane (28)

A literature procedure (18) was modified to synthesise compound **28**. Under a nitrogen atmosphere, the silane **27** (3 g, 8.61 mmol), 4-chloro-2-methoxyphenol (2.73 g, 17.20 mmol), Cs_2CO_3 (5.61 g, 17.22 mmol), CuI (0.082 g, 0.40 mmol), ethyl acetate (0.03 µL, 0.29 mmol), 1-naphthoic acid (2.08 g, 12.10 mmol), powdered molecular sieves (4 Å) (0.04g) and dry toluene (6 mL) were added to an oven dried sealed tube and heated to 107 °C for 3 days until almost complete consumption of the halide had occurred. The dark purple suspension was allowed to cool

to room temperature followed by the addition of DCM (10 mL). The product was filtered and DCM (2 x 20 mL) was added with stirring to remove any residue. The combined organic phases were washed with 5 % $NaOH_{(aq)}$ (100 mL). Purification by flash chromatography EtOAc/Petrol (1:20 v/v) gave a yellow oil (1.80 g, 41 %); IR ($CHCl_3$)*:*V_{max} = 3080, 1497, 1260, 838 cm^{-1}; ^{1}H NMR (400 MHz, $CDCl_3$): δ = 7.25 (2 H, d, *J* = 8.6 Hz, H-Ar), 6.97 (1 H, s, H-Ar), 6.89 (2 H, d, *J* = 8.6 Hz, H-Ar), 6.87-6.86 (2 H, m, H-Ar), 4.69 (2 H, s, CH_2), 3.82 (3 H, s, CH_3), 0.93 (9 H, s3 x CH_3), 0.09 (6 H, s, 2 x CH_3); ^{13}C NMR (100 MHz, $CDCl_3$): δ = 156.4 (C-Ar), 151.8 (C-Ar), 144.2 (C-Ar), 135.9 (C-Ar), 129.3 (C-Ar), 127.5 (C-Ar), 121.3 (C-Ar), 120.8 (C-Ar), 117.1 (C-Ar), 113.3 (C-Ar), 64.5 (CH_2), 56.1 (CH_3), 25.9 (CH_3), 25.6 (CH_2), 18.4 (CH_3); HRMS (ESI) required for $C_{20}H_{27}{}^{35}ClO_3Si^+$ [$(MNa)^+$] *m/z* = 401.1316, found 401.1090.

[4-(4-Chloro-2-methoxyphenoxy)phenyl] methanol (29)

A solution of silyl ether **28** (0.48 g, 1.30 mmol) in THF (6 mL) was cooled to 0 °C followed by the addition of TBAF (0.40 g, 1.51 mmol). The brown solution was allowed to warm to room temperature and left stirring for 4.5 hr. The solvent was removed *in vacuo*. Purification by flash chromatography EtOAc/Petrol (1:3 v/v) gave a colourless powder (0.26 g, 79 %); M.p. 65-68 °C; IR ($CHCl_3$) V_{max} = 3692, 3692, 3059, 1422, 896 cm^{-1}; ^{1}H NMR (400 MHz, $CDCl_3$): δ = 7.30 (1 H, d, J = 8.6 Hz, H-Ar), 6.91 (2 H, d, 8.6 Hz, H-Ar), 6.92 (1 H, s, H-Ar), 6.90 (2 H, m, H-Ar), 4.65 (2 H, s, CH_2), 3.83 (3 H, s, CH_3); ^{13}C NMR (100 MHz, $CDCl_3$): δ = 157.2 (C-Ar), 151.9 (C-Ar), 143.7 (C-Ar), 135.3 (C-Ar), 129.7 (C-Ar), 128.6 (C-Ar), 121.7 (C-Ar), 120.9 (C-Ar), 117.2 (C-Ar), 113.4 (C-Ar), 64.9 (CH_2), 56.2 (CH_3); HRMS (EI) required for $C_{14}H_{13}{}^{35}ClO_3$: *m/z* = 264.0548, found 264.0560.

4-Chloro-1-[4-(chloromethyl)phenoxy]-2-methoxybenzene (30)

A solution of alcohol **29** (0.14 g, 0.53 mmol) in DCM (5.3 mL) was cooled to 0 °C, followed by the addition of triethylamine (0.13 g, 1.27 mmol). The reaction mixture was left stirring for 5 minutes followed by the dropwise addition of methanesulfonylchloride (0.10 g, 0.64 mmol). The reaction mixture was allowed to warm to room temperature followed by stirring for 4 h. Saturated $NH_4Cl_{(aq)}$ solution (5 mL) was added and the phases were separated. The organic layer was washed with brine (2 x 10 mL) and the combined organic layers were extracted with DCM (2 x 10 mL) and dried over $MgSO_4$, before the solvent was removed *in vacuo*. The crude product was purified by flash chromatography EtOAc/Petrol (1:9 v/v) to give a sticky colourless solid (0.23 g, 0.74 mmol, 65 %); M.p. 83-86 °C; IR ($CHCl_3$): V_{max} = 3043, 2666, 1497, 1266, 854 cm^{-1}; ^{1}H NMR (400 MHz; $CDCl_3$): δ = 7.31 (2 H, d, *J* = 8.7 Hz, H-Ar), 6.99 (1 H, s, H-Ar), 6.92 (2 H, m, H-Ar), 6.87 (2 H, d, *J* = 8.7Hz, H-Ar), 4.57 (2 H, s, CH_2), 3.81 (3 H, s, CH_3), ^{13}C NMR (100 MHz, $CDCl_3$): δ = 157.8 (C-Ar), 154.9 (C-Ar), 152.1 (C-Ar), 143.2 (C-Ar), 131.7 (C-Ar), 130.1 (C-Ar), 122.2 (C-Ar), 121.0 (C-Ar), 116.9 (C-Ar), 113.5 (C-Ar), 56.2 (CH_3), 45.9 (CH_2); HRMS (EI) calculated for $C_{14}H_{14}{}^{35}Cl_2O_2$ m/z = 282.0214, found 282.0214.

***Di*-[(4’-chloro-2’-methoxy-)4-phenoxy] dibenzylether (31)**

A literature procedure(19) was modified to synthesise compound **31**. To a suspension of NaH (60 % dispersion in mineral oil, 0.018 g, 0.44 mmol) in DMF (1 mL), a solution of alcohol **29** (0.08 g, 0.29 mmol) in DMF (2 ml) was added, and left stirring for 30 min at room temperature. The solution was cooled to 0 °C, followed by the addition of a solution of compound **30** (0.10 g, 0.29 mmol) in DMF (2 mL). The solution was left stirring at room temperature for 4.5 h followed by the addition of water (5 mL). The solution was extracted with DCM (3 x 10 mL). The combined

organic extracts were dried over $MgSO_4$, before the solvent was removed *in vacuo*. Purification by flash column chromatography EtOAc/Petrol (1:4 v/v) gave a yellow oil (0.12 g, 79 %), IR ($CHCl_3$): V_{max} = 3009, 1491, 1271, 912, 858 cm^{-1}; ^1H NMR (400 MHz; $CDCl_3$): δ = 7.28 (4 H, d, *J* = 8.7 Hz, H-Ar), 6.98 (2 H, m, H-Ar), 6.90 (8 H, m, H-Ar), 6.89, 4.49 (4 H, s, CH_2), 3.82 (6 H, s, CH_3); ^{13}C NMR (100 MHz, $CDCl_3$): δ = 157.1 (C-Ar), 151.9 (C-Ar), 143.7 (C-Ar), 132.6 (C-Ar), 129.6 (C-Ar), 129.3 (C-Ar), 121.6 (C-Ar), 120.8 (C-Ar), 117.0 (C-Ar), 113.4 (C-Ar), 71.6 (CH_2), 56.1 (CH_3); HRMS (ESI) calculated for $C_{28}H_{24}{}^{35}Cl_2O_5N$ ($[M+NH_4])^+$: *m/z* = 528.1339, found 528.1349.

4-(4-Chloro-2-methoxyphenoxy)benzaldehyde (32)

4-Chloro-2-methoxyphenol (5.00 g, 31.50 mmol) was dissolved in anhydrous DMF (40 mL), 4-fluorobenzaldehyde (4.30 g, 34.60 mmol) and K_2CO_3 (4.79 g, 34.60 mmol) were sequentially added and the reaction mixture was heated to 130 °C and stirred for 18 h. The reaction mixture was then allowed to cool to room temperature before being diluted with H_2O (100 mL) and extracted with EtOAc (3 × 100 mL). The combined organic layers were then washed sequentially with saturated $NaHCO_3$ (aq) solution, H_2O and Brine (100 mL). The organic layer was then dried over $MgSO_4$ before the solvent was removed *in vacuo*. Purification by flash column chromatography Hexane/EtOAc (3:1) gave a dense orange oil (7.31 g, 89 %) ^1H NMR (400 MHz, $CDCl_3$) δ 9.82 (s, 1H, CHO), 7.80 – 7.64 (m, 2H, H-Ar), 6.96 – 6.93 (m, 2H, H-Ar), 6.91 – 6.86 (m, 3H, H-Ar), 3.69 (s, 3H, CH_3). ^{13}C NMR (101 MHz, $CDCl_3$) δ 190.7 (CHO), 163.2 (C-Ar), 152.4 (C-Ar), 141.6 (C-Ar), 131.9 (C-Ar), 131.4 (C-Ar), 131.2 (C-Ar), 123.3 (C-Ar), 121.2 (C-Ar), 116.2 (C-Ar), 113.7 (C-Ar), 56.1 (CH_3). HRMS (ESI) *m/z* calcd for $C_{14}H_{12}{}^{35}ClO_3$ $[M + H]^+$, 263.0469, found 263.0469

4-(4-Chloro-2-hydroxyphenoxy)benzaldehyde (33)

Compound **33** (8.00 g. 30.50 mmol) was suspended in AcOH (30 ml, 0.52 mol) followed by the addition of 47 % HBr (aq) (12 mL, 0.10 mol). The reaction mixture was then heated to 110 °C and stirred for 18 h. The reaction mixture was allowed to cool to room temperature before being concentrated *in vacuo*, the mixture was then neutralised by careful addition of $NaHCO_3$ before being diluted in H_2O (200 mL) and extracted with EtOAc (3 × 200 mL). The combined organic layers were then dried over $MgSO_4$ before the solvent was removed *in vacuo*. Purification by flash column chromatography Hexane/EtOAc (0 -> 21 %) gave a light yellow solid (2.20 g, 29 %). ^{1}H NMR (400 MHz, MeOD) δ 9.81 (s, 1H, CHO), 7.85 – 7.78 (m, 2H, H-Ar), 7.03 – 6.94 (m, 4H, H-Ar), 6.85 (dd, J = 8.6, 2.5 Hz, 1H, H-Ar). ^{13}C NMR (101 MHz, MeOD) δ 191.5 (CHO), 163.4 (C-Ar), 150.3 (C-Ar), 140.7 (C-Ar), 131.7 (C-Ar), 131.1 (C-Ar), 130.8 (C-Ar), 123.2 (C-Ar), 119.9 (C-Ar), 117.3 (C-Ar), 116.0 (C-Ar). HRMS (ESI) *m/z* calcd for $C_{13}H_8O_3{}^{35}Cl$ [M - H]$^-$, 247.0167, found 247.0175

4-(4-(4-Chloro-2-(methoxymethoxy)phenoxy)benzaldehyde (34)

Compound **33** (2.20 g, 8.90 mmol) was dissolved in CH_2Cl_2 (40 mL) under a nitrogen atmosphere. DIPEA (3.43 g, 26.54 mmol) and MOMCl (1.07 g, 13.29 mmol) were added sequentially and the reaction mixture was allowed to stir for 18 h. The reaction mixture was then diluted with saturated NH_4Cl (aq) solution (40 mL) and extracted with EtOAc (3 × 50 mL). The combined organic layers were then dried over $MgSO_4$ before the solvent was removed *in vacuo*. Purification by flash column chromatography Hexane/EtOAc (3:1) gave a light yellow oil (2.41 g, 92 %). ^{1}H NMR (400 MHz, $CDCl_3$) δ 9.77 (s, 1H, CHO), 7.75 – 7.63 (m, 2H, H-Ar), 7.17 (dd, J = 2.1, 0.5 Hz, 1H, H-

Ar), 6.94 – 6.81 (m, 4H, H-Ar), 4.98 (s, 2H, CH_2), 3.22 (s, 3H, CH_3). ^{13}C NMR (101 MHz, $CDCl_3$) δ 190.1 (CHO), 163.1 (C-Ar), 149.8 (C-Ar), 142.4 (C-Ar), 131.8 (C-Ar), 131.3 (C-Ar), 131.2 (C-Ar), 123.5 (C-Ar), 122.7 (C-Ar), 117.6 (C-Ar), 116.2 (C-Ar), 95.0 (CH_2), 56.3 (CH_3). HRMS (ESI) *m/z* calcd for $C_{15}H_{13}O_4{}^{35}ClNa$ $[M + Na]^+$, 315.0395, found 315.0398.

(4-(4-Chloro-2-(methoxymethoxy)phenoxy)phenyl)methanol (35)

Compound **34** (2.41 g, 8.25 mmol) was dissolved in MeOH (50 mL) and cooled to 0 °C. $NaBH_4$ (0.47 g, 12.38 mmol) was added portion-wise and the reaction mixture was allowed to warm to room temperature and stirred for 4 h. The reaction was quenched by the addition of $H_2$0 (40 mL) and was extracted with CH_2Cl_2 (3 × 100 mL). The combined organic layers were then dried over $MgSO_4$ before the solvent was removed *in vacuo*. Purification by flash column chromatography Hexane/EtOAc (3:1) gave a light yellow oil (2.09 g, 86 %). ^1H NMR (400 MHz, $CDCl_3$) δ 7.30 – 7.07 (m, 3H, H-Ar), 6.88 (dd, J = 8.6, 2.4 Hz, 1H, H-Ar), 6.85 – 6.80 (m, 3H, H-Ar), 5.05 (s, 2H, CH_2), 4.52 (s, 2H, CH_2OH), 3.32 (s, 3H, CH_3), 2.47 (bs, 1H, OH). ^{13}C NMR (101 MHz, $CDCl_3$) δ 157.1 (C-Ar), 149.4 (C-Ar), 144.6 (C-Ar), 135.6 (C-Ar), 129.6 (C-Ar), 128.5 (C-Ar), 122.6 (C-Ar), 122.0 (C-Ar), 117.9 (C-Ar), 117.1 (C-Ar), 95.3 (CH_2), 64.6 (CH_2OH), 56.3 (CH_3). HRMS (ESI) *m/z* calcd for $C_{15}H_5O_4{}^{35}ClNa$ $[M + Na]^+$, 317.0551, found 317.0558

4-Chloro-1-(4-(chloromethyl)phenoxy)-2-(methoxymethoxy)benzene (36)

Compound **35** (0.35 g, 1.17 mmol) and Et_3N (0.24 g, 2.34 mmol) were dissolved in CH_2Cl_2 (7 mL) and cooled to 0 °C. Methanesulfonyl chloride (0.20 g, 1.75 mmol) was added dropwise and the reaction mixture was allowed to warm to room temperature and stirred for 4 h. The reaction

mixture was diluted with saturated NH_4Cl (aq) solution (10 mL) and extracted with CH_2Cl_2 (3 × 10 mL). The combined organic layers were then dried over $MgSO_4$ before the solvent was removed *in vacuo*. Purification by flash column chromatography Hexane/EtOAc (4:1) gave a light yellow oil (0.20 g, 56 %). ^{1}H NMR (400 MHz, $CDCl_3$) δ 7.37 – 7.31 (m, 2H, H-Ar), 7.30 – 7.26 (m, 1H, H-Ar), 7.03 – 6.95 (m, 2H, H-Ar), 6.94 – 6.89 (m, 2H, H-Ar), 5.16 (s, 2H, CH_2), 4.59 (s, 2H, CH_2Cl), 3.42 (s, 3H, CH_3). ^{13}C NMR (101 MHz, $CDCl_3$) δ 158.0 (C-Ar), 149.6 (C-Ar), 144.0 (C-Ar), 131.8 (C-Ar), 130.1 (C-Ar), 122.6 (C-Ar), 122.6 (C-Ar)-, 117.8 (C-Ar), 117.0 (C-Ar), 95.3 (CH_2), 56.4 (45CH_3), 45.9 (CH_2Cl). HRMS (ESI) *m/z* calcd for $C_{15}H_{14}O_3{}^{35}Cl_2Na$ [M + Na]$^+$, 335.0212, found 335.0213

4,4’-(((Oxy*bis*(methylene))*bis*(4,1-phenylene))*bis*(oxy))*bis*(1-chloro-3-(methoxymethoxy)benzene) (37)

Compound **36** (0.13 g, 0.43 mmol) was dissolved in anhydrous DMF (5 mL) and cooled to 0 °C. NaH (60 % dispersion in mineral oil, 33 mg, 0.83 mmol) was added in a single portion and the reaction mixture was allowed to stir for 1 h. Compound **35** (0.20 g, 0.65 mmol) in DMF (2 mL) was then added dropwise and the reaction mixture was allowed to warm to room temperature before being stirred for a further 18 h. The reaction mixture was quenched by the addition of H_2O (5 mL) before being extracted with EtOAc (3 × 10 mL). The combined organic layers were then washed sequentially with saturated $NaHCO_3$ (aq) solution, H_2O and Brine (20 mL). The organic layer was then dried over $MgSO_4$ before the solvent was removed *in vacuo*. Purification by flash column chromatography Hexane/EtOAc (3:1) gave a light yellow oil (0.11 g, 45 %). ^{1}H NMR (400 MHz, $CDCl_3$) δ 7.33 – 7.30 (m, 4H, H-Ar), 7.29 – 7.27 (m, 2H, H-Ar), 7.01 – 6.97 (m, 2H, H-Ar), 6.96 – 6.91 (m, 6H, H-Ar), 5.17 (s, 4H, OCH_2O), 4.52 (s, 4H, $ArCH_2$), 3.44 (s, 6H, CH_3). ^{13}C

NMR (101 MHz, $CDCl_3$) δ 157.3 (C-Ar), 149.4 (C-Ar), 144.7 (C-Ar), 132.7 (C-Ar), 129.6 (C-Ar), 129.4 (C-Ar), 122.6 (C-Ar), 122.1 (C-Ar), 117.9 (C-Ar), 117.1 (C-Ar), 95.4 (OCH_2O), 71.6 ($ArCH_2$), 56.4 (CH_3). HRMS (ESI) *m/z* calcd for $C_{30}H_{28}O_7{}^{35}Cl_2Na$ $[M + Na]^+$, 593.1104, found 593.1088

_Di_-[(4’-chloro-2’-hydroxy-)4-phenoxy] dibenzylether (38)

Compound **37** (0.11 g, 0.19 mmol) was dissolved in MeOH (7 mL) followed by the addition of 6 M HCl (0.2 mL, 1.2 mmol). The reaction mixture was heated to reflux and stirred for 2 h. The reaction mixture was then allowed to cool to room temperature before being concentrated *in vacuo*, the reaction mixture was then diluted with saturated $NaHCO_3$ (aq) solution (10 mL) and extracted with EtOAc (3 × 15 mL). The combined organic layers were then dried over $MgSO_4$ before the solvent was removed *in vacuo*. Purification by reverse-phase high performance liquid chromatography (Method B) gave an off-white solid (0.04 g, 44 %). ^1H NMR (400 MHz, $CDCl_3$) δ 7.40 – 7.33 (m, 4H, H-Ar), 7.07 (d, J = 2.2 Hz, 2H, H-Ar), 7.04 – 6.98 (m, 4H, H-Ar), 6.86 – 6.78 (m, 4H, H-Ar), 5.80 (s, 2H, OH), 4.56 (s, 4H, CH_2). ^{13}C NMR (101 MHz, $CDCl_3$) δ 156.1 (C-Ar), 148.1 (C-Ar), 142.4 (C-Ar), 133.8 (C-Ar), 129.7 (C-Ar), 129.5 (C-Ar), 120.6 (C-Ar), 119.5 (C-Ar), 118.0 (C-Ar), 116.7 (C-Ar), 71.6 (CH_2). HPLC r.t. ~ 17 min (Method B) HRMS (ESI) *m/z* calcd for $C_{26}H_{20}O_5{}^{35}Cl_2Na$ $[M + Na]^+$, 505.0580, found 505.0594. LC-MS Purity = 92 %

4-Chloro-2-methoxy-1-(4-[4-methoxybenzyl] oxy] methyl phenoxy) benzene (39)

A literature procedure (19) was modified to synthesise compound **39**. To a suspension of NaH (60 % dispersion in mineral oil, 0.02 g, 0.4 mmol) in DMF (1 mL), a solution of (4-methoxyphenyl)methanol (0.04 g, 0.3 mmol) in DMF (2 ml) was added and left stirring for 30 min

at room temperature. The solution was cooled to 0 °C, followed by the addition of a solution of compound **31** (0.10 g, 0.3 mmol) in DMF (2 mL). The solution was left stirring at room temperature for 4.5 h followed by the addition of water (5 mL). The reaction mixture was extracted with DCM (3 x 10 mL). The combined organic extracts were dried over $MgSO_4$ and the solvent was removed *in vacuo*. The crude product was purified by flash chromatography EtOAc/Petrol (1:19 v/v) to give a colourless oil (0.08 g, 67 %), IR ($CHCl_3$): V_{max} = 3011, 1612-1597, 1464, 1251, 1118, 1032, 844 cm^{-1}; ^1H NMR (400 MHz, $CDCl_3$): δ = 7.30 (4 H, d, *J* = 8.6 Hz, H-9, H-14), 7.00 (1 H, m, H-Ar), 6.92 (6 H, m, H-Ar), 4.51, 4.49 (2 x2 H, s, 2 x CH_2), 3.84, 3.83 (s, 2 x3 H, 2 x CH_3); ^{13}C NMR (100 MHz, $CDCl_3$): δ = 159.2 (C-Ar), 157.1 (C-Ar), 151.9 (C-Ar), 143.9 (C-Ar), 132.8 (C-Ar), 130.3 (C-Ar), 129.6 (C-Ar), 129.4 (C-Ar), 121.6 (C-Ar), 120.9 (C-Ar), 117.1 (C-Ar), 113.8 (C-Ar), 113.4 (C-Ar), 71.78, 71.35 (C-Ar), 56.2 (2 x CH_2), 55.3 (2 x CH3); HRMS (ESI) required for $C_{22}H_{21}{}^{35}ClO_4$ ($[M+Na]^+$): *m/z* = 407.1026, found 407.1024.

***Di*-1,1'(4-Iodo-phenyl)-dimethylether (40)**

A literature procedure was modified to synthesise compound **40** (17). Under a nitrogen atmosphere, to a suspension of NaH (60 % dispersion in mineral oil, 0.31 g, 17.67 mmol) in anhydrous THF (13 mL), a solution of (4-iodophenyl)methanol (0.20 g, 0.86 mmol) in anhydrous THF (16 mL) was added at 0 °C and stirred for 30 min at room temperature. A solution of 4-iodo-benzylbromide (0.50 g, 1.71 mmol) in dry THF (4 mL) was added to the solution at 0 °C. The solution was heated to reflux overnight and cooled to room temperature, followed by dropwise addition of water (13 mL), and extraction with EtOAc (3 x 25 mL). The combined organic layers were dried over $MgSO_{4,}$ before the solvent was removed *in vacuo*. The crude product was purified by flash column chromatography EtOAc/Petrol (1:9 v/v) to give a white powder (0.28 g, 71 %);

M.p. 103 – 105 ˚C; IR ($CHCl_3$): V_{max} = 2860, 1083, 1488 cm^{-1}; ^1H NMR (400 MHz; $CDCl_3$): δ = 7.68 (4 H, m, H-Ar), 7.09 (4 H, m, H-Ar), 4.48 (4 H, s, CH_2); ^{13}C NMR (100 MHz, $CDCl_3$): δ = 137.7 (C-Ar), 137.5 (C-Ar), 129.5 (C-Ar), 93.2 (C-Ar), 71.5 (CH_2); HRMS (EI) required for $C_{14}H_{12}I_2O^+$ is 449.8970. No parent ion was observed found $[M\text{-}C_7H_7IO]^+$, 217.9595 and $[M–C_7H_6I]^+$, 232.9468

4-(Chloromethyl)benzylmethanesulfonate (42)

To a solution of (4-methoxyphenyl)methanol (5.00 g, 40.3 mmol) in DCM (400 mL) at 0 °C, triethylamine (19.53 g, 193.00 mmol) was added, and the solution was left stirring for 5 min, followed by the addition of methanesulfonyl chloride (18.5 g, 161.00 mmol). The solution was allowed to warm to room temperature and stirred for 4 h, followed by the addition of saturated $NH_4Cl_{(aq)}$ solution (400 mL). The phases were separated and the organic phase was washed with brine (3 x 200 mL). The combined aqueous phases were extracted with DCM (3 x 200 mL). The combined organic phases were dried over $MgSO_4$, before the solvent was removed *in vacuo*. Purification by flash column chromatography EtOAc/Petrol (1/9 v/v) gave yellow crystals (4.92 g, 44 %); M.p. 40-42 °C; IR ($CHCl_3$): V_{max} = 1505, 1375, 1150, 872 cm^{-1}; ^1H NMR (400 MHz, $CDCl_3$): δ = 7.47 (2 H, d, *J* = 8.7 Hz, H-Ar), 7.30 (2 H, d, *J* = 8.7 Hz, H-Ar); 4.61 (2 H, s, H-Ar), 3.18 (3 H, s, CH_3); ^{13}C NMR (100 MHz, $CDCl_3$): δ = 148.9 (C-Ar), 130.3 (C-Ar), 122.3 (C-Ar), 45.1 (CH_3), 37.5 (CH_2); HRMS (EI) required for $C_8H_9{}^{35}ClO_3S$ is 219.9961 found 219.9955.

4-[(2-Acetyl-4-chlorophenoxy)methyl]phenylmethanesulfonate (43)

Following general procedure B,(12) compound **43** was synthesised from ketone **41** (0.30 g, 1.80 mmol) and mesylate **42** (0.43 g, 2.10 mmol). Purification by flash column chromatography

EtOAc/Petrol (1:9 to 3:7 v/v) gave cream flakes (0.28 g, 51 %); M.p. 97-98°C, IR ($CHCl_3$): V_{max} = 1679, 1375, 1151, 873 cm^{-1}; ^1H NMR (400 MHz, $CDCl_3$): δ = 7.71 (1 H, d, *J* = 2.8 Hz, H-Ar), 7.48 (2 H, d, *J* = 8.7 Hz, H-Ar), 7.39 (1 H, dd, *J* = 2.8 Hz, 8.9 Hz, H-Ar), 7.33 (2 H, d, *J* = 8.7 Hz, H-Ar), 6.94 (1 H, d, *J* = 8.9 Hz, H-Ar), 5.15 (2 H, s, CH_2), 2.98 (3 H, s, CH_3), 2.58 (3 H, s, CH_3); ^{13}C NMR (100 MHz, $CDCl_3$): δ = 198.2 (C-14), 156.1 (C-Ar), 148.9 (C-Ar), 135.1 (C-Ar), 133.1 (C-Ar), 130.3 (C-Ar), 129.9 (C-Ar), 129.1 (C-Ar), 126.7 (C-Ar), 122.5 (C-Ar), 114.3 (C-Ar), 70.2 (CH_2), 37.6 (CH_3), 31.9 (CH_3). HRMS (micOTOF) required for $C_{16}H_{15}{}^{35}ClO_5S^+$ ($[MNa]^+$) *m/z* = 377.0226, found *m/z* =377.0222.

4-[(2-Acetyl-4-chlorophenoxy) methyl] phenylmethanesulfonate (44)

A literature procedure (20) was adapted to synthesise compound **44**. To a solution of ketone **43** (0.28 g, 1.01 mmol) in chloroform (2 mL) *m*-CPBA (70-75 % purity) (0.87 g, 5.10 mmol), was added. The white suspension was left stirring at room temperature overnight. The reaction was quenched by the addition of saturated $NaS_2SO_{4(aq)}$ solution (5 mL). The aqueous layer was extracted with DCM (3 x 10mL). The combined organic layers were dried over $MgSO_4$, the solvent was removed *in vacuo*. Purification by flash column chromatography EtOAc/Petrol (1:9 to 3:7 v/v) gave a white solid (0.25 g, 68 %); M.p. 132-133 °C; V_{max}: ($CHCl_3$): 1764, 1498, 1373, 1150, 873 cm^{-1}; ^1H NMR (400 MHz, $CDCl_3$): δ = 7.42 (2 H, d, *J* = 8.7 Hz, H-Ar), 7.30 (2 H, d, *J* = 8.7 Hz, H-Ar), 7.14 (1 H, dd, *J* = 2.5 Hz, 8.7 Hz, H-Ar), 7.09 (1 H, d, *J* = 2.5 Hz, H-Ar), 6.89 (1 H, d, *J* = 8.7 Hz, H-Ar), 5.07 (2 H, s, CH_2), 3.16 (3 H, s, CH_3), 2.28 (3 H, s, CH_3); ^{13}C NMR (100 MHz, $CDCl_3$): δ = 148.8 (C-13), 140.6 (C-Ar), 135.7 (C-Ar), 128.7 (C-Ar), 126.7 (C-Ar), 126.0 (C-Ar), 123.5 (C-Ar), 122.3 (C-Ar), 114.7 (C-Ar), 70.1 (CH_2), 37.5 (CH_3), 20.6 (CH_3);

HRMS (ESI) required for $C_{16}H_{15}{}^{35}ClO_6S^+$ ($[MNa]^+$) *m/z* = 393.0176, found *m/z* = 393.0164; $C_{16}H_{15}ClO_6S$ requires C, 51.83; H, 4.08 % found C, 51.91; H, 4.15 %.

2-*trans*-Octenyl CoA

Using an method adapted from the literature(21), The reaction vessel was covered with foil. The addition of the starting materials was done in the dark: potassium carbonate (35 mg, 63.6 µmol) was dissolved in water (2.5 mL), followed by the addition of coenzyme A (50 mg, 63.6 µmol) and 2-*trans*-octenoic acid (Alfa Aesar, 94%) (16 µl, 110 µmol). THF (2.5 mL) was added followed by the addition of PyBOP (0.053 g, 102 µmol). The solution was left stirring at room temperature for 5 h and completion of the reaction was monitored with DTNB (to determine free CoA thiol). The organic layer was removed by evaporation and the water was removed by lyophilisation to give a crude cream solid. The crude product was dissolved in distilled water and purified by HPLC using 20 mM ammonium acetate (pH 5.8) (A) and acetonitrile (B). Gradient elution over 25 minutes 100 % A (0-1 min), 0-10 % B (1.01-2min), 10 % B (1.01-2 min), 10-20 % B (2-2.01 min), 20 % (2.01-5 min), 20-25 % B (5-5.01 min) 25-30 % B (5.01-15 min), 30-95 % B (15.01 min), 95 % (15.01-17 min),95-0 % B (17-20 min), 100 % A (20-25 min) at a flow rate of 2 mL/min gave an eluted product which was lyophilised thrice to give a white powder (27.7 mg, 24.3 µmol, 38 %); V_{max} (KBr): 3412, 2359, 2927, 1652, 1239, 1078, 951 cm^{-1}; ^{1}H NMR (400 MHz, $CDCl_3$): δ = 8.43 (1 H, s, H-Ar), 8.14 (1 H, s, H-Ar), 6.84 (1 H, m, CH), 6.04 (2 H, m, 2 x CH), 4.7 (2 H, m, 2 x CH), 4.47 (1 H, s, CH), 4.14 (bs, 2 H, CH_2), 3.91 (s, 1 H, CH), 3.72 (1 H, m, CH_B), 3.46 (1 H, m, CH_A), 3.32 (2 H, m, CH_2), 3.24 (2 H, m, CH_2), 2.92 (2 H, m, CH_2), 2.3 (2 H, m, CH_2), 2.06 (2 H, m, CH_2), 1.29 (2 H, m, CH_2), 1.13 (4 H, m, 2 x CH_2), 0.78 (3 H, s, CH_3), 0.73 (3 H, m, CH_3), 0.65 (3 H, s, CH_3); ^{31}P-NMR (162 MHz, D_2O): -0.6, -11.3 (2 P); ^{13}C NMR (100 MHz, D_2O): δ = 193.7, 174.7,

173.91, 152.3, 148.9, 148.2, 141.2, 127.6, 118.5, 86.8, 83.4, 74.0, 73.9, 71.8, 65.1, 38.6, 38.3, 38.2, 35.4, 35.3, 31.6, 30.5, 30.4, 27.7, 26.7, 21.7, 20.8, 18.1, 13.2; HRMS (ESI) required for $C_{29}H_{47}N_7O_{17}P_3S^-$: *m/z* = 890.2040, found 890.1869.

NMR Data

(see below)

4-chloro-22-methoxy-1-[(4-methoxybenzyl)oxy]benzene

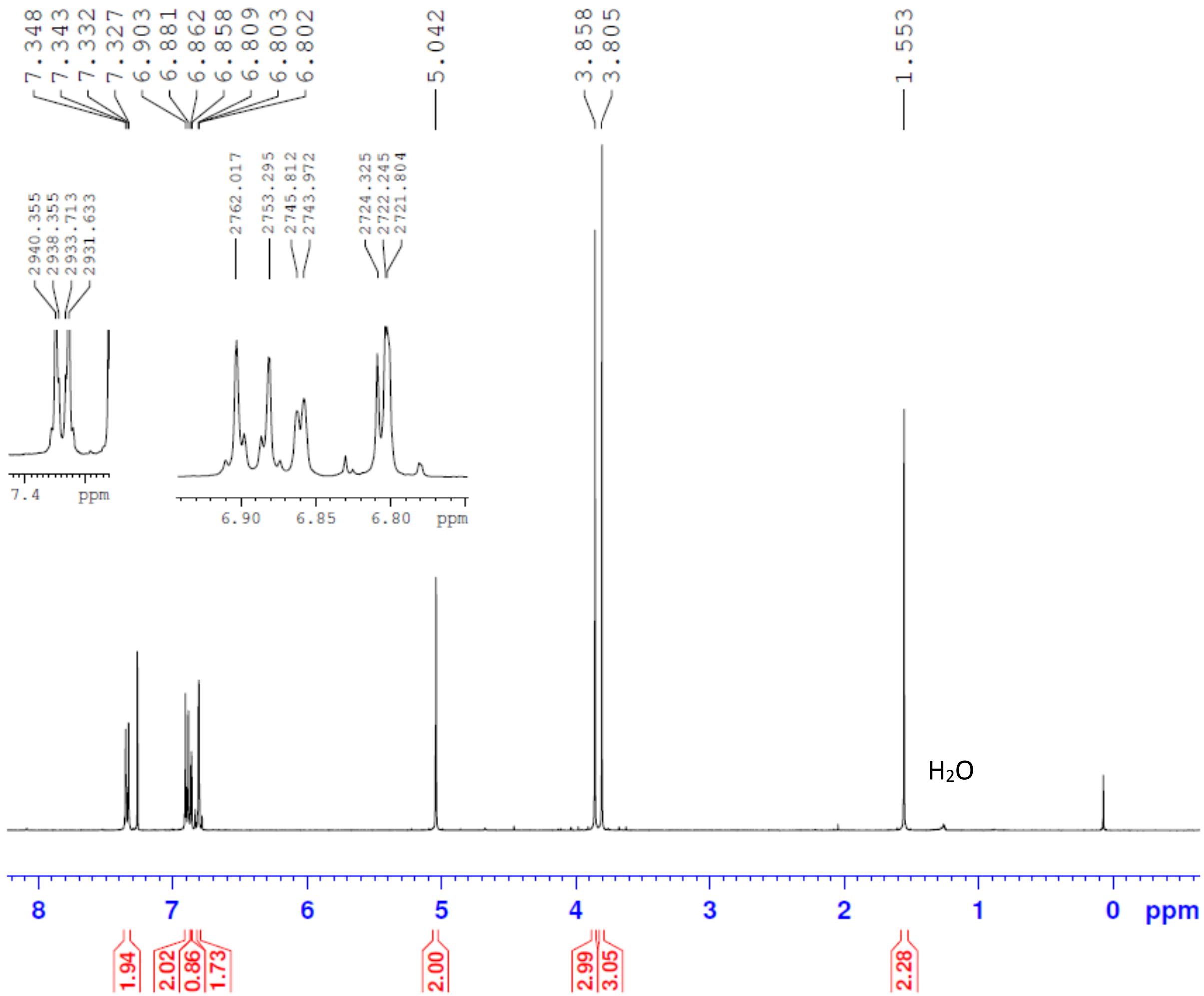

NAME	sc128purefiltration
EXPNO	1
PROCNO	1
Date_	20101103
Time	17.20
INSTRUM	spect
PROBHD	5 mm QNP 1H/13
PULPROG	zg30
TD	65536
SOLVENT	CDCl3
NS	16
DS	2
SWH	8278.146 Hz
FIDRES	0.126314 Hz
AQ	3.9584243 sec
RG	2896.3
DW	60.400 usec
DE	6.50 usec
TE	298.2 K
D1	1.00000000 sec
TD0	1

======== CHANNEL f1 ========

NUC1	1H
P1	11.10 usec
PL1	-1.10 dB
SFO1	400.1324710 MHz
SI	32768
SF	400.1300095 MHz
WDW	EM
SSB	0
LB	0.30 Hz
GB	0
PC	1.00

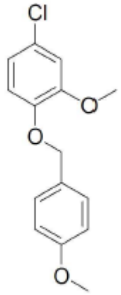

1

4-chloro-22-methoxy-1-[(4-methoxybenzyl)oxy]benzene

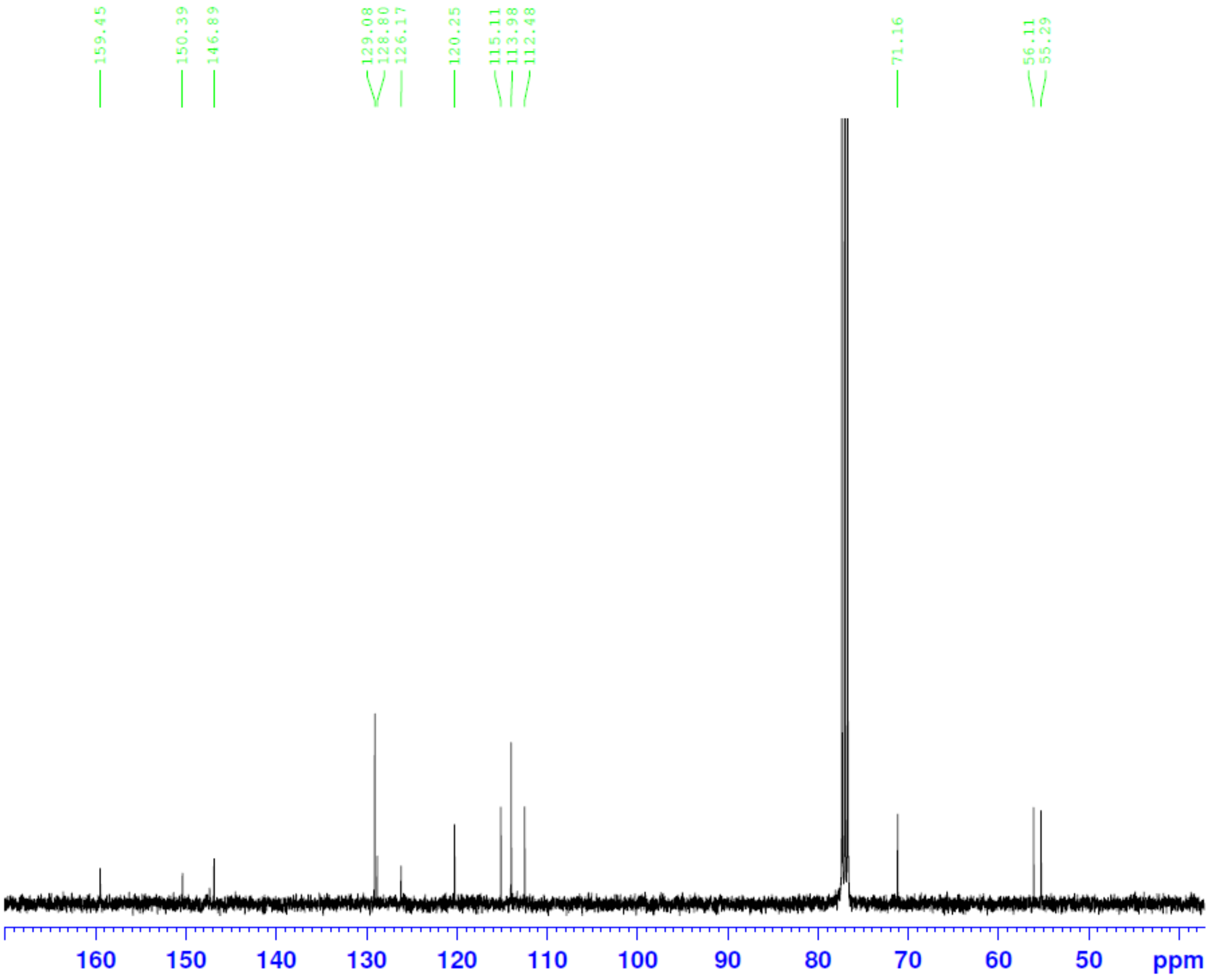

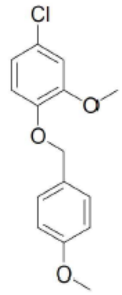

1

4-fluoro-2-methoxy-1-[(4-methoxybenzyl)oxy]benzene

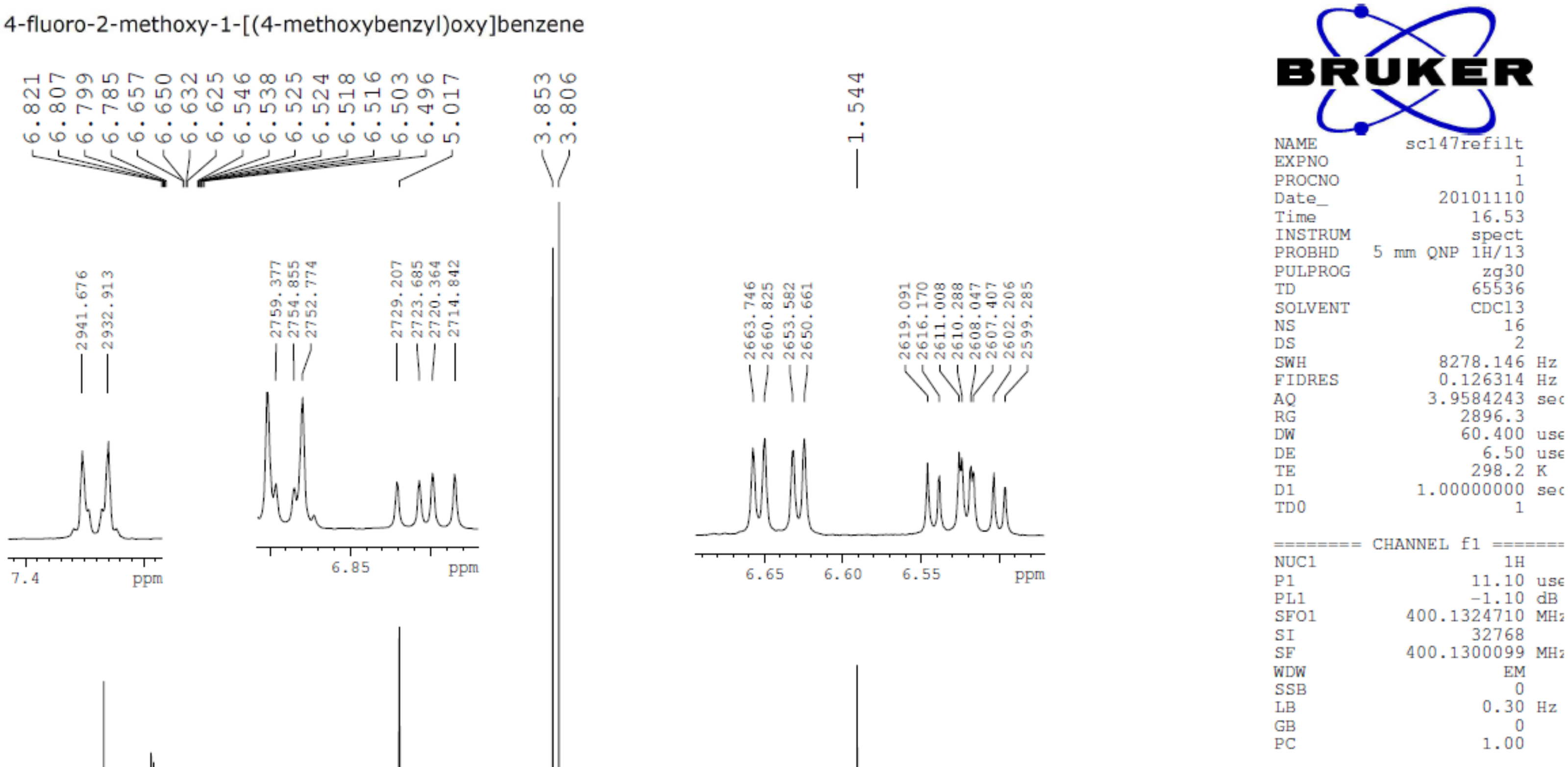

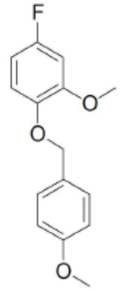

2

UserID s_che SampleID 147pure SupervisorID thoma Lab Phone No. 67990 S
F-NMR

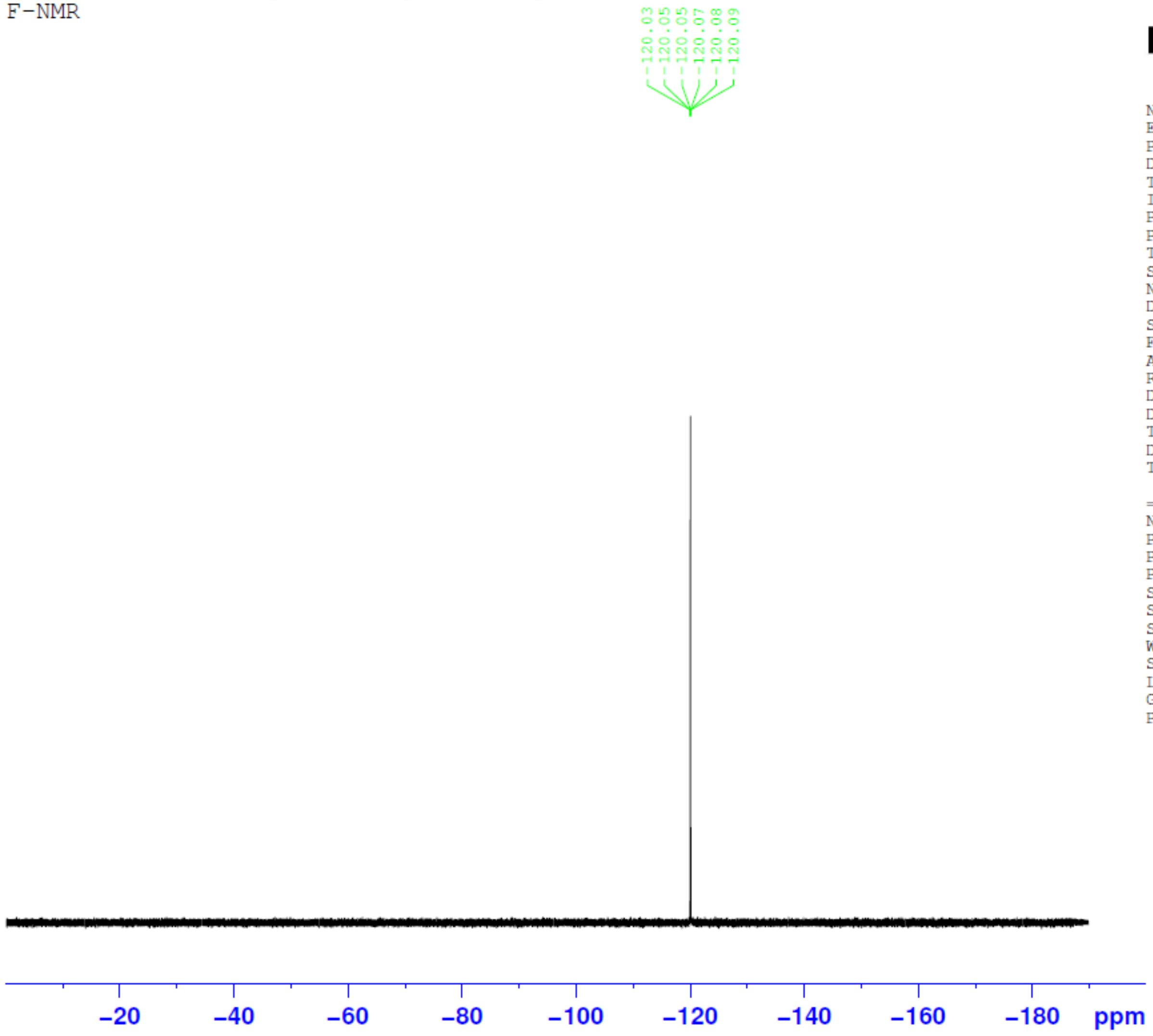

NAME	s_che.147pure
EXPNO	1
PROCNO	1
Date_	20101114
Time	12.22
INSTRUM	av400
PROBHD	5 mm PABBO BB-
PULPROG	zg
TD	262144
SOLVENT	CDCl3
NS	64
DS	2
SWH	75187.969 Hz
FIDRES	0.286819 Hz
AQ	1.7433076 sec
RG	2048
DW	6.650 usec
DE	6.50 usec
TE	298.2 K
D1	2.00000000 sec
TD0	1

======== CHANNEL f1 ========

NUC1	19F
P1	10.00 usec
PL1	3.00 dB
PL1W	4.67061329 W
SFO1	376.4644798 MHz
SI	262144
SF	376.4983670 MHz
WDW	EM
SSB	0
LB	0.80 Hz
GB	0
PC	1.00

4-bromo-2-methoxy-1-[(4-methoxybenzyl)oxy]benzene

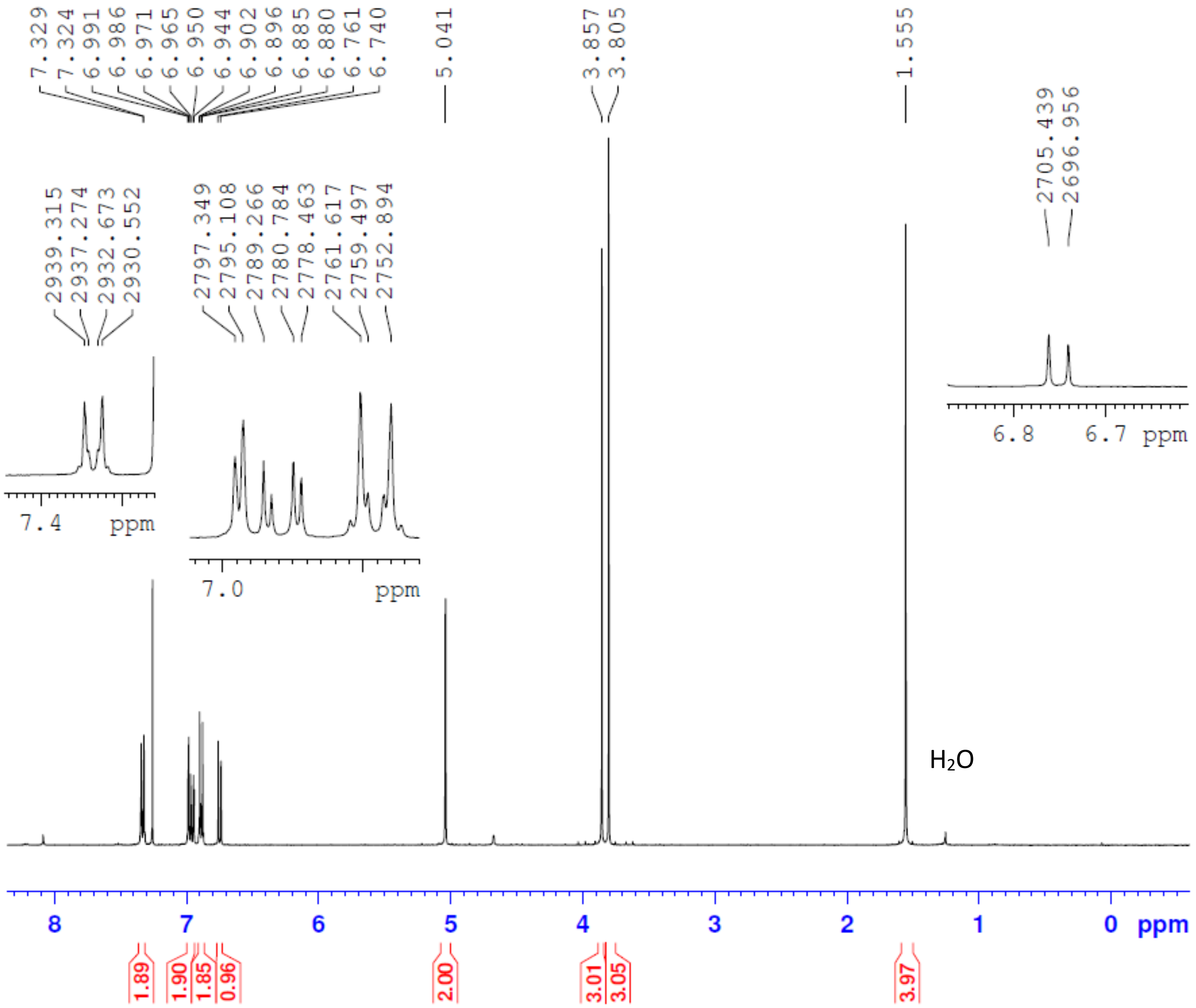

BRUKER

NAME	SC148FILT_PURE	
EXPNO	1	
PROCNO	1	
Date_	20110113	
Time	15.49	
INSTRUM	spect	
PROBHD	5 mm QNP 1H/13	
PULPROG	zg30	
TD	65536	
SOLVENT	CDCl3	
NS	16	
DS	2	
SWH	8278.146	Hz
FIDRES	0.126314	Hz
AQ	3.9584243	sec
RG	4096	
DW	60.400	usec
DE	6.50	usec
TE	298.3	K
D1	1.00000000	sec
TD0	1	

======== CHANNEL f1 ========

NUC1	1H	
P1	11.10	usec
PL1	-1.10	dB
SFO1	400.1324710	MHz
SI	32768	
SF	400.1300095	MHz
WDW	EM	
SSB	0	
LB	0.30	Hz
GB	0	
PC	1.00	

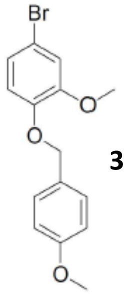

3

4-bromo-2-methoxy-1-[(4-methoxybenzyl)oxy]benzene

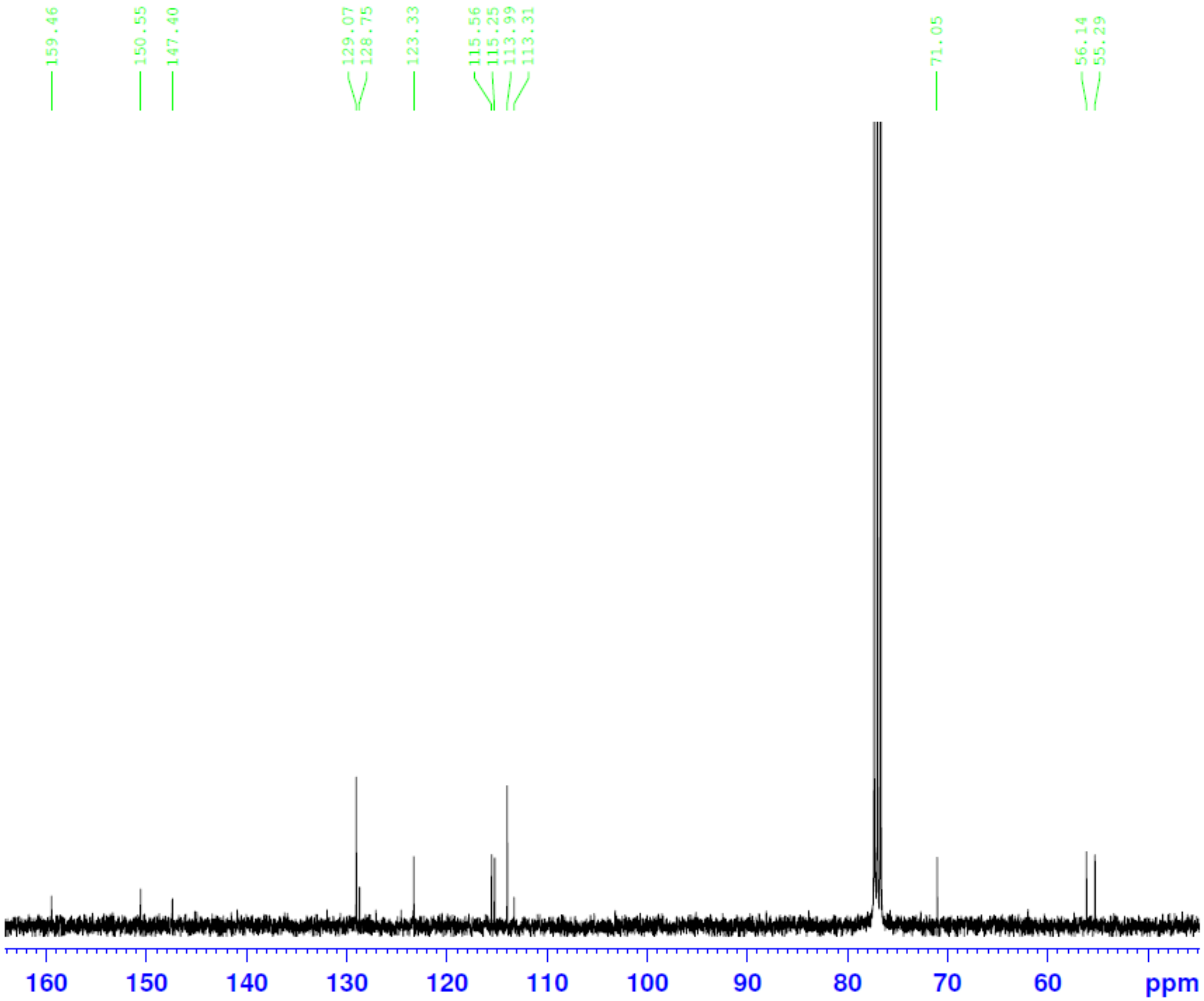

BRUKER

```
NAME         SC148FILT_PURE
EXPNO                     2
PROCNO                    1
Date_              20110114
Time                   8.14
INSTRUM               spect
PROBHD   5 mm QNP 1H/13
PULPROG             zgpg30
TD                   65536
SOLVENT               CDCl3
NS                    1024
DS                       4
SWH           23980.814 Hz
FIDRES         0.365918 Hz
AQ            1.3664756 sec
RG                   16384
DW               20.850 usec
DE                 6.50 usec
TE                298.3 K
D1           2.00000000 sec
D11          0.03000000 sec
TD0                      1

======== CHANNEL f1 ========
NUC1                   13C
P1                 9.38 usec
PL1                0.00 dB
SFO1        100.6228298 MHz

======== CHANNEL f2 ========
CPDPRG2            waltz16
NUC2                    1H
PCPD2             80.00 usec
PL2               -1.10 dB
PL12              16.06 dB
PL13              21.00 dB
SFO2        400.1316005 MHz
SI                   32768
SF          100.6127690 MHz
WDW                     EM
SSB                      0
LB                 1.00 Hz
GB                       0
PC                    1.40
```

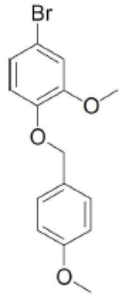

3

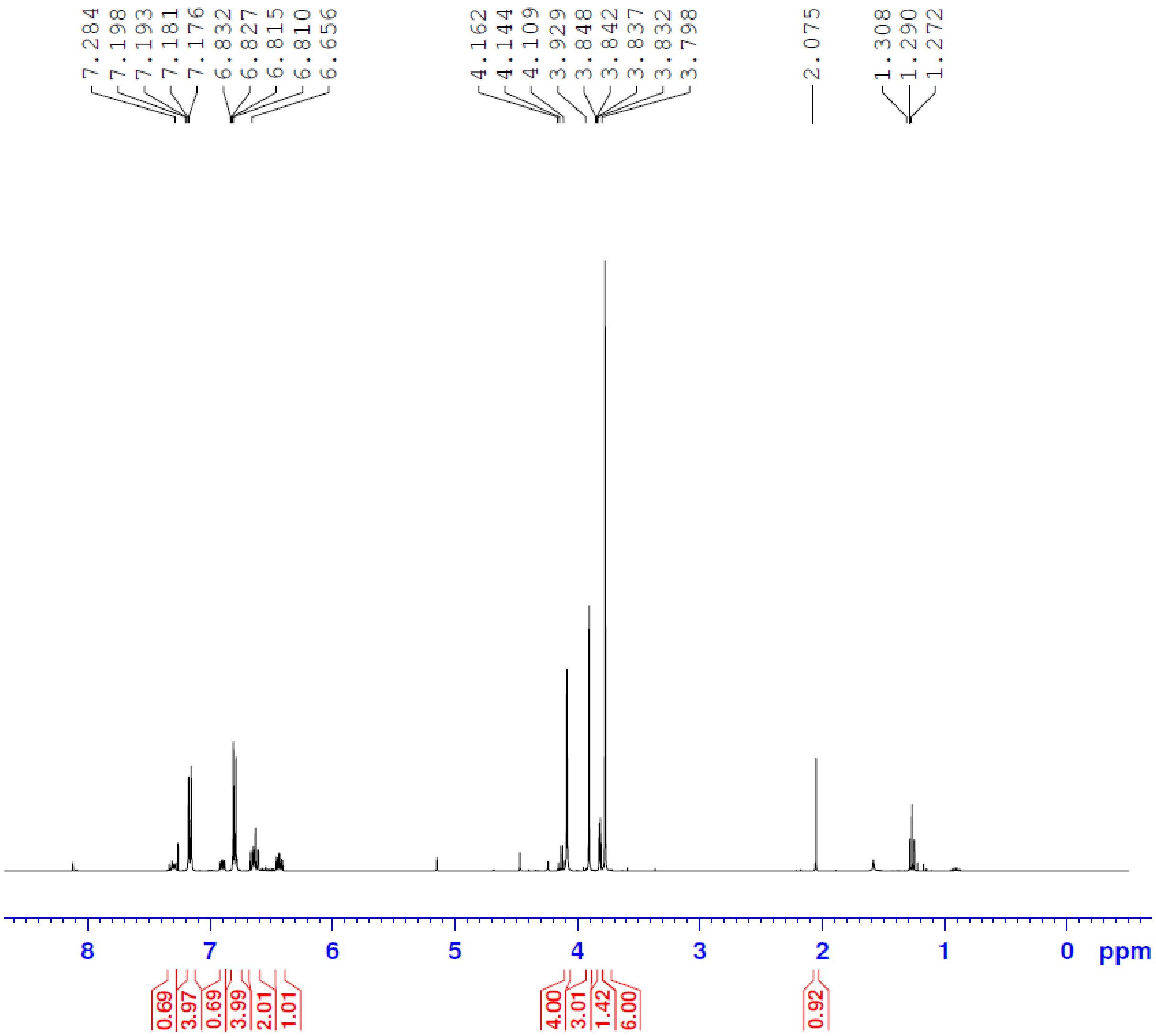


```
NAME      s_che.260spot2recolum
EXPNO                 1
PROCNO                1
Date_          20110304
Time              12.13
INSTRUM           av400
PROBHD   5 mm PABBO BB-
PULPROG            zg30
TD                32768
SOLVENT           CDCl3
NS                   16
DS                    2
SWH            4789.272 Hz
FIDRES         0.146157 Hz
AQ            3.4210291 sec
RG                 90.5
DW              104.400 usec
DE                 6.00 usec
TE                298.2 K
D1           1.00000000 sec
TD0                   1

======== CHANNEL f1 ========
NUC1                 1H
P1                11.75 usec
PL1               -2.00 dB
PL1W        16.00390816 W
SFO1        400.1322007 MHz
SI                32768
SF          400.1300099 MHz
WDW                  EM
SSB                   0
LB                 0.30 Hz
GB                    0
PC                 1.00
```

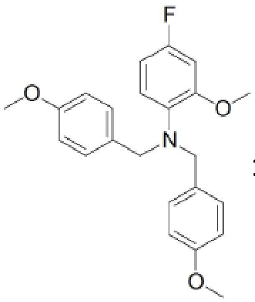

13

4-fluoro-2-methoxy-N-(4-bismethoxybenzyl) aniline

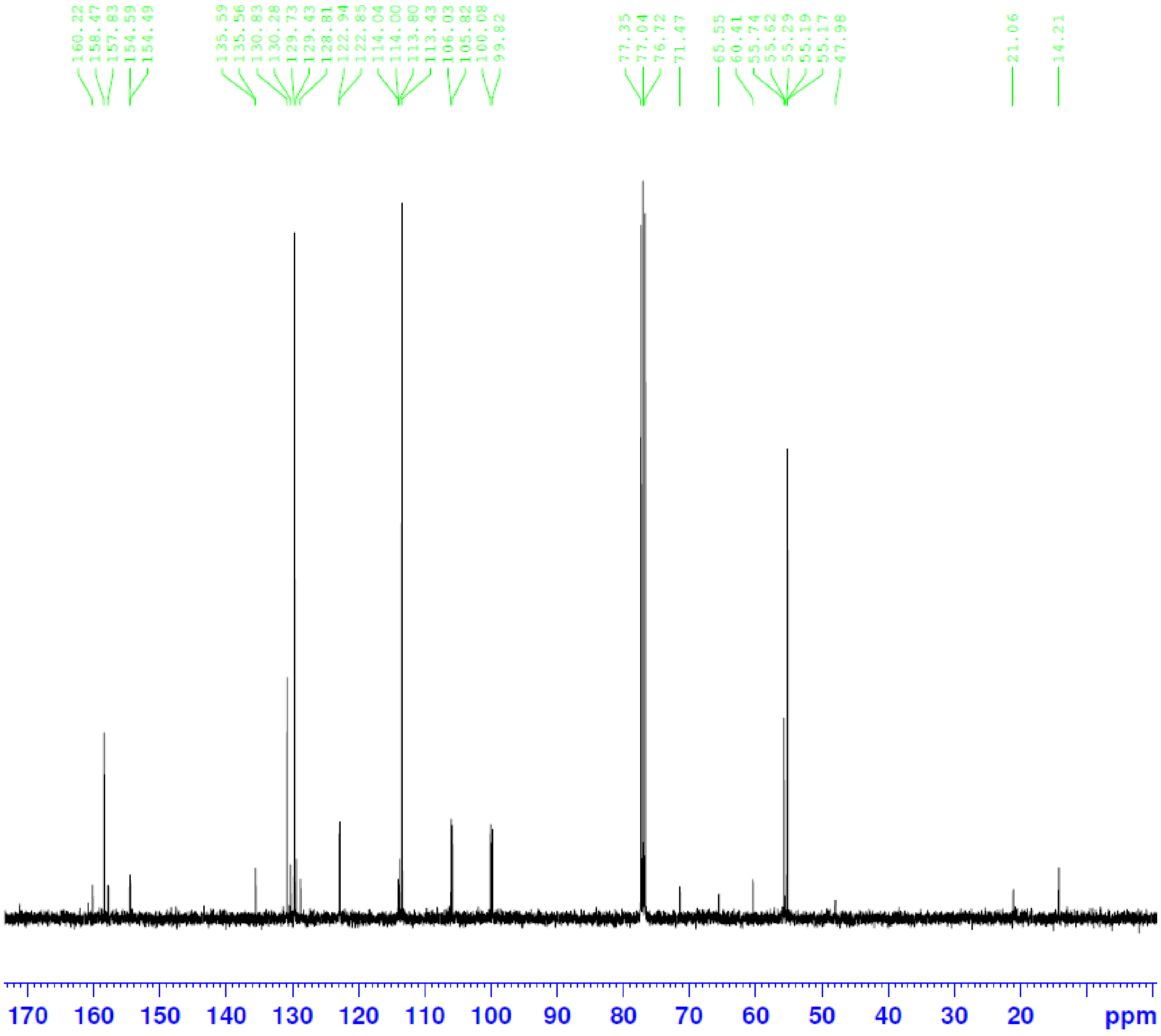

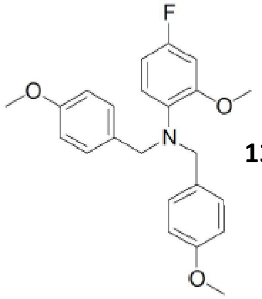

13

4-fluoro-2-methoxy-N-(4-bismethoxybenzyl) aniline

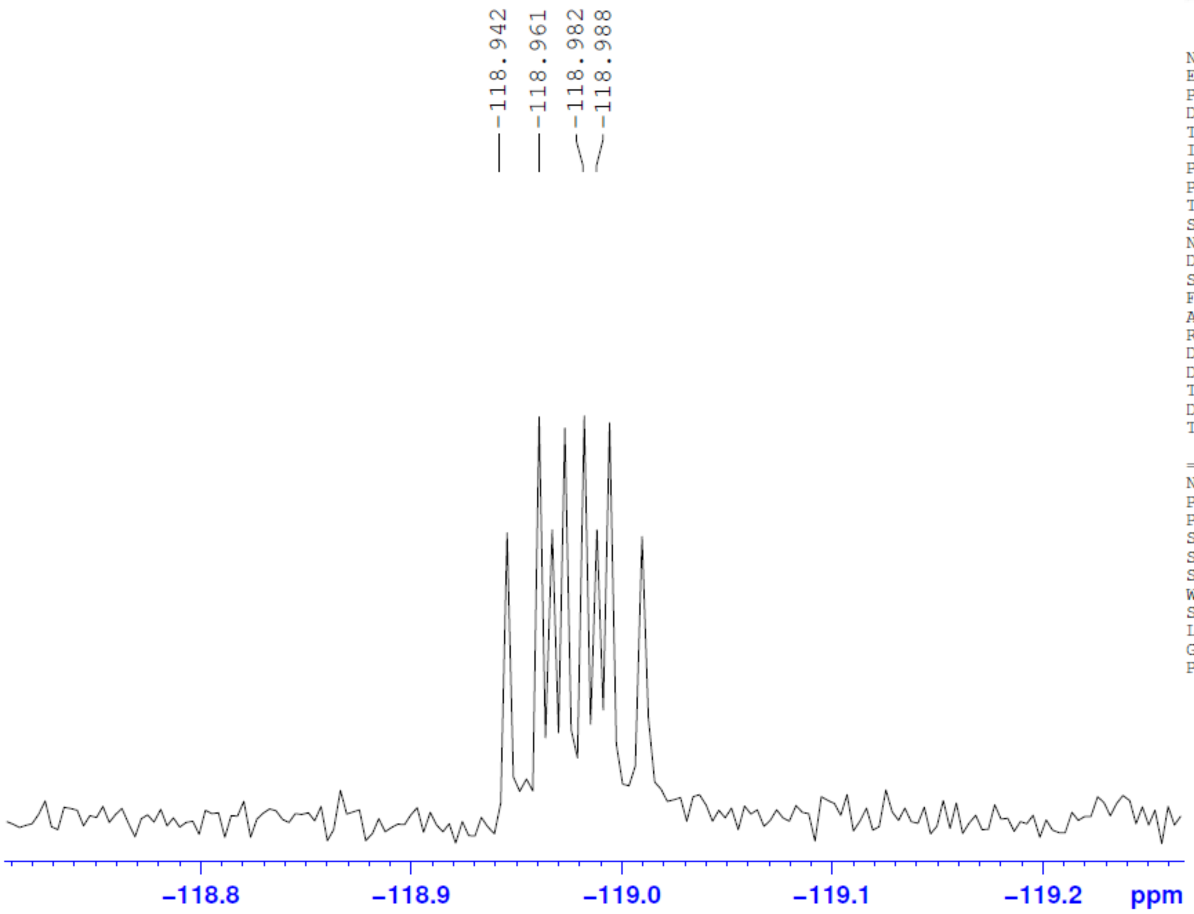

NAME	sc260p_Wup
EXPNO	7
PROCNO	1
Date_	20110211
Time	6.00
INSTRUM	spect
PROBHD	5 mm QNP 1H/13
PULPROG	zgflqn
TD	131072
SOLVENT	CDCl3
NS	16
DS	4
SWH	75187.969 Hz
FIDRES	0.573639 Hz
AQ	0.8716788 sec
RG	32768
DW	6.650 usec
DE	6.50 usec
TE	298.2 K
D1	1.00000000 sec
TD0	1
======== CHANNEL f1 ========	
NUC1	19F
P1	13.00 usec
PL1	0.00 dB
SFO1	376.4607164 MHz
SI	65536
SF	376.4983660 MHz
WDW	no
SSB	0
LB	0.00 Hz
GB	0
PC	1.00

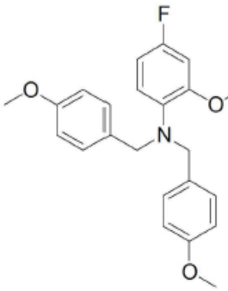

13

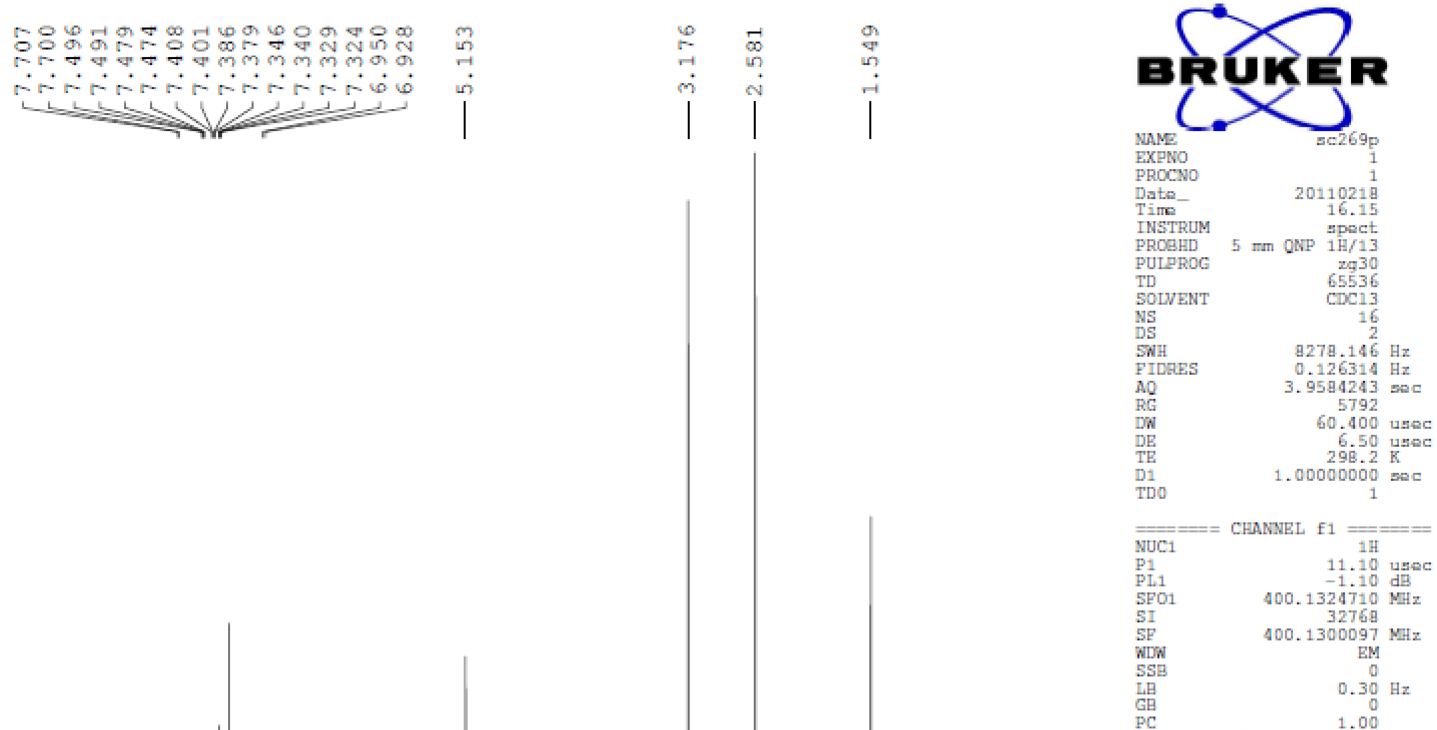
7.707
7.700
7.496
7.491
7.479
7.474
7.408
7.401
7.386
7.379
7.346
7.340
7.329
7.324
6.950
6.928
5.153
3.176
2.581
1.549
BRUKER
NAME sc269p
EXPNO 1
PROCNO 1
Date_ 20110218
Time 16.15
INSTRUM spect
PROBHD 5 mm QNP 1H/13
PULPROG zg30
TD 65536
SOLVENT CDCl3
NS 16
DS 2
SWH 8278.146 Hz
FIDRES 0.126314 Hz
AQ 3.9584243 sec
RG 5792
DW 60.400 usec
DE 6.50 usec
TE 298.2 K
D1 1.00000000 sec
TD0 1
======== CHANNEL f1 ========
NUC1 1H
P1 11.10 usec
PL1 -1.10 dB
SFO1 400.1324710 MHz
SI 32768
SF 400.1300097 MHz
WDW EM
SSB 0
LB 0.30 Hz
GB 0
PC 1.00
9
8
7
6
5
4
3
2
1
0
ppm
1.00
2.10
1.07
2.01
1.03
2.06
2.98
3.04
1.81

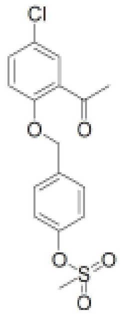
Cl
O
O
O
S
O
O

43

4-[(2-acetyl-4-chlorophenoxy) methyl] phenylmethanesulfonate

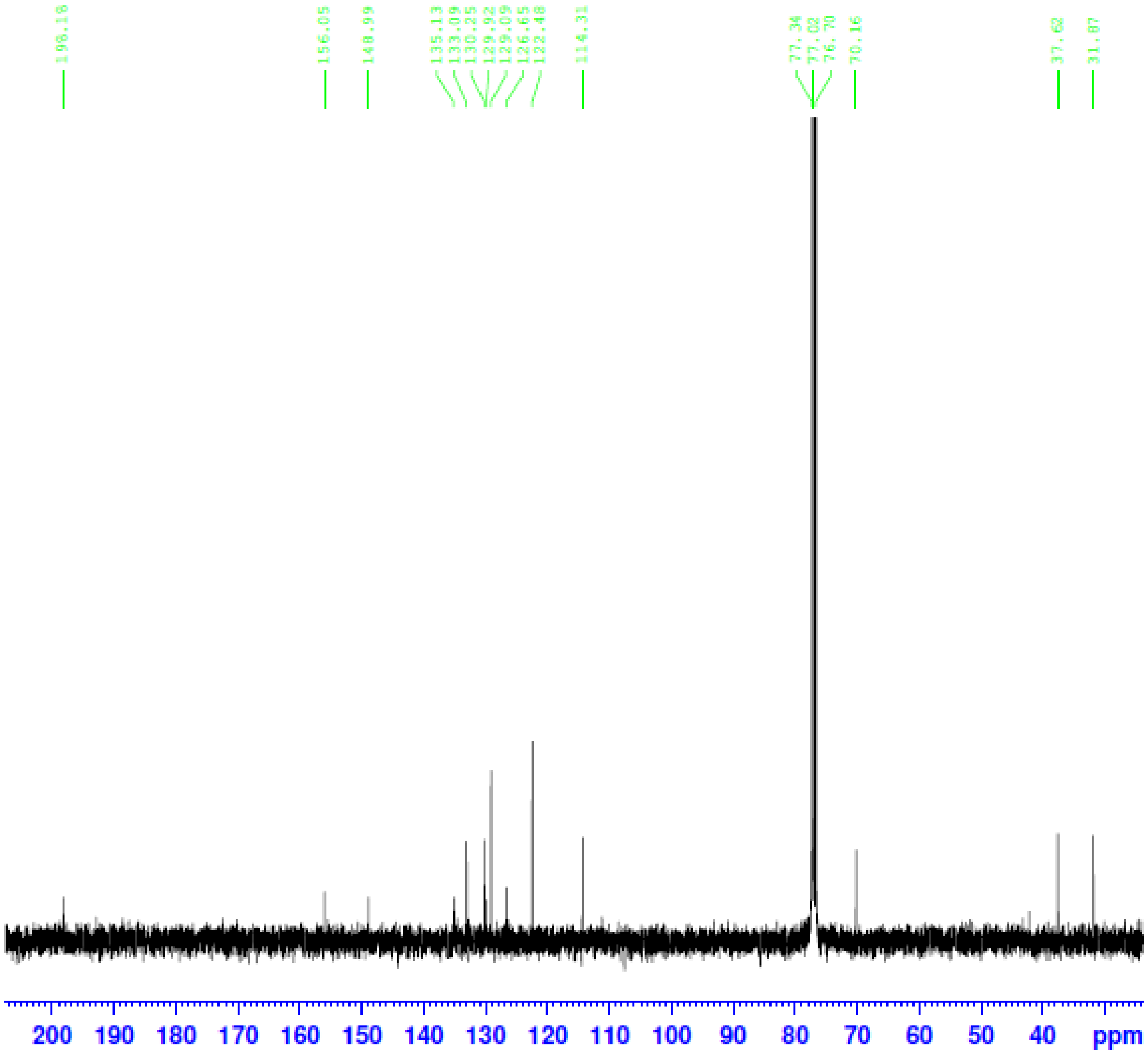

	69p
	2
	1
Date_	20110219
Time	2.19
INSTRUM	spect
PROBHD	5 mm QNP 1H/13
PULPROG	zgpg30
TD	65536
SOLVENT	CDCl3
NS	1024
DS	4
SWH	23980.814 Hz
FIDRES	0.365918 Hz
AQ	1.3664756 sec
RG	5792
DW	20.850 usec
DE	6.50 usec
TE	298.2 K
D1	2.00000000 sec
D11	0.03000000 sec
TD0	1
======== CHANNEL f1 ========	
NUC1	13C
P1	9.38 usec
PL1	0.00 dB
SFO1	100.6228298 MHz
======== CHANNEL f2 ========	
CPDPRG2	waltz16
NUC2	1H
PCPD2	80.00 usec
PL2	-1.10 dB
PL12	16.06 dB
PL13	21.00 dB
SFO2	400.1316005 MHz
SI	32768
SF	100.6127690 MHz
WDW	EM
SSB	0
LB	1.00 Hz
GB	0
PC	0.50

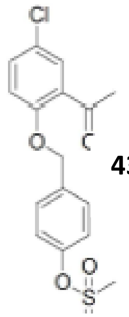

43

5-chloro-2-({4-[(methyl sulfonyl)oxy]benzyl}oxy)phenyl acetate

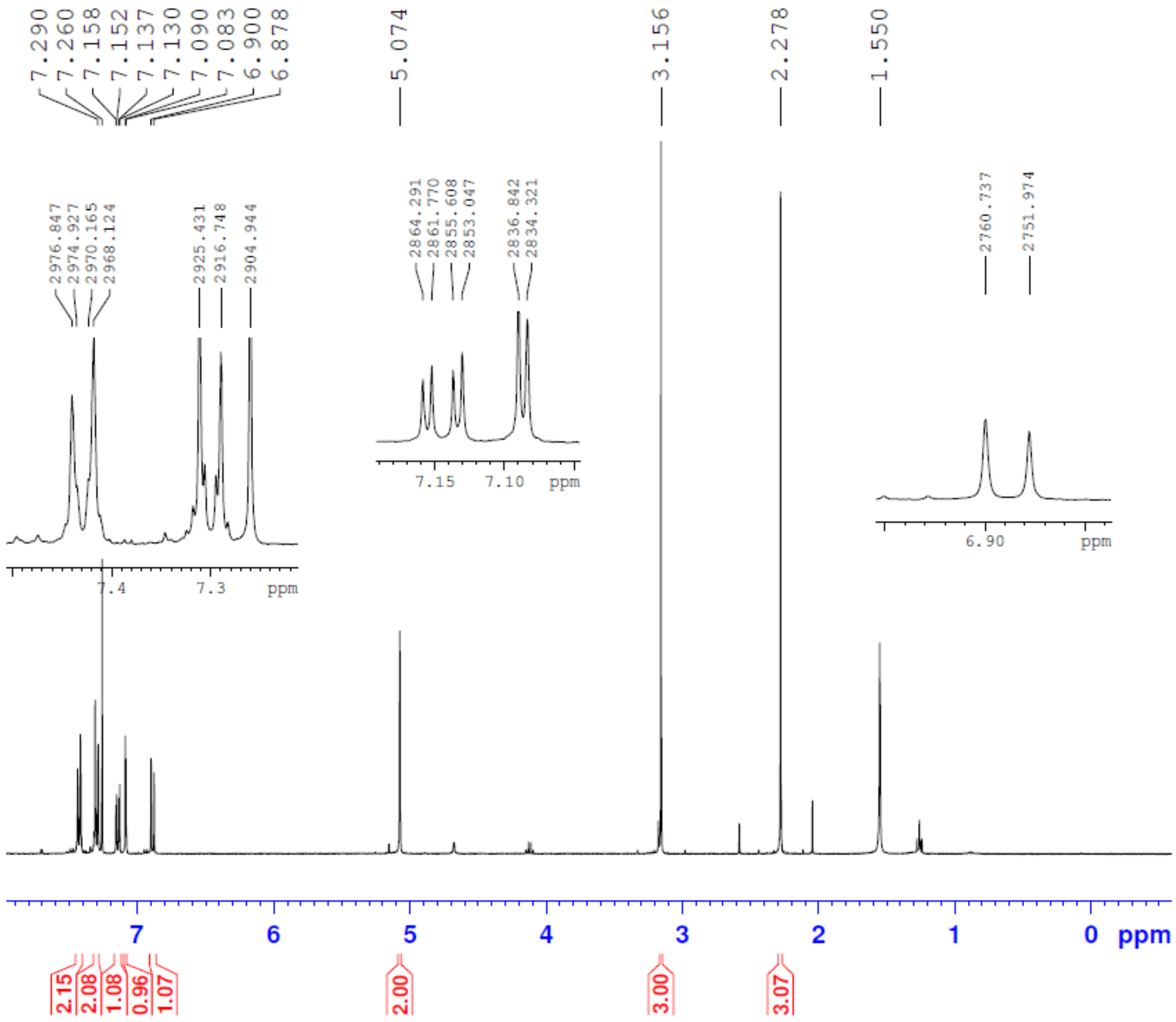

BRUKER

NAME	sc271p spot2
EXPNO	1
PROCNO	1
Date_	20110224
Time	17.02
INSTRUM	spect
PROBHD	5 mm QNP 1H/13
PULPROG	zg30
TD	65536
SOLVENT	CDCl3
NS	16
DS	2
SWH	8278.146 Hz
FIDRES	0.126314 Hz
AQ	3.9584243 sec
RG	5792
DW	60.400 usec
DE	6.50 usec
TE	298.2 K
D1	1.00000000 sec
TD0	1

======== CHANNEL f1 ========

NUC1	1H
P1	11.10 usec
PL1	-1.10 dB
SFO1	400.1324710 MHz
SI	32768
SF	400.1300095 MHz
WDW	EM
SSB	0
LB	0.30 Hz
GB	0
PC	1.00

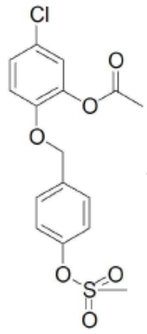

44

5-chloro-2-({4-[(methyl sulfonyl)oxy]benzyl}oxy)phenyl acetate

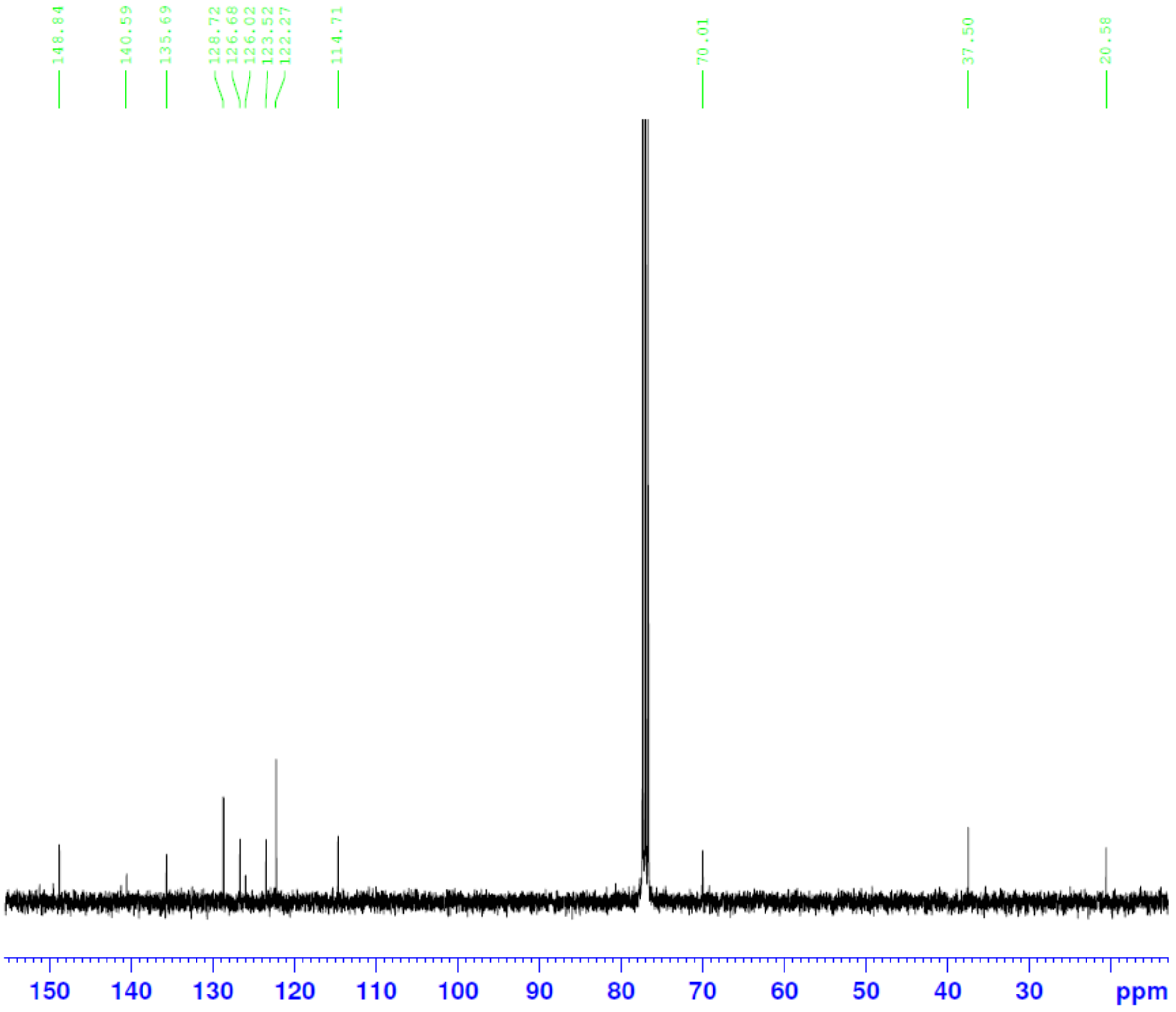

BRUKER

```
[illegible]
PROCNO                 1
Date_           20110225
Time                3.10
INSTRUM            spect
PROBHD   5 mm QNP 1H/13
PULPROG          zgpg30
TD                 65536
SOLVENT            CDCl3
NS                  1024
DS                     4
SWH           23980.814 Hz
FIDRES         0.365918 Hz
AQ            1.3664756 sec
RG                  5792
DW                20.850 usec
DE                  6.50 usec
TE                 298.2 K
D1            2.00000000 sec
D11           0.03000000 sec
TDO                    1

======== CHANNEL f1 ========
NUC1                 13C
P1                  9.38 usec
PL1                 0.00 dB
SFO1         100.6228298 MHz

======== CHANNEL f2 ========
CPDPRG2          waltz16
NUC2                  1H
PCPD2              80.00 usec
PL2                -1.10 dB
PL12               16.06 dB
PL13               21.00 dB
SFO2         400.1316005 MHz
SI                 32768
SF           100.6127690 MHz
WDW                   EM
SSB                    0
LB                  1.00 Hz
GB                     0
PC                   .40
```

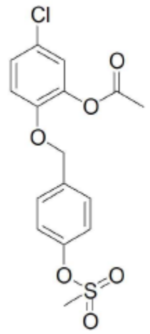

44

4-fluoro-2-methoxy-N-(4-methoxybenzyl)aniline

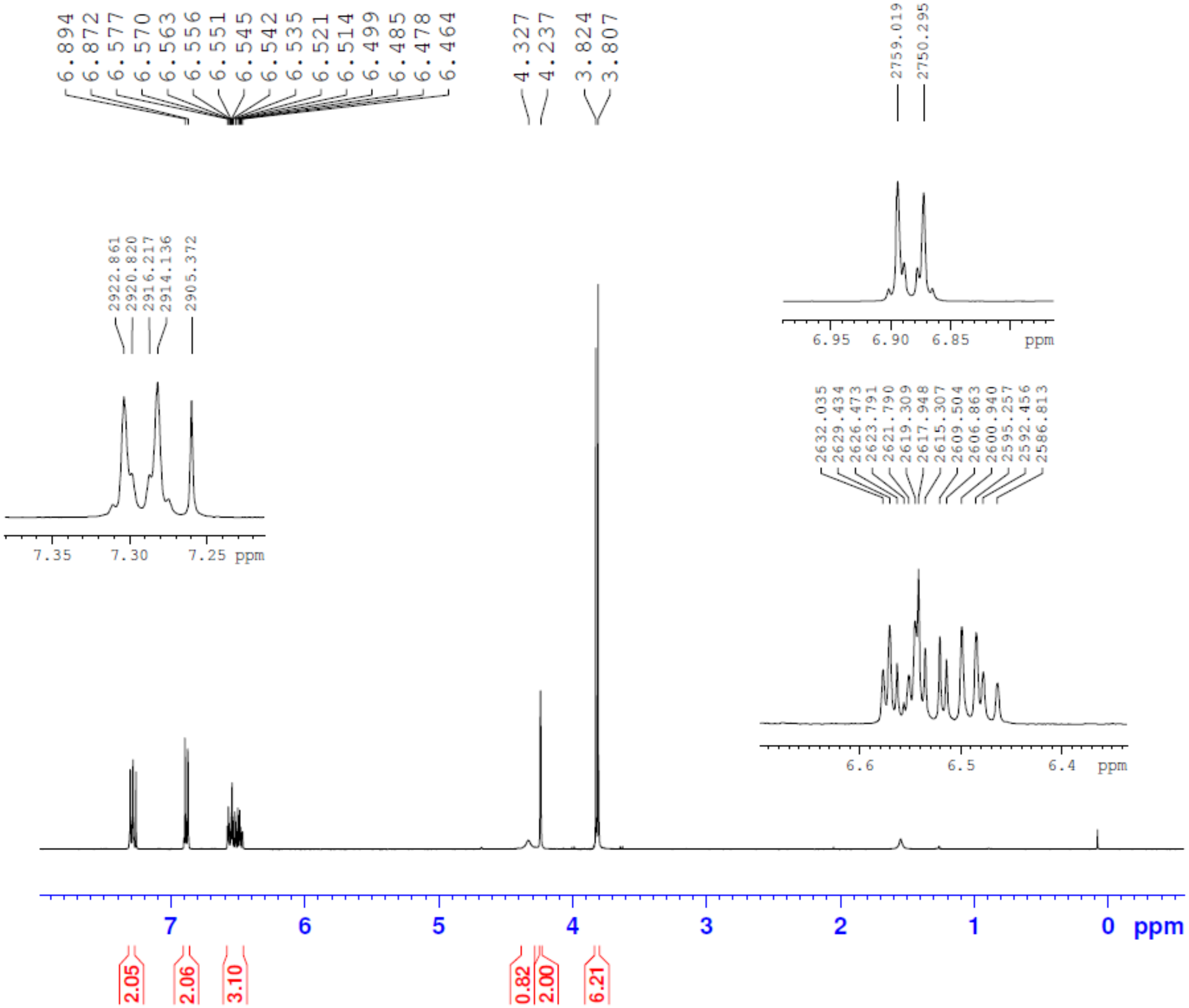

BRUKER

```
NAME       s_che.272recolumn
EXPNO                      1
PROCNO                     1
Date_               20110304
Time                   11.31
INSTRUM               dpx400
PROBHD      5 mm PABBO BB/
PULPROG                 zg30
TD                     65536
SOLVENT                CDCl3
NS                        16
DS                         2
SWH                 8223.685 Hz
FIDRES              0.125483 Hz
AQ                 3.9846387 sec
RG                     645.1
DW                    60.800 usec
DE                      6.00 usec
TE                     298.0 K
D1                1.00000000 sec
TD0                        1

======== CHANNEL f1 ========
NUC1                      1H
P1                     13.40 usec
PL1                    -6.00 dB
SFO1             400.2024712 MHz
SI                     65536
SF               400.2000130 MHz
WDW                       EM
SSB                        0
LB                      0.30 Hz
GB                         0
PC                      1.00
```

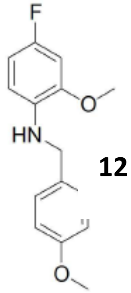

12

4-fluoro-2-methoxy-N-(4-methoxybenzyl)aniline

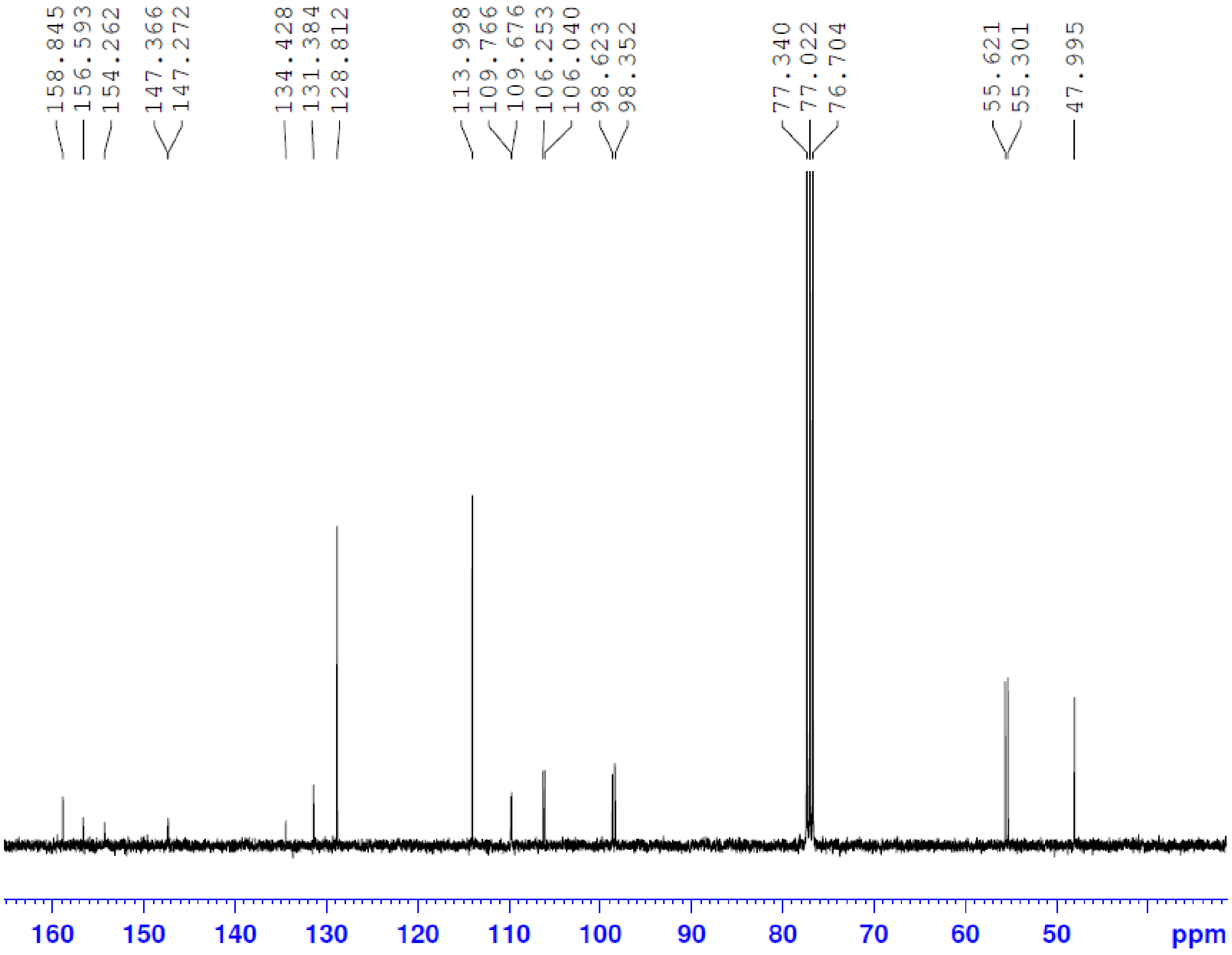

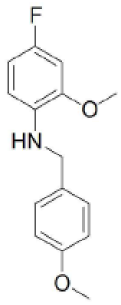

12

4-fluoro-2-methoxy-N-(4-methoxybenzyl)aniline

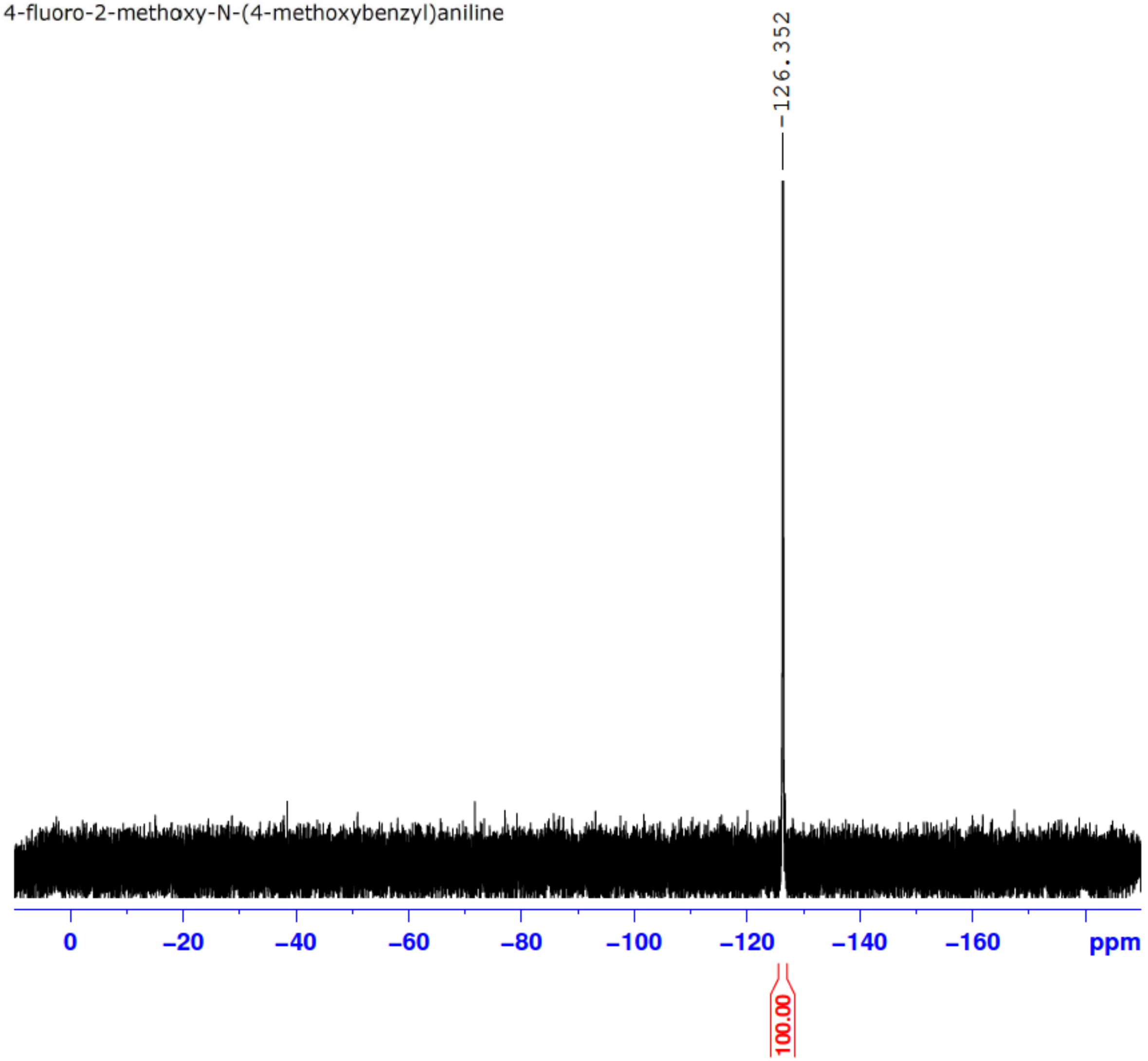

NAME	s_che.272spot2ppt	
EXPNO	2	
PROCNO	1	
Date_	20110305	
Time	8.14	
INSTRUM	av400	
PROBHD	5 mm PABBO BB-	
PULPROG	zg	
TD	262144	
SOLVENT	CDCl3	
NS	64	
DS	2	
SWH	75187.969	Hz
FIDRES	0.286819	Hz
AQ	1.7433076	sec
RG	2048	
DW	6.650	usec
DE	6.50	usec
TE	298.2	K
D1	2.00000000	sec
TD0	1	
======== CHANNEL f1 ========		
NUC1	19F	
P1	10.00	usec
PL1	3.00	dB
PL1W	4.67061329	W
SFO1	376.4644798	MHz
SI	262144	
SF	376.4983670	MHz
WDW	EM	
SSB	0	
LB	0.80	Hz
GB	0	
PC	1.00	

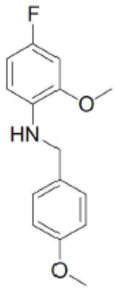

12

4-[(4-chloro-2-methoxyphenoxy)methyl]-1-(4-methoxyphenyl)-1-H-1,2,3 triazole

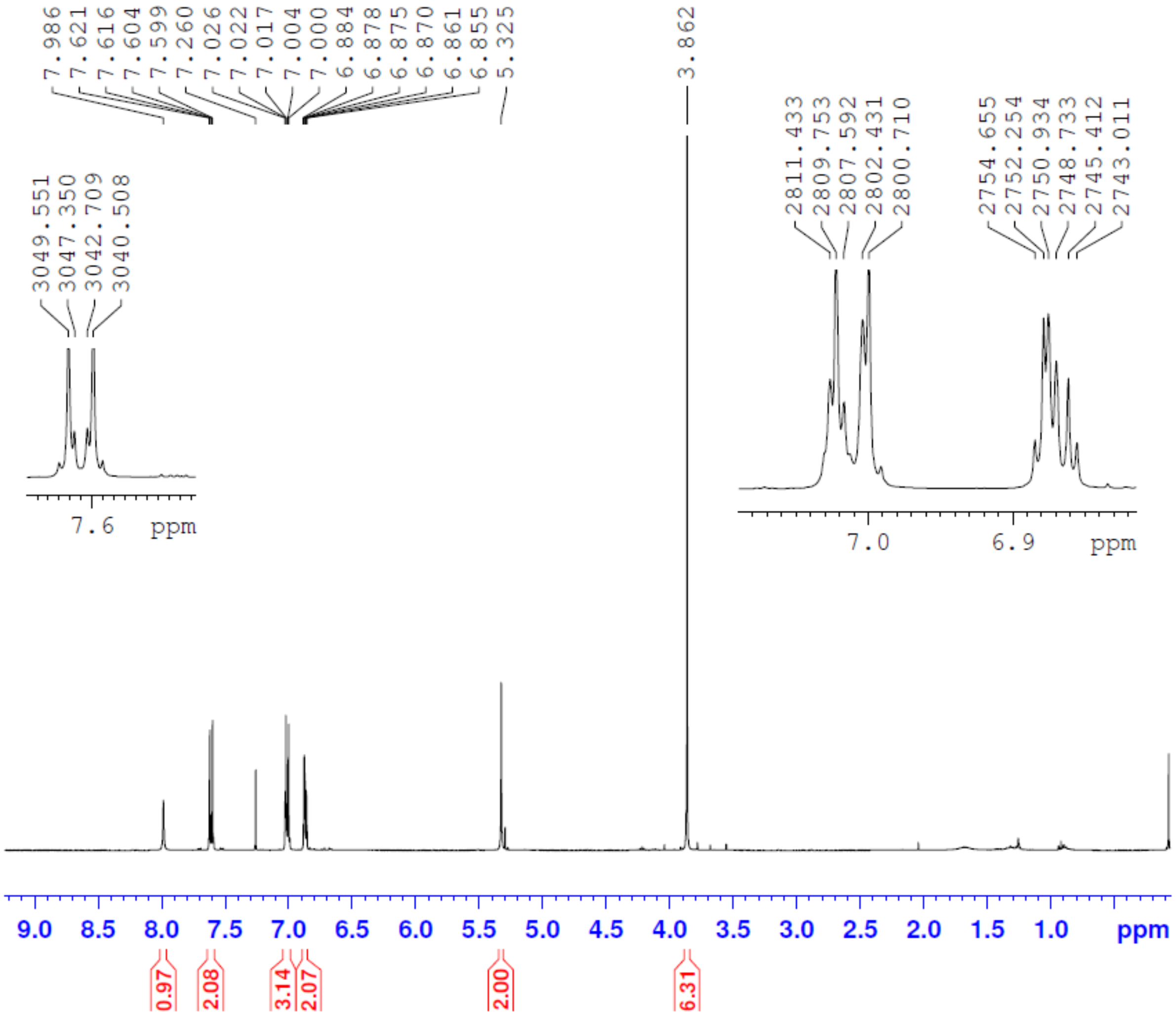

BRUKER

NAME	s_che.sample120p	
EXPNO	1	
PROCNO	1	
Date_	20100209	
Time	12.54	
INSTRUM	av400	
PROBHD	5 mm BBO BB-1H	
PULPROG	zg30	
TD	32768	
SOLVENT	CDCl3	
NS	128	
DS	2	
SWH	4789.272	Hz
FIDRES	0.146157	Hz
AQ	3.4210291	sec
RG	181	
DW	104.400	usec
DE	6.00	usec
TE	298.2	K
D1	1.00000000	sec
TD0	1	
======== CHANNEL f1 ========		
NUC1	1H	
P1	9.60	usec
PL1	-3.00	dB
PL1W	20.14772606	W
SFO1	400.1322007	MHz
SI	32768	
SF	400.1300094	MHz
WDW	EM	
SSB	0	
LB	0.30	Hz
GB	0	
PC	1.00	

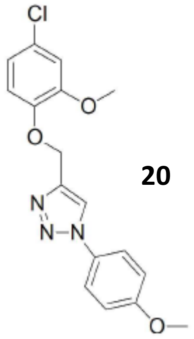

20

4-[(4-chloro-2-methoxyphenoxy)methyl]-1-(4-methoxyphenyl)-1-H-1,2,3 triazole

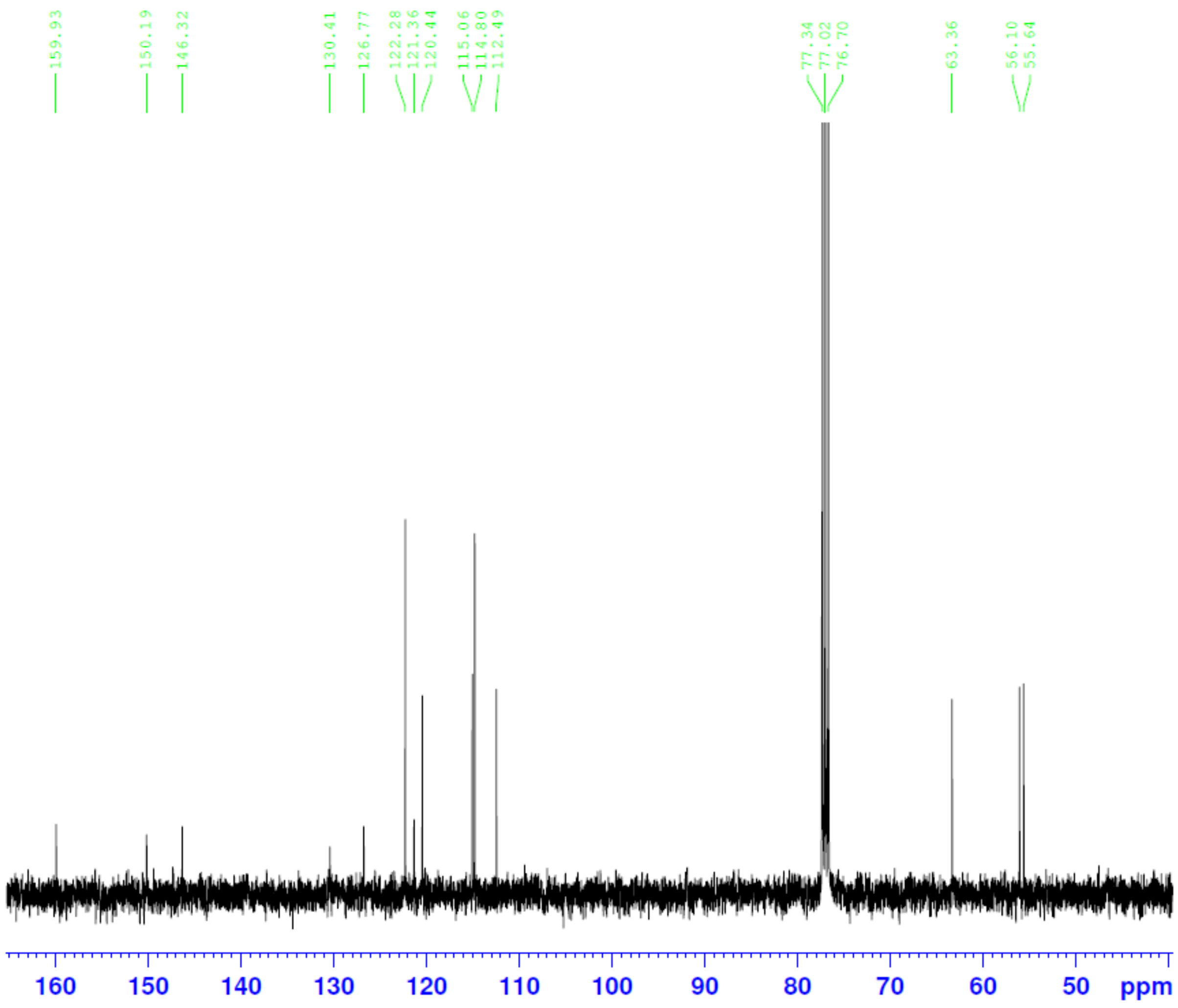


```
EXPNO                  1
PROCNO                 1
Date_           20110309
Time               18.47
INSTRUM            av400
PROBHD   5 mm PABBO BB-
PULPROG           zgpg30
TD                 32768
SOLVENT            CDCl3
NS                  1024
DS                     2
SWH            25125.629 Hz
FIDRES          0.766773 Hz
AQ             0.6521332 sec
RG               14596.5
DW                19.900 usec
DE                 10.00 usec
TE                 298.2 K
D1            1.00000000 sec
D11           0.03000000 sec
TD0                    1

======== CHANNEL f1 ========
NUC1                 13C
P1                  7.50 usec
PL1                -3.00 dB
PL1W         73.67452240 W
SFO1         100.6238350 MHz

======== CHANNEL f2 ========
CPDPRG2          waltz16
NUC2                  1H
PCPD2             100.00 usec
PL2                -2.00 dB
PL12               17.00 dB
PL13               19.30 dB
PL2W         16.00390816 W
PL12W         0.20147727 W
PL13W         0.11863863 W
SFO2         400.1316005 MHz
SI                 32768
SF           100.6127690 MHz
WDW                   EM
SSB                    0
LB                  1.00 Hz
GB                     0
PC                  [illegible]
```

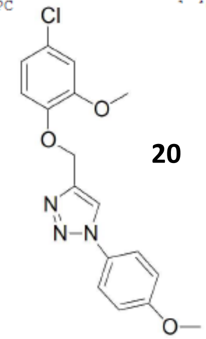

20

4-[(4-fluoro-2-methoxyphenoxy)methyl-1-(4methoxyphenyl)-1H-1,2,3-triazole

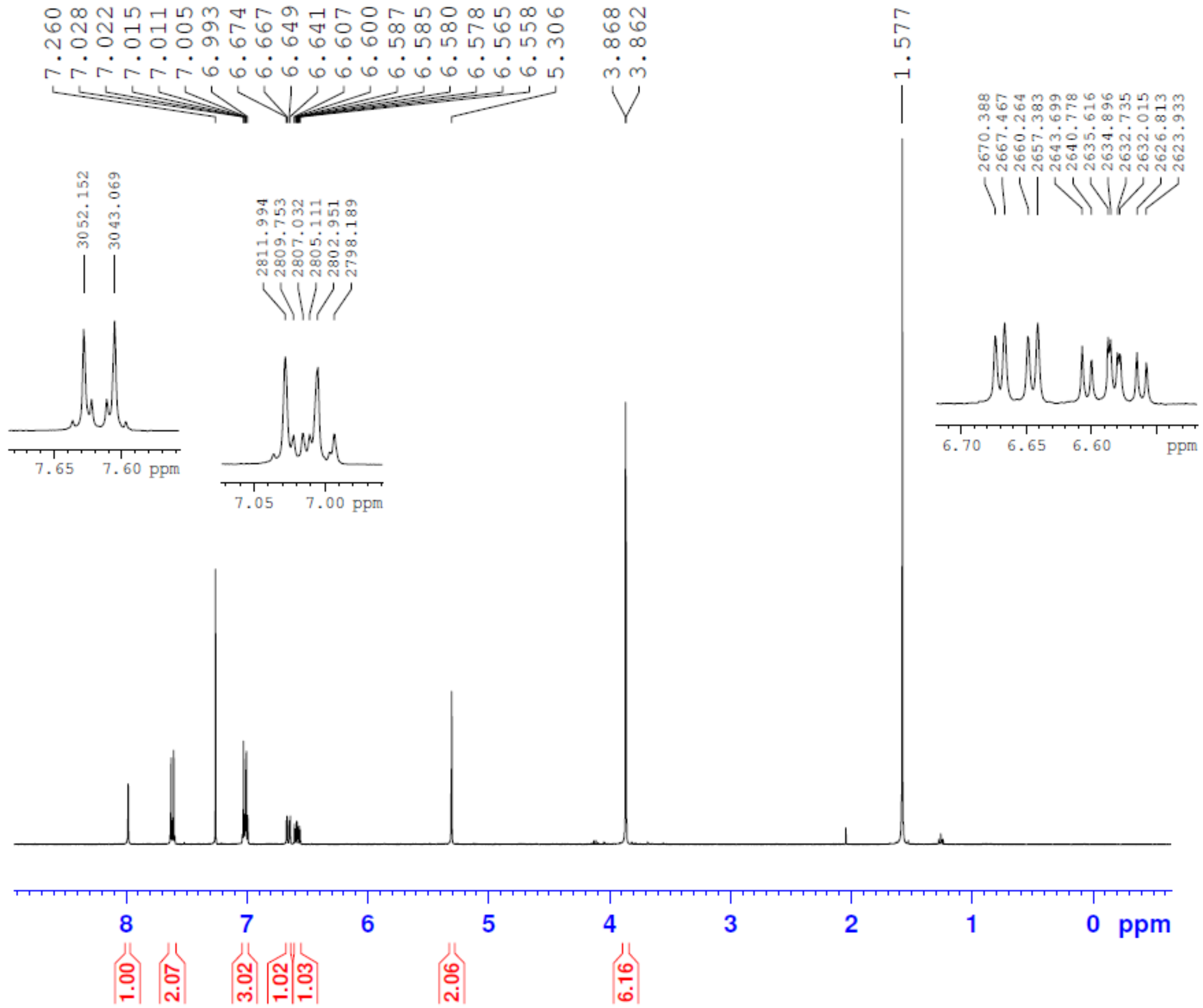

NAME	sc162pure
EXPNO	1
PROCNO	1
Date_	20100610
Time	16.15
INSTRUM	spect
PROBHD	5 mm QNP 1H/13
PULPROG	zg30
TD	65536
SOLVENT	CDCl3
NS	16
DS	2
SWH	8278.146 Hz
FIDRES	0.126314 Hz
AQ	3.9584243 sec
RG	1448
DW	60.400 usec
DE	6.50 usec
TE	298.0 K
D1	1.00000000 sec
TD0	1

======== CHANNEL f1 ========

NUC1	1H
P1	11.10 usec
PL1	-1.10 dB
SFO1	400.1324710 MHz
SI	32768
SF	400.1300095 MHz
WDW	EM
SSB	0
LB	0.30 Hz
GB	0
PC	0

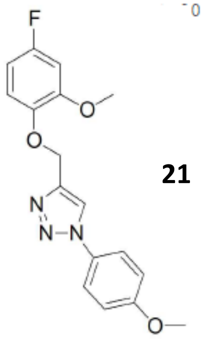

21

4-[(4-fluoro-2-methoxyphenoxy)methyl-1-(4methoxyphenyl)-1H-1,2,3-triazole

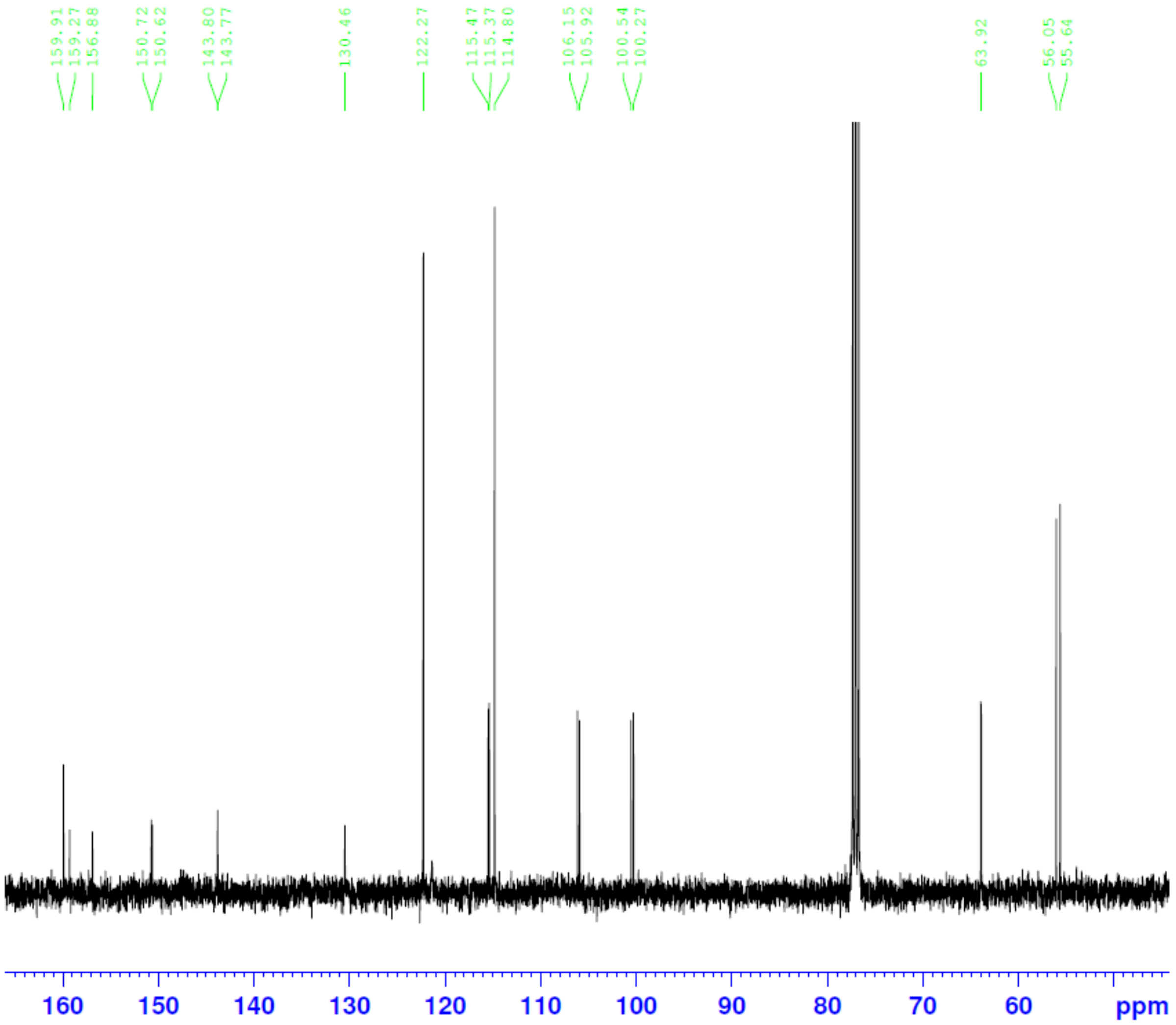

BRUKER

NAME	s_che.162p_2
EXPNO	1
PROCNO	1
Date_	20110309
Time	19.22
INSTRUM	av400
PROBHD	5 mm PABBO BB-
PULPROG	zgpg30
TD	32768
SOLVENT	CDCl3
NS	1024
DS	2
SWH	25125.629 Hz
FIDRES	0.766773 Hz
AQ	0.6521332 sec
RG	20642.5
DW	19.900 usec
DE	10.00 usec
TE	298.2 K
D1	1.00000000 sec
D11	0.03000000 sec
TD0	1
======== CHANNEL f1 ========	
NUC1	13C
P1	7.50 usec
PL1	-3.00 dB
PL1W	73.67452240 W
SFO1	100.6238350 MHz
======== CHANNEL f2 ========	
CPDPRG2	waltz16
NUC2	1H
PCPD2	100.00 usec
PL2	-2.00 dB
PL12	17.00 dB
PL13	19.30 dB
PL2W	16.00390816 W
PL12W	0.20147727 W
PL13W	0.11863863 W
SFO2	400.1316005 MHz
SI	32768
SF	100.6127690 MHz
WDW	EM
SSB	0
LB	[illegible]
GB	
PC	

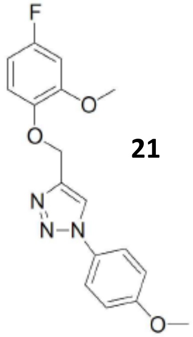

21

4-[(4-fluoro-2-methoxyphenoxy)methyl-1-(4methoxyphenyl)-1H-1,2,3-triazole

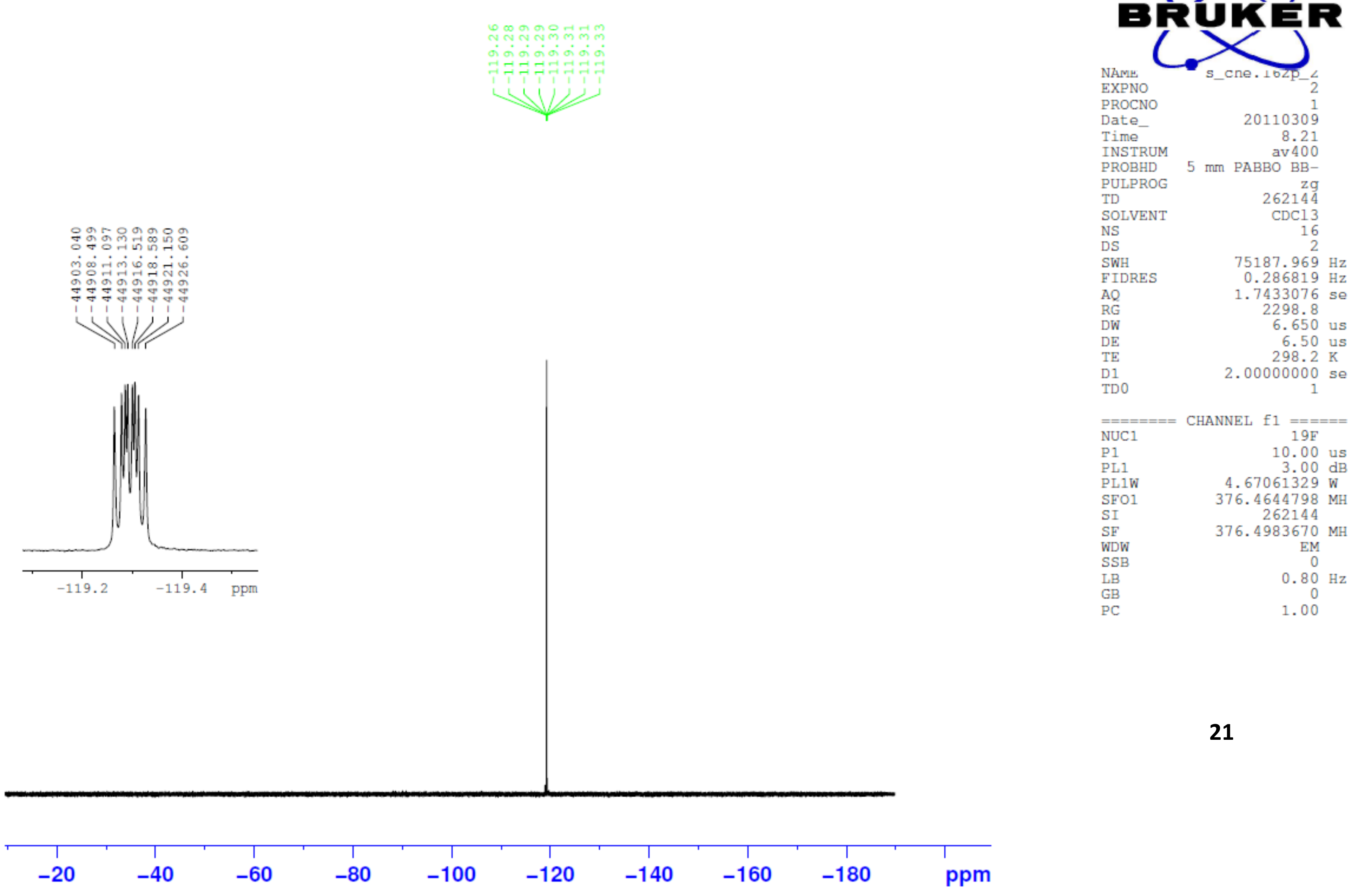

21

4-[(4-bromo-2-methoxyphenoxy)methyl-1-(4methoxyphenyl)-1H-1,2,3-triazole

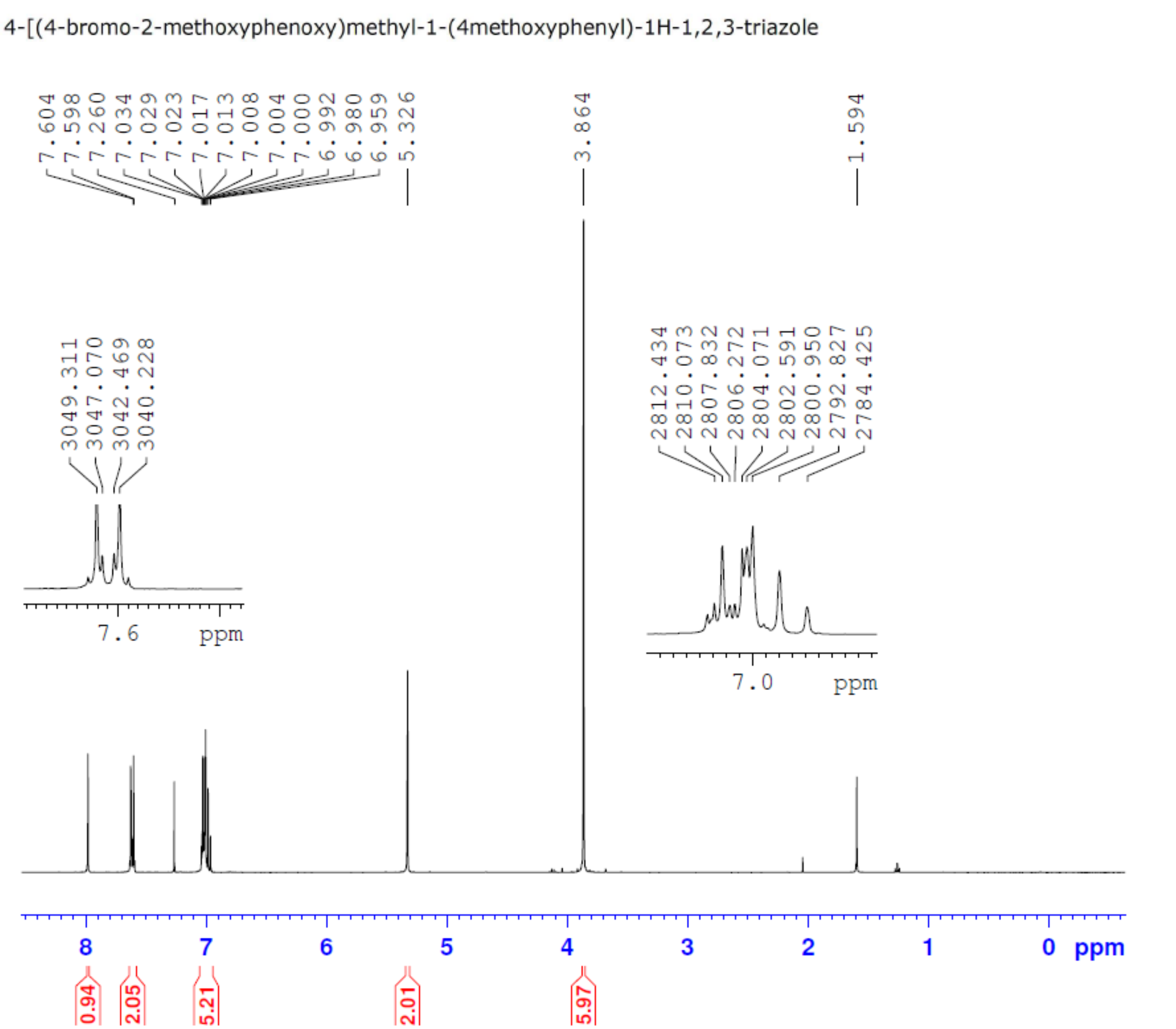

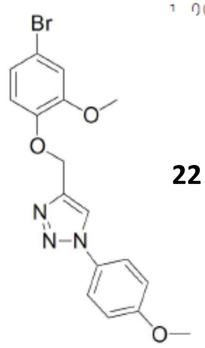

22

4-[(4-bromo-2-methoxyphenoxy)methyl-1-(4methoxyphenyl)-1H-1,2,3-triazole

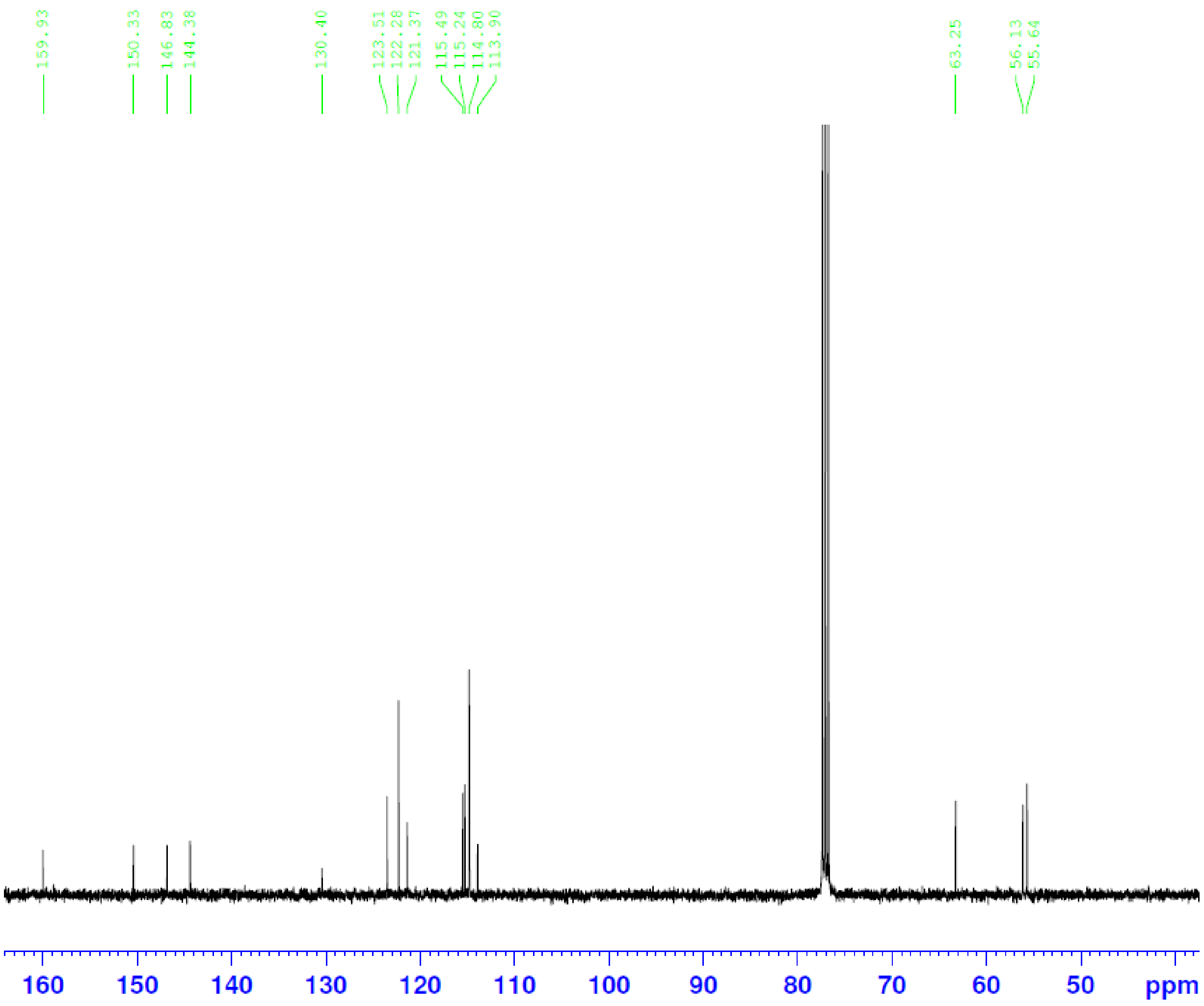

NAME	sc163p_2
EXPNO	2
PROCNO	1
Date_	20110310
Time	23.08
INSTRUM	spect
PROBHD	5 mm QNP 1H/13
PULPROG	zgpg30
TD	65536
SOLVENT	CDCl3
NS	1024
DS	4
SWH	23980.814 Hz
FIDRES	0.365918 Hz
AQ	1.3664756 sec
RG	8192
DW	20.850 usec
DE	6.50 usec
TE	298.2 K
D1	2.00000000 sec
D11	0.03000000 sec
TD0	1
======== CHANNEL f1 ========	
NUC1	13C
P1	9.38 usec
PL1	0.00 dB
SFO1	100.6228298 MHz
======== CHANNEL f2 ========	
CPDPRG2	waltz16
NUC2	1H
PCPD2	80.00 usec
PL2	-1.10 dB
PL12	16.06 dB
PL13	21.00 dB
SFO2	400.1316005 MHz
SI	32768
SF	100.6127690 MHz
WDW	EM
SSB	0
LB	1.00 Hz
GB	0
PC	[illegible]

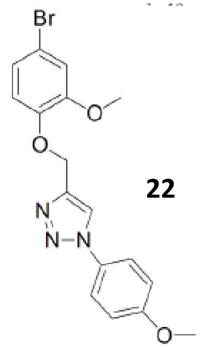

22

4-chloro-2-methoxy-N-{[1-4-methoxyphenyl)-1H-1,2,3 triazol-4-yl]methyl} aniline

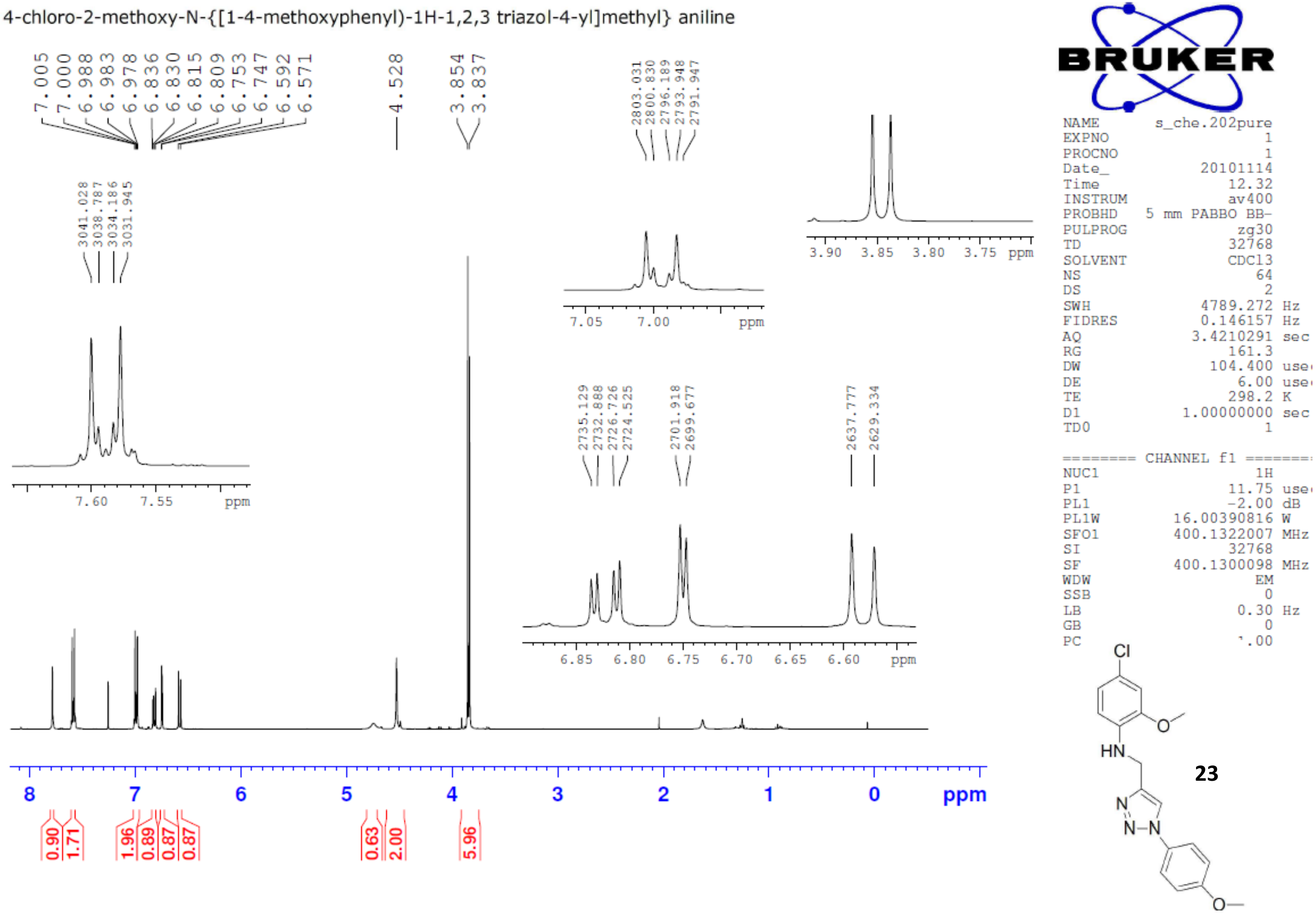

4-chloro-2-methoxy-N-{[1-4-methoxyphenyl)-1H-1,2,3 triazol-4-yl]methyl} aniline

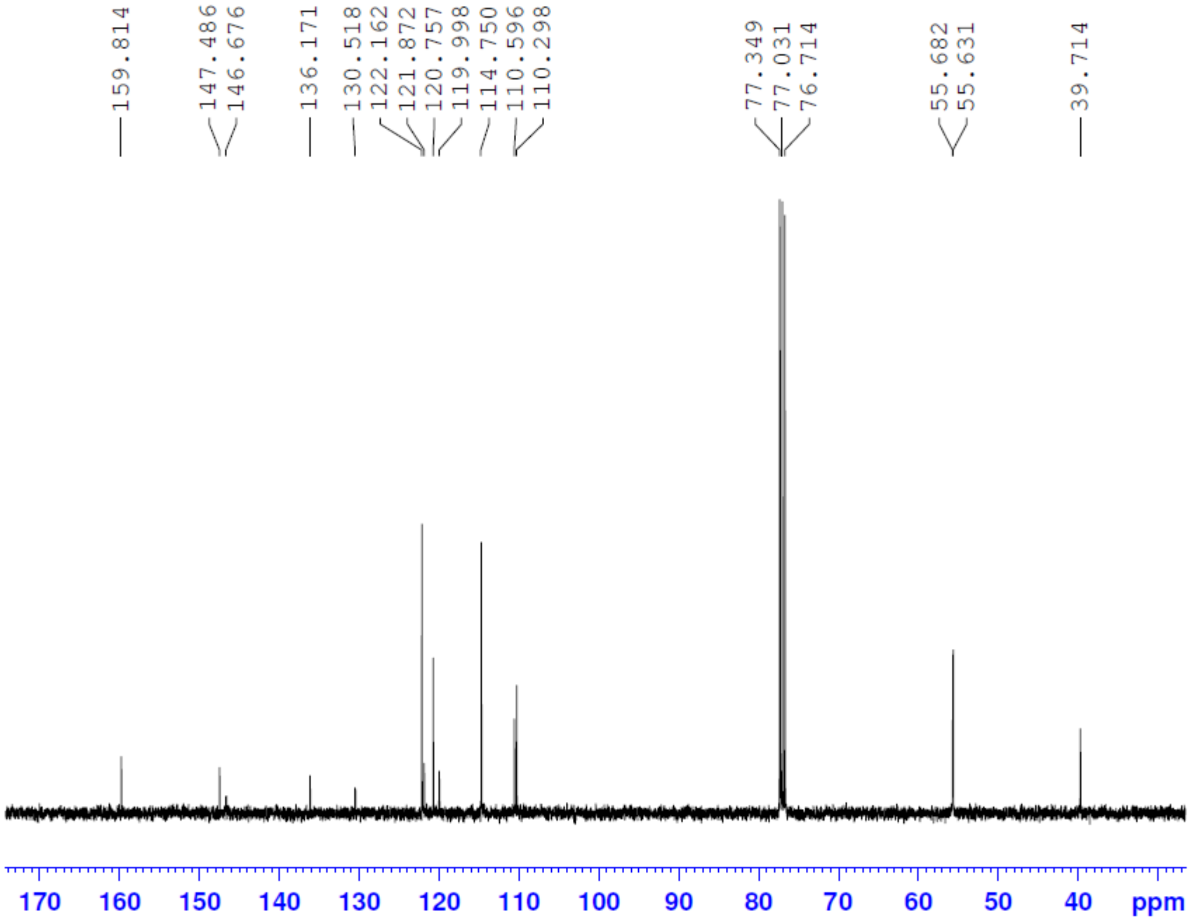


```
NAME          s_che.202pure
EXPNO                     2
PROCNO                    1
Date_              20101114
Time                  12.55
INSTRUM               av400
PROBHD     5 mm PABBO BB-
PULPROG             zgpg30
TD                    32768
SOLVENT               CDCl3
NS                      512
DS                        2
SWH              25125.629 Hz
FIDRES            0.766773 Hz
AQ               0.6521332 sec
RG                 20642.5
DW                  19.900 usec
DE                   10.00 usec
TE                   298.2 K
D1              1.00000000 sec
D11             0.03000000 sec
TD0                       1

======== CHANNEL f1 ========
NUC1                    13C
P1                     7.50 usec
PL1                   -3.00 dB
PL1W            73.67452240 W
SFO1            100.6238350 MHz

======== CHANNEL f2 ========
CPDPRG2             waltz16
NUC2                     1H
PCPD2                100.00 usec
PL2                   -2.00 dB
PL12                  17.00 dB
PL13                  19.30 dB
PL2W            16.00390816 W
PL12W            0.20147727 W
PL13W            0.11863863 W
SFO2            400.1316005 MHz
SI                    32768
SF              100.6127690 MHz
WDW                      EM
SSB                       0
LB                     1.00 Hz
GB
PC
```

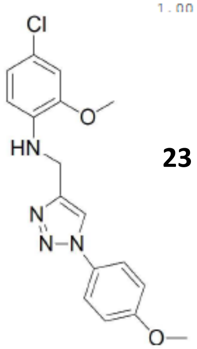

23

4-fluoro-2-methoxy-N-{[1-4-methoxyphenyl)-1H-1,2,3 triazol-4-yl]methyl} aniline

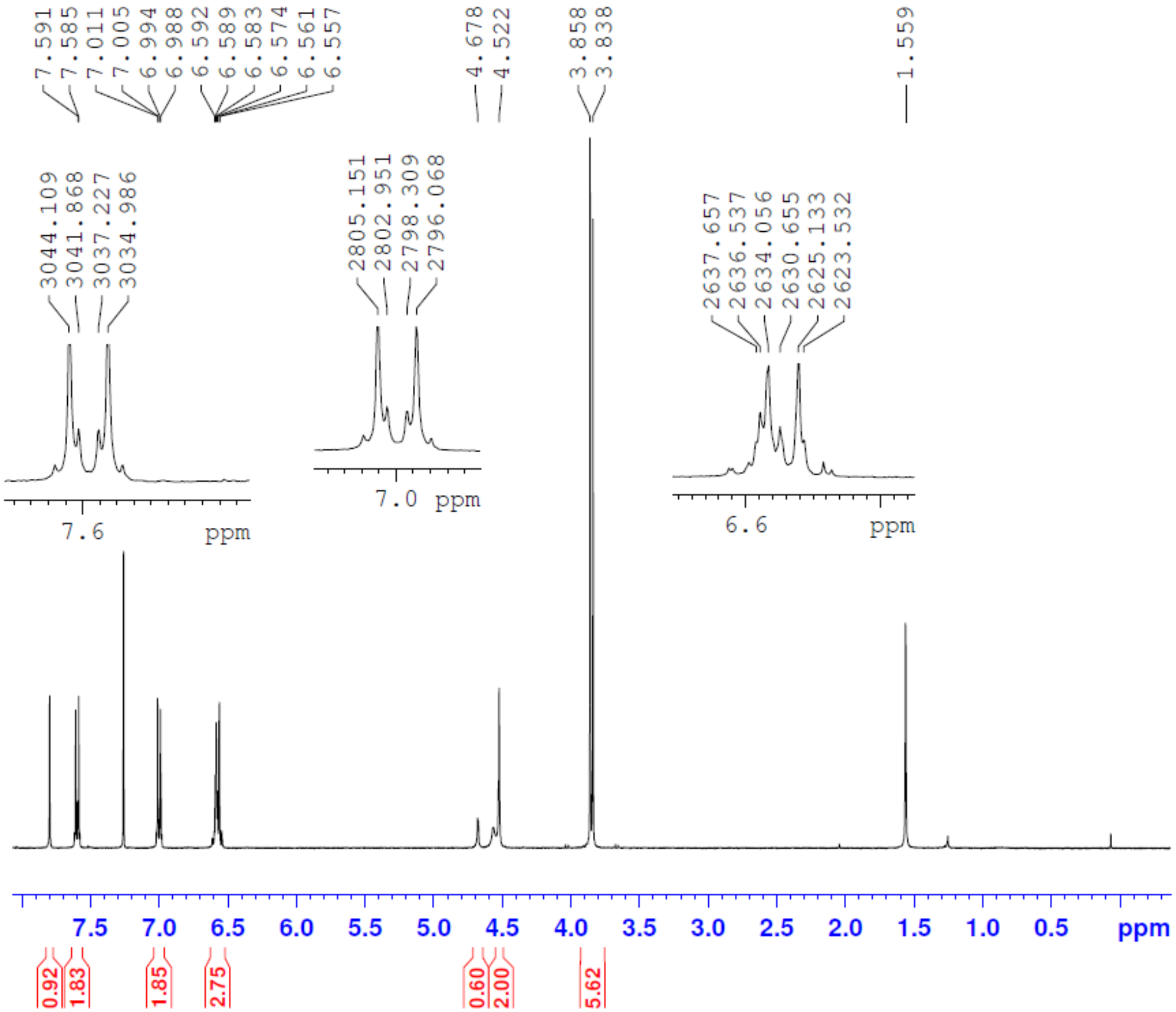

BRUKER

NAME	sc263spot3	
EXPNO	1	
PROCNO	1	
Date_	20110202	
Time	12.55	
INSTRUM	spect	
PROBHD	5 mm QNP 1H/13	
PULPROG	zg30	
TD	65536	
SOLVENT	CDCl3	
NS	16	
DS	2	
SWH	8278.146	Hz
FIDRES	0.126314	Hz
AQ	3.9584243	sec
RG	5792	
DW	60.400	usec
DE	6.50	usec
TE	298.2	K
D1	1.00000000	sec
TD0	1	
======== CHANNEL f1 ========		
NUC1	1H	
P1	11.10	usec
PL1	-1.10	dB
SFO1	400.1324710	MHz
SI	32768	
SF	400.1300102	MHz
WDW	EM	
SSB	0	
LB	0.30	Hz
GB	0	
PC	1.00	

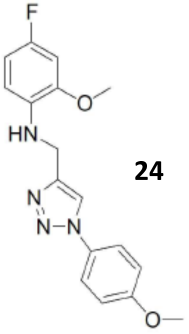

24

4-fluoro-2-methoxy-N-{[1-4-methoxyphenyl)-1H-1,2,3 triazol-4-yl]methyl} aniline

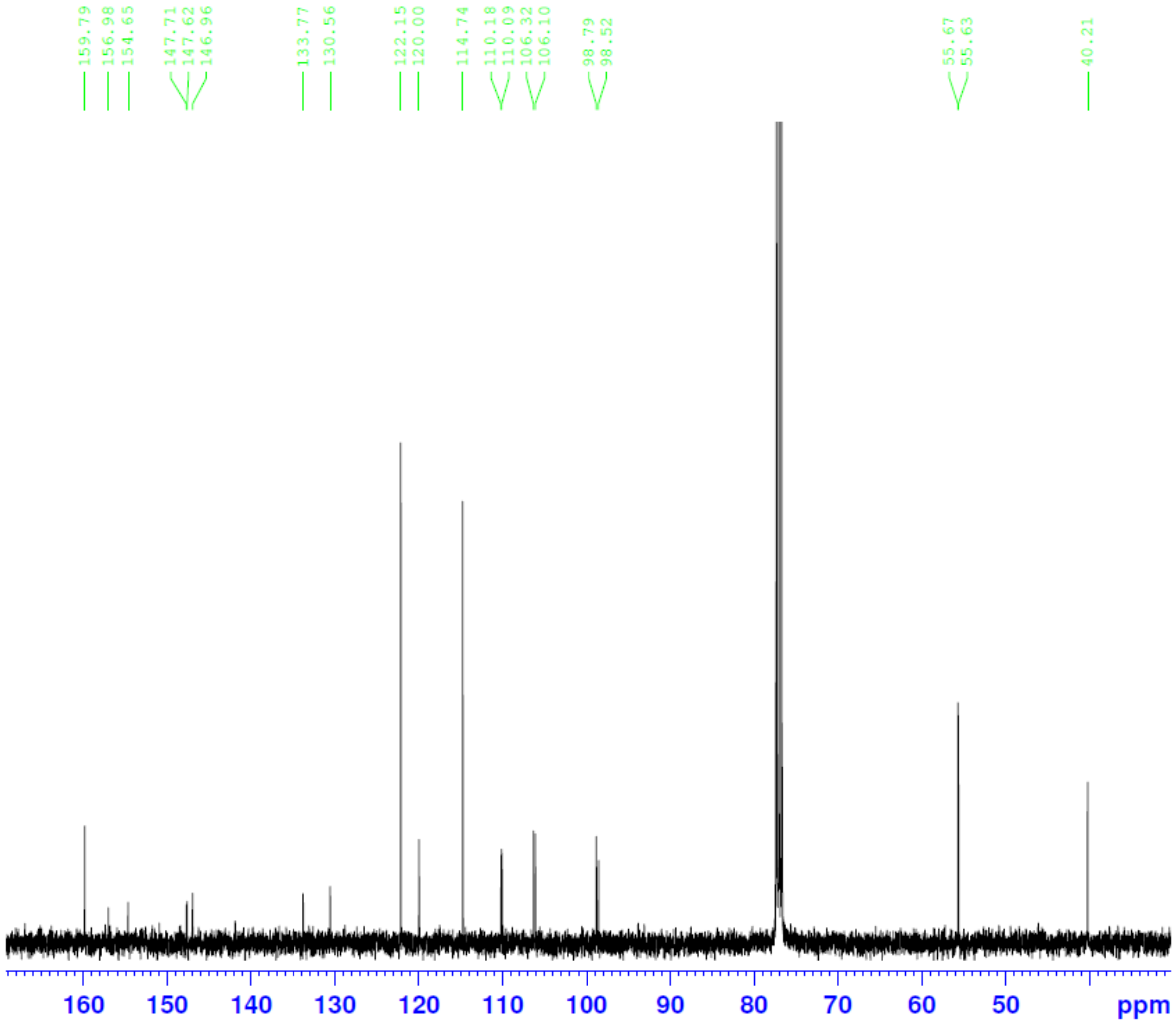


```
EXPNO                   2
PROCNO                  1
Date_            20110315
Time                 2.23
INSTRUM             spect
PROBHD   5 mm QNP 1H/13
PULPROG           zgpg30
TD                  65536
SOLVENT             CDCl3
NS                   1024
DS                      4
SWH             23980.814 Hz
FIDRES           0.365918 Hz
AQ              1.3664756 sec
RG                   4096
DW                 20.850 usec
DE                   6.50 usec
TE                  298.2 K
D1             2.00000000 sec
D11            0.03000000 sec
TD0                     1

======== CHANNEL f1 ========
NUC1                  13C
P1                   9.38 usec
PL1                  0.00 dB
SFO1          100.6228298 MHz

======== CHANNEL f2 ========
CPDPRG2           waltz16
NUC2                   1H
PCPD2               80.00 usec
PL2                 -1.10 dB
PL12                16.06 dB
PL13                21.00 dB
SFO2          400.1316005 MHz
SI                  32768
SF            100.6127690 MHz
WDW                    EM
SSB                     0
LB                   1.00 Hz
GB                      0
PC                   1.40
```

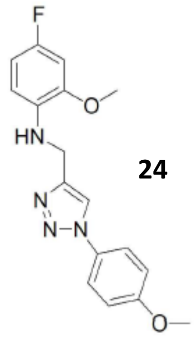

24

4-fluoro-2-methoxy-N-{[1-4-methoxyphenyl)-1H-1,2,3 triazol-4-yl]methyl} aniline

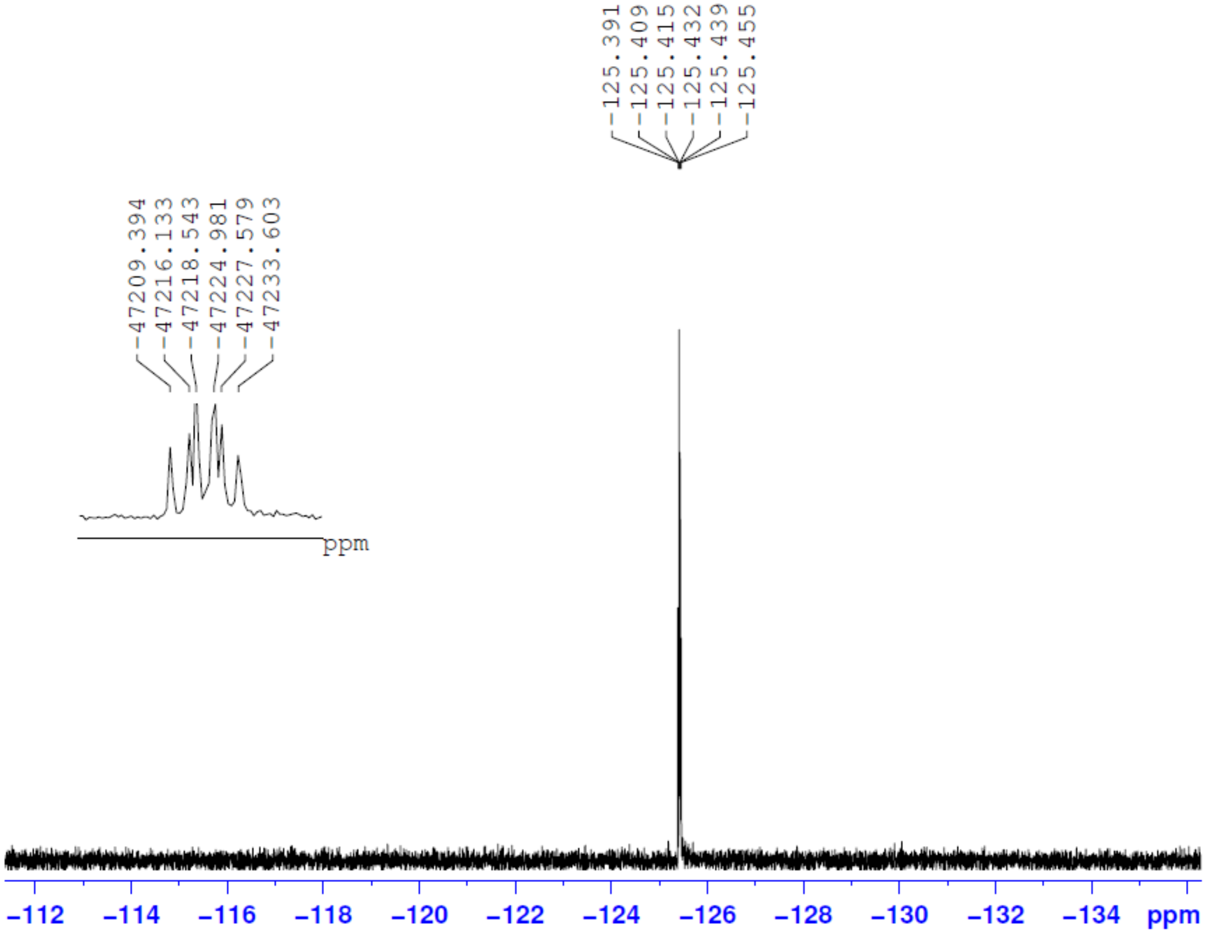

BRUKER

NAME	sc263_NMR2	
EXPNO	3	
PROCNO	1	
Date_	20110315	
Time	2.24	
INSTRUM	spect	
PROBHD	5 mm QNP 1H/13	
PULPROG	zgflqn	
TD	131072	
SOLVENT	CDCl3	
NS	16	
DS	4	
SWH	75187.969	Hz
FIDRES	0.573639	Hz
AQ	0.8716788	sec
RG	32768	
DW	6.650	usec
DE	6.50	usec
TE	298.3	K
D1	1.00000000	sec
TD0	1	
======== CHANNEL f1 ========		
NUC1	19F	
P1	13.00	usec
PL1	0.00	dB
SFO1	376.4607164	MHz
SI	65536	
SF	376.4983660	MHz
WDW	no	
SSB	0	
LB	0.00	Hz
GB	0	
PC	1.00	

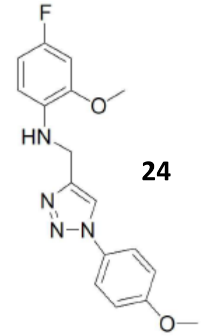

24

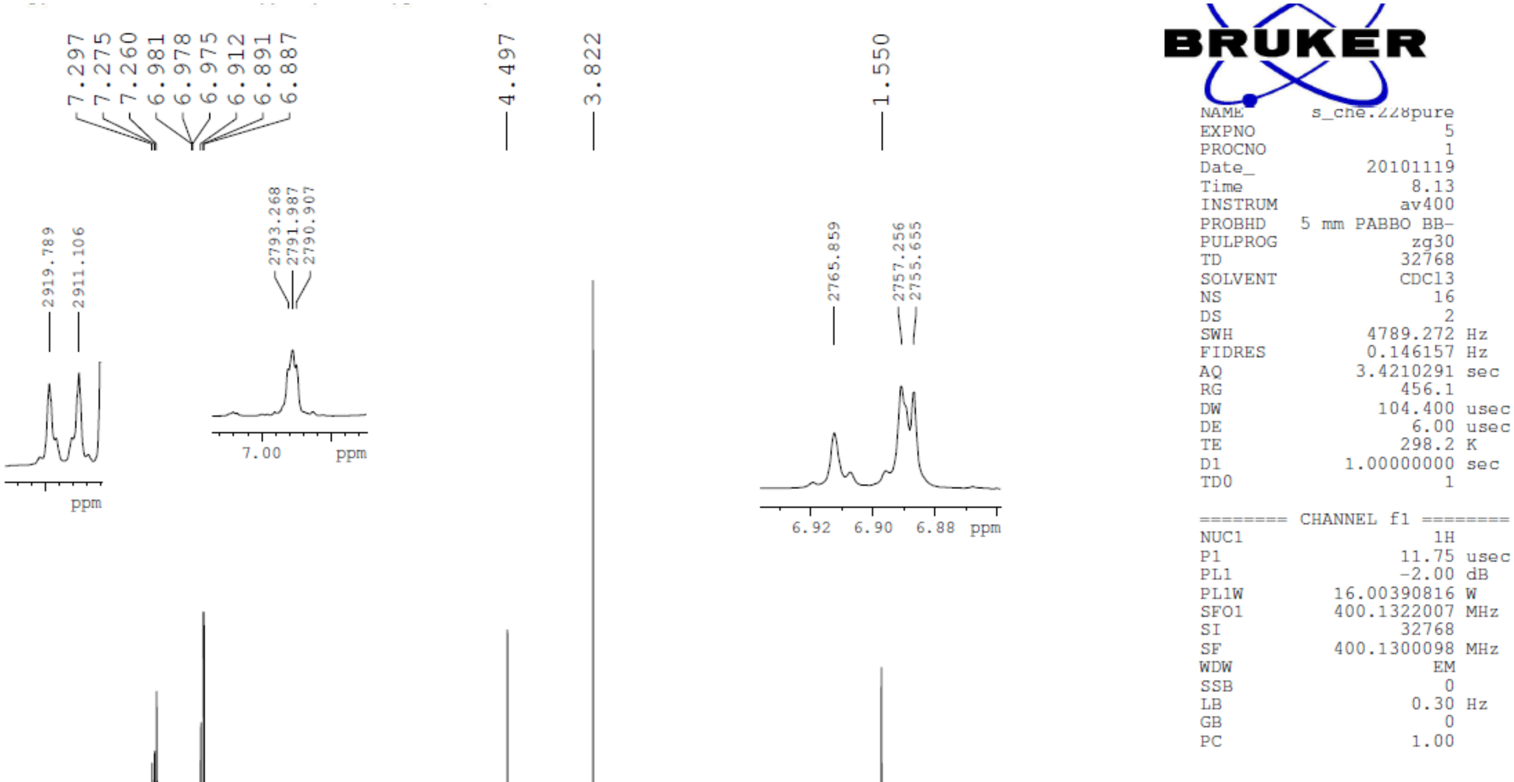
7.297
7.275
7.260
6.981
6.978
6.975
6.912
6.891
6.887
4.497
3.822
1.550
2919.789
2911.106
2793.268
2791.987
2790.907
7.00
2765.859
2757.256
2755.655
6.92 6.90 6.88 ppm
8 7 6 5 4 3 2 1 0 ppm
2.58
0.94
3.85
2.00
3.01
NAME s_che.228pure
EXPNO 5
PROCNO 1
Date_ 20101119
Time 8.13
INSTRUM av400
PROBHD 5 mm PABBO BB-
PULPROG zg30
TD 32768
SOLVENT CDCl3
NS 16
DS 2
SWH 4789.272 Hz
FIDRES 0.146157 Hz
AQ 3.4210291 sec
RG 456.1
DW 104.400 usec
DE 6.00 usec
TE 298.2 K
D1 1.00000000 sec
TD0 1
======== CHANNEL f1 ========
NUC1 1H
P1 11.75 usec
PL1 -2.00 dB
PL1W 16.00390816 W
SFO1 400.1322007 MHz
SI 32768
SF 400.1300098 MHz
WDW EM
SSB 0
LB 0.30 Hz
GB 0
PC 1.00

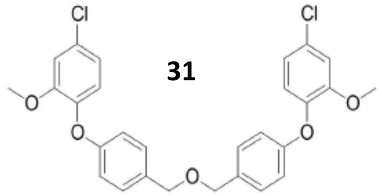
Cl
Cl
O
O
O
O
O

31

Bis [(4'-chloro-2'-methoxy)-4-phenoxy]dibenzyl ether

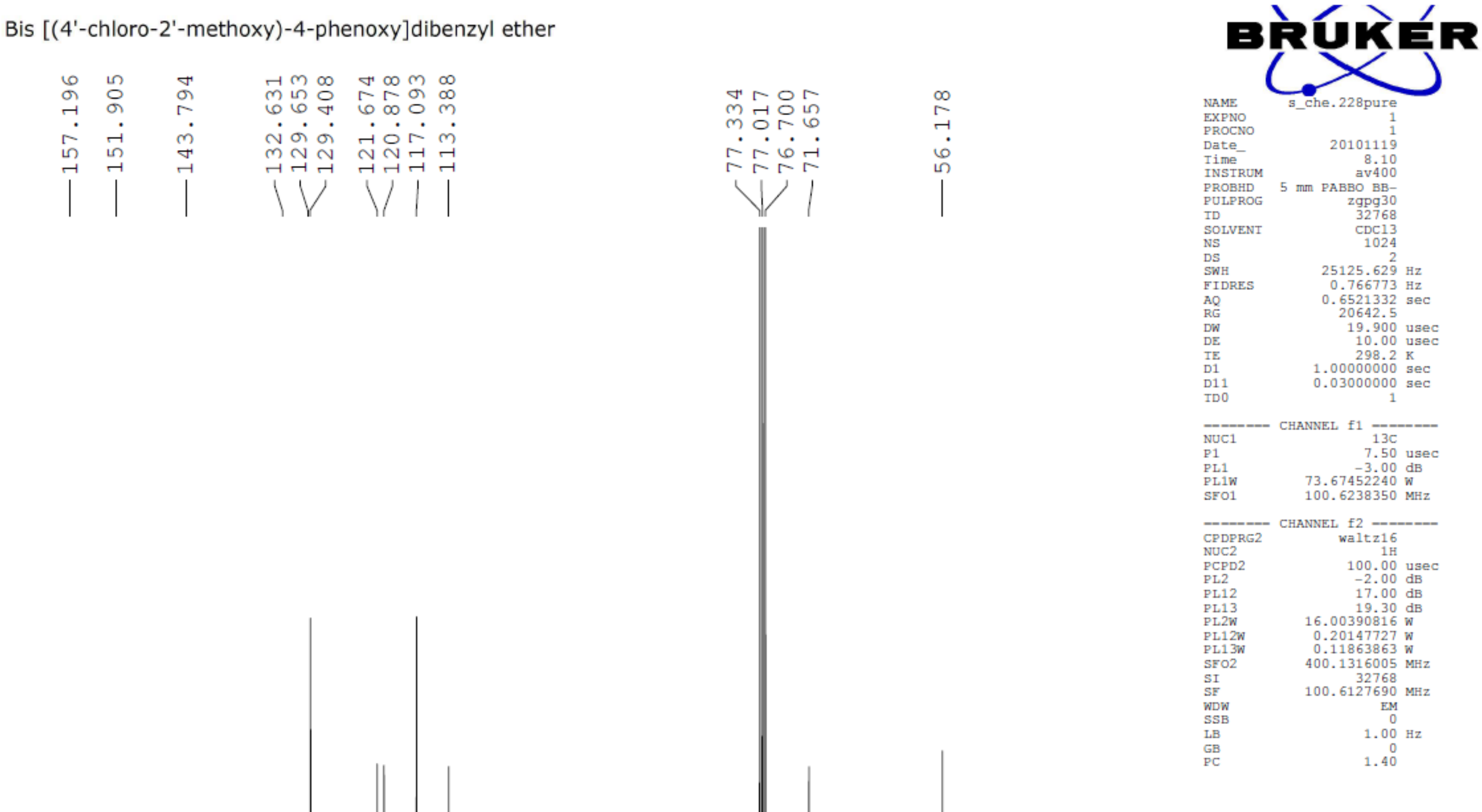

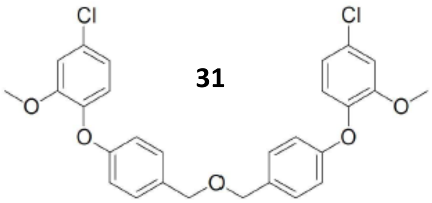

31

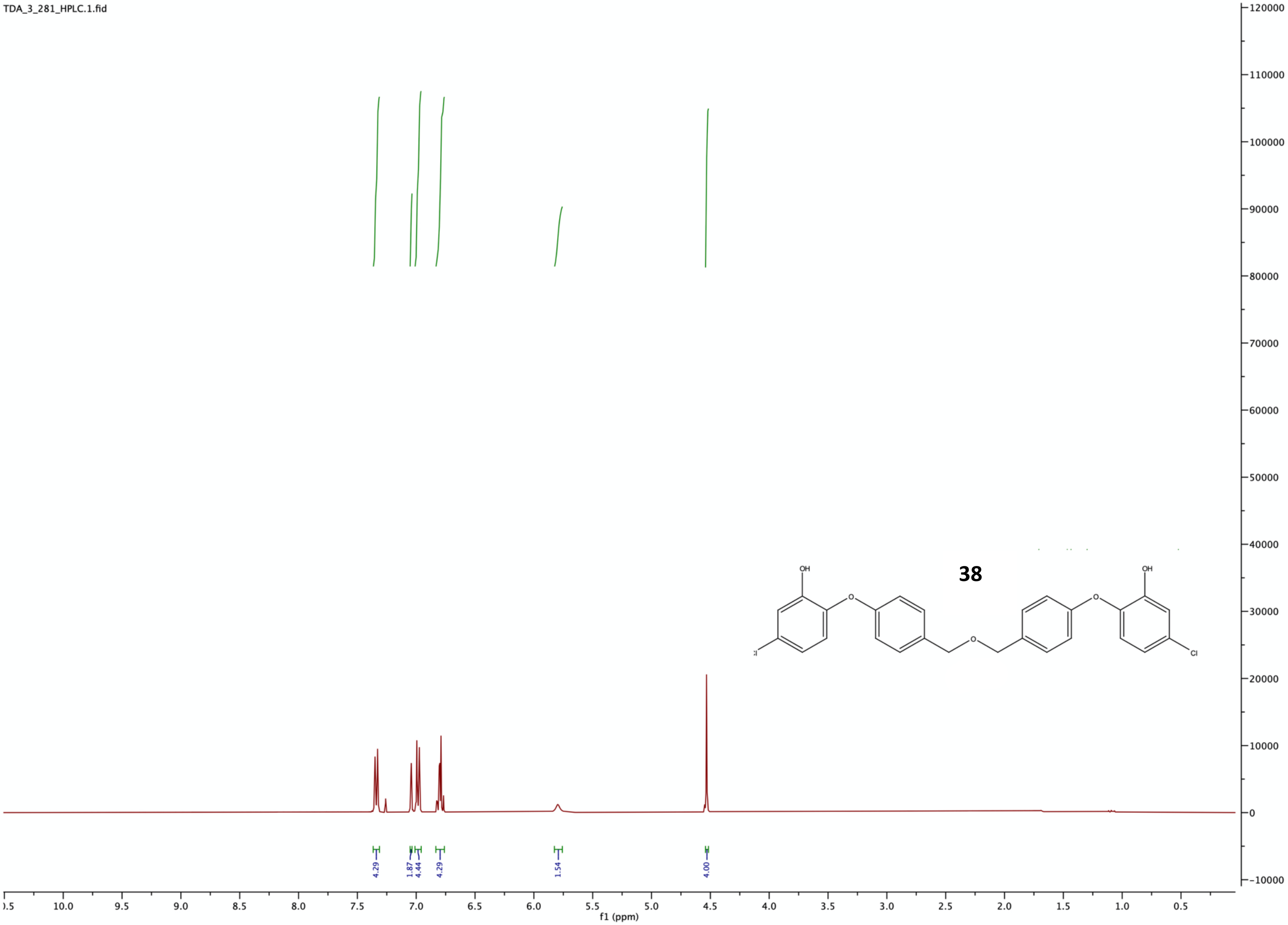
TDA_3_281_HPLC.1.fid
38
OH
O
O
O
OH
Cl
Cl
4.29
1.87
4.44
4.29
1.54
4.00
f1 (ppm)
10.0 9.5 9.0 8.5 8.0 7.5 7.0 6.5 6.0 5.5 5.0 4.5 4.0 3.5 3.0 2.5 2.0 1.5 1.0 0.5
120000 110000 100000 90000 80000 70000 60000 50000 40000 30000 20000 10000 0 -10000

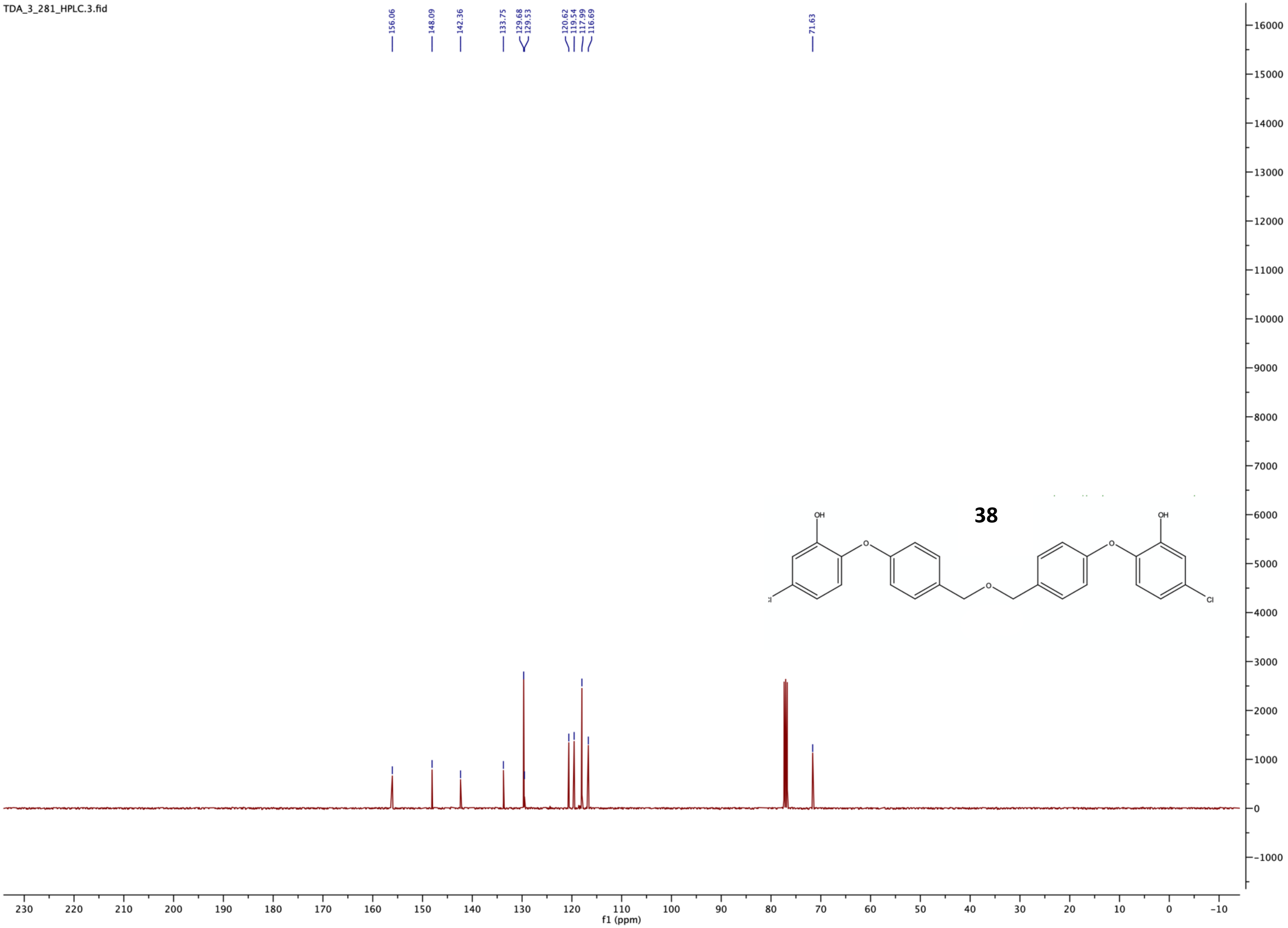
TDA_3_281_HPLC.3.fid
156.06
148.09
142.36
133.75
129.68
129.53
120.62
119.54
117.99
116.69
71.63
38
OH
O
Cl
f1 (ppm)

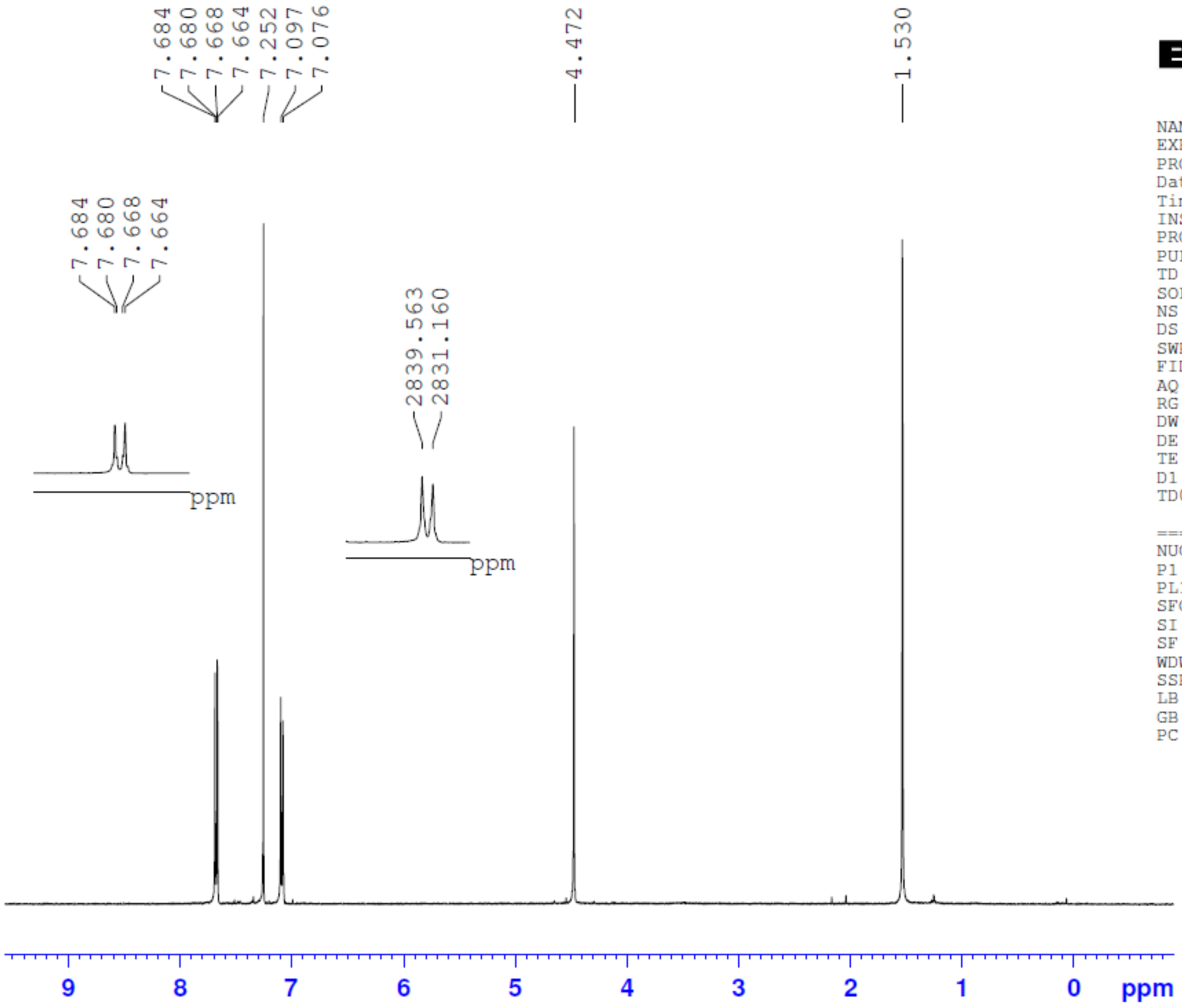

BRUKER

NAME	sc022
EXPNO	1
PROCNO	1
Date_	20080819
Time	13.16
INSTRUM	spect
PROBHD	5 mm QNP 1H/13
PULPROG	zg30
TD	65536
SOLVENT	CDCl3
NS	16
DS	2
SWH	8278.146 Hz
FIDRES	0.126314 Hz
AQ	3.9584243 sec
RG	1024
DW	60.400 usec
DE	6.50 usec
TE	298.2 K
D1	1.00000000 sec
TD0	1

======== CHANNEL f1 ========

NUC1	1H
P1	11.75 usec
PL1	0.00 dB
SFO1	400.1324710 MHz
SI	32768
SF	400.1300124 MHz
WDW	EM
SSB	0
LB	0.30 Hz
GB	0
PC	1.00

40

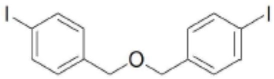

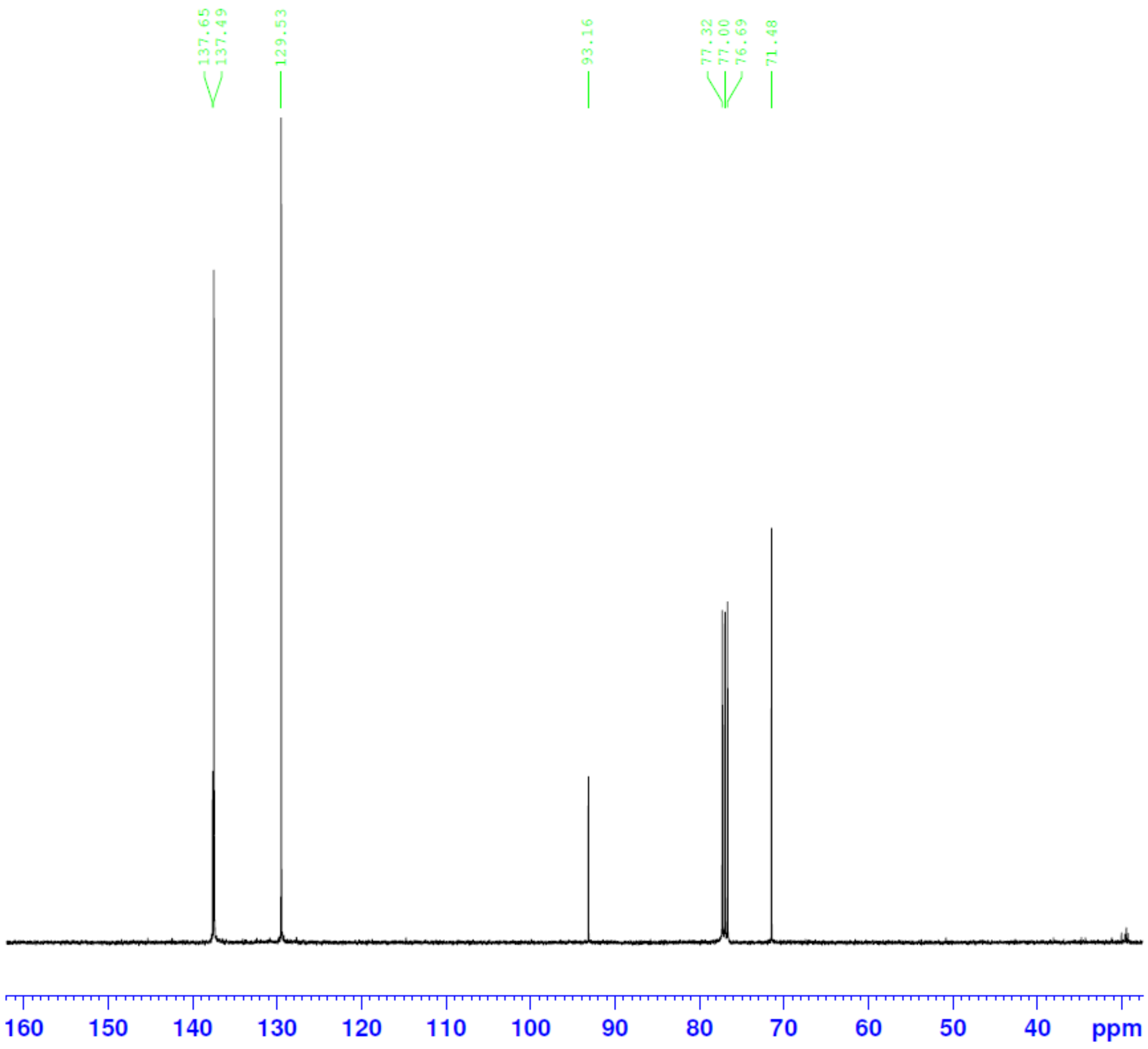

BRUKER

NAME	sc016b	
EXPNO	1	
PROCNO	1	
Date_	20080701	
Time	20.03	
INSTRUM	spect	
PROBHD	5 mm QNP 1H/13	
PULPROG	zgpg30	
TD	65536	
SOLVENT	CDCl3	
NS	1024	
DS	4	
SWH	23980.814	Hz
FIDRES	0.365918	Hz
AQ	1.3664756	sec
RG	1024	
DW	20.850	usec
DE	6.50	usec
TE	298.2	K
D1	2.00000000	sec
D11	0.03000000	sec
TD0	1	
======== CHANNEL f1 ========		
NUC1	13C	
P1	8.12	usec
PL1	0.00	dB
SFO1	100.6228298	MHz
======== CHANNEL f2 ========		
CPDPRG2	waltz16	
NUC2	1H	
PCPD2	80.00	usec
PL2	0.00	dB
PL12	18.00	dB
PL13	21.00	dB
SFO2	400.1316005	MHz
SI	32768	
SF	100.6127757	MHz
WDW	EM	
SSB	0	
LB	1.00	Hz
GB	0	
PC	1.40	

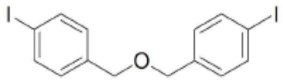

40

4-chloro-2-methoxy-1-(4-{[4-methoxybenzyl)oxy]methyl}phenoxy)benzene

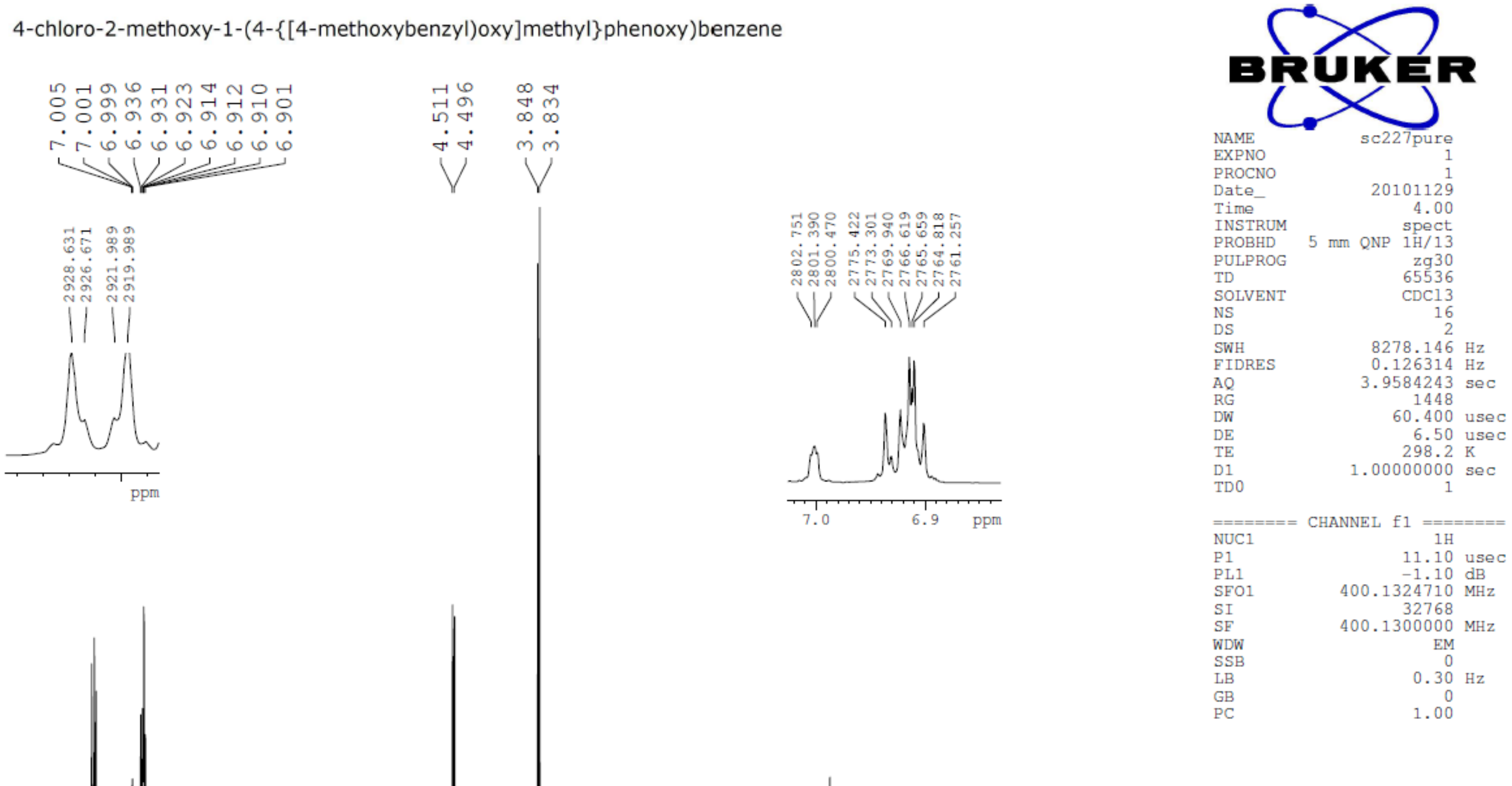

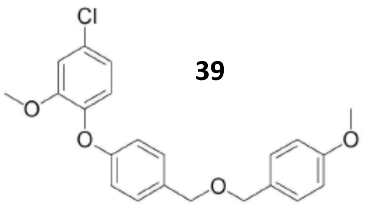

39

4-chloro-2-methoxy-1-(4-{[4-methoxybenzyl)oxy]methyl}phenoxy)benzene

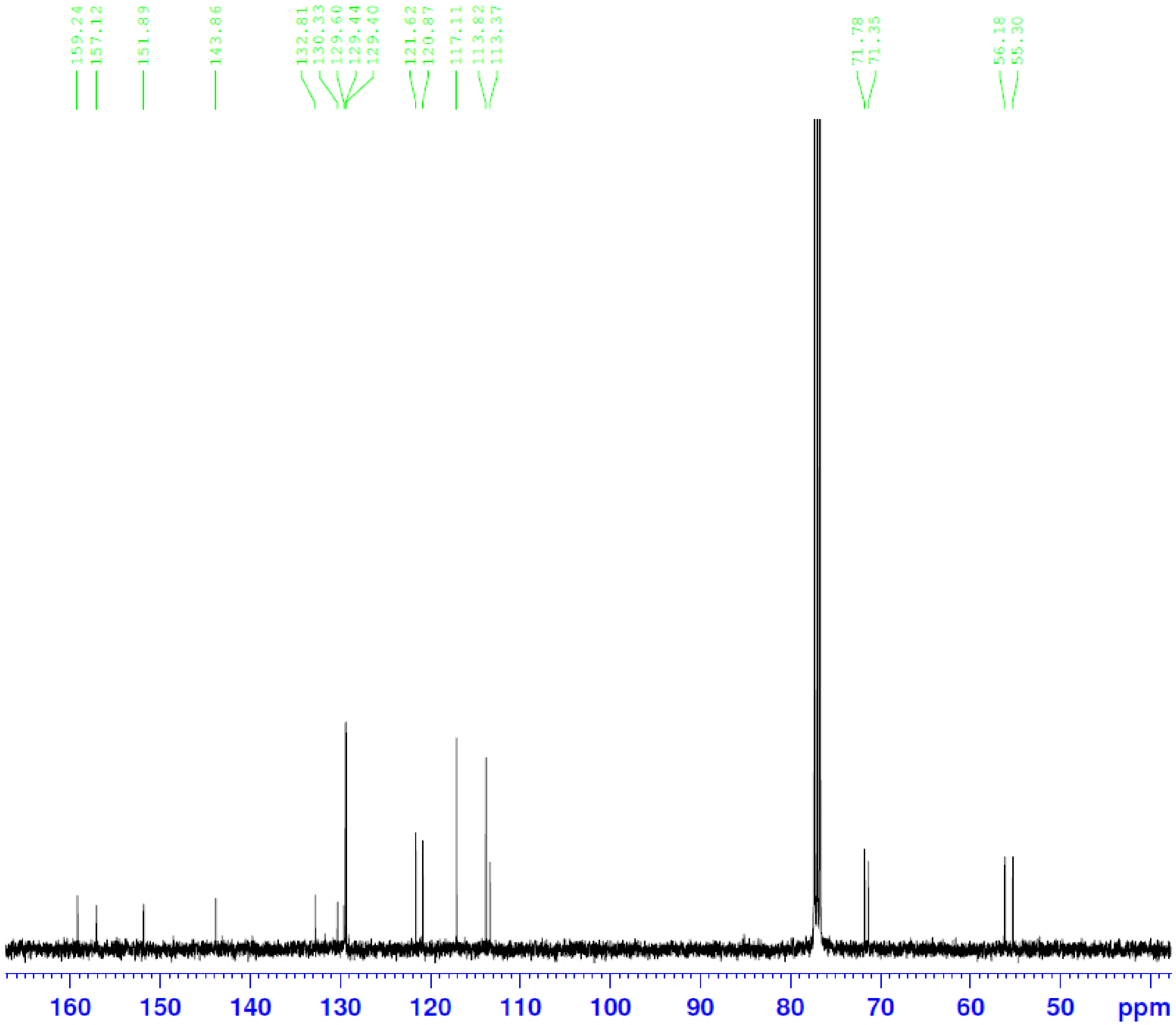

NAME	sc227pure_2	
EXPNO	2	
PROCNO	1	
Date_	20101129	
Time	5.00	
INSTRUM	spect	
PROBHD	5 mm QNP 1H/13	
PULPROG	zgpg30	
TD	65536	
SOLVENT	CDCl3	
NS	1024	
DS	4	
SWH	23980.814	Hz
FIDRES	0.365918	Hz
AQ	1.3664756	sec
RG	5792	
DW	20.850	usec
DE	6.50	usec
TE	298.2	K
D1	2.00000000	sec
D11	0.03000000	sec
TD0	1	
======== CHANNEL f1 ========		
NUC1	13C	
P1	9.38	usec
PL1	0.00	dB
SFO1	100.6228298	MHz
======== CHANNEL f2 ========		
CPDPRG2	waltz16	
NUC2	1H	
PCPD2	80.00	usec
PL2	-1.10	dB
PL12	16.06	dB
PL13	21.00	dB
SFO2	400.1316005	MHz
SI	32768	
SF	100.6127690	MHz
WDW	EM	
SSB	0	
LB	1.00	Hz
GB	0	
PC	1.40	

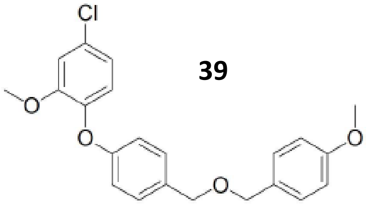

39

trans-2-octenoyl CoA

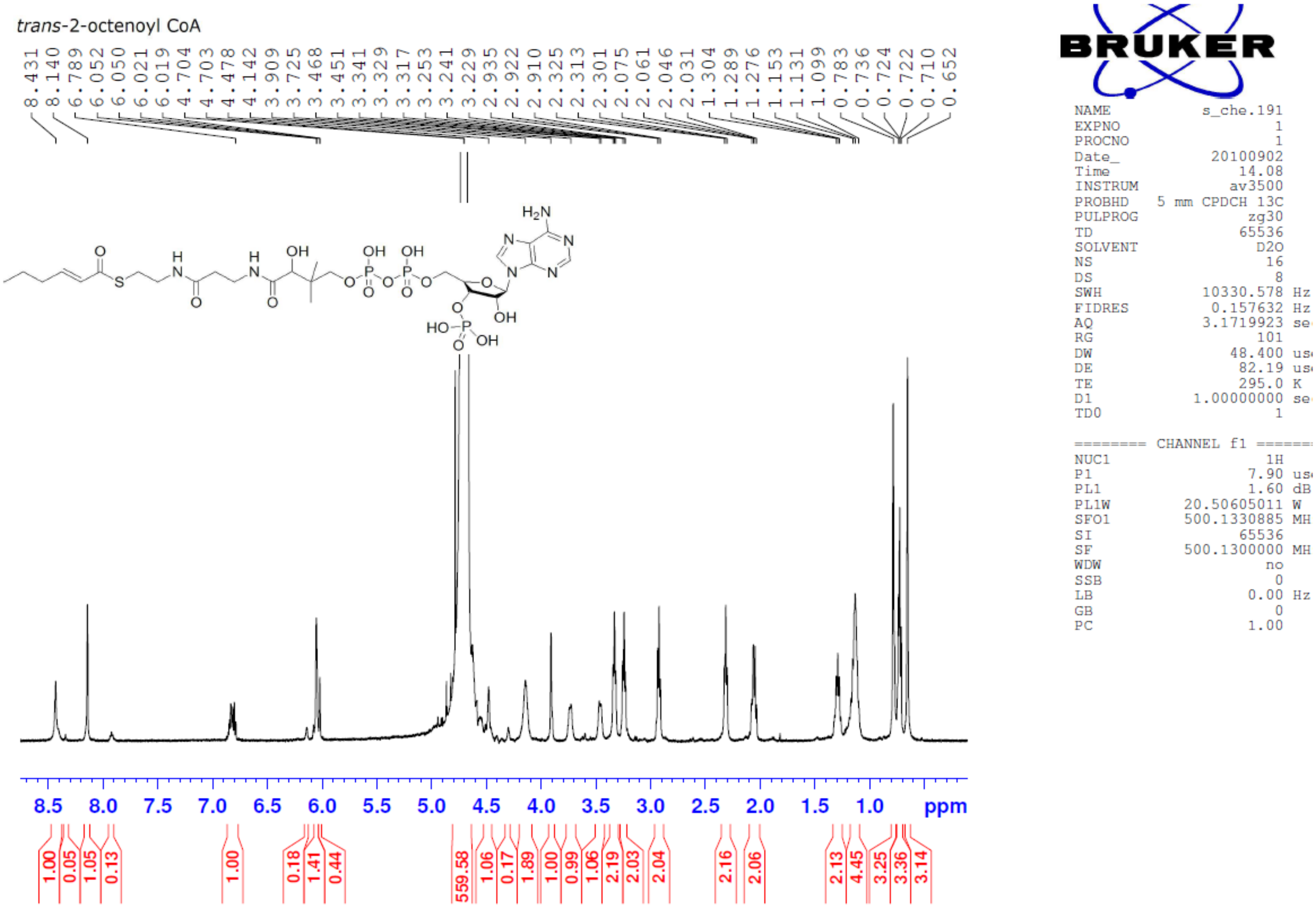

trans-2-octenoyl CoA

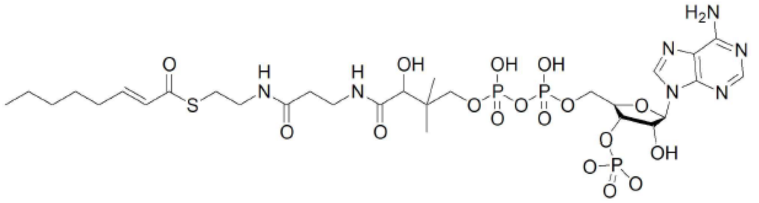

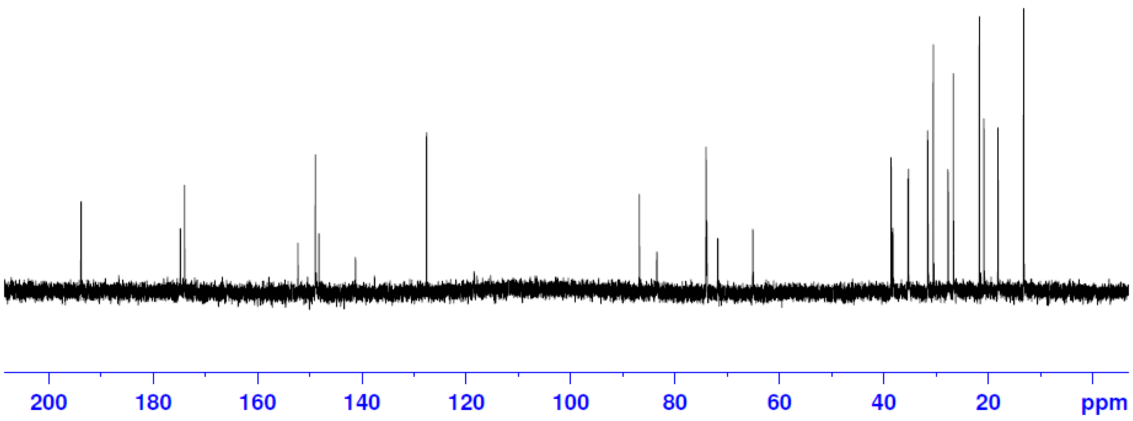

NAME	s_che.sc270
EXPNO	2
PROCNO	1
Date_	20110316
Time	13.53
INSTRUM	av3500
PROBHD	5 mm CPDCH 13C
PULPROG	zgpg30
TD	65536
SOLVENT	D2O
NS	515
DS	16
SWH	29761.904 Hz
FIDRES	0.454131 Hz
AQ	1.1010548 sec
RG	512
DW	16.800 use
DE	31.09 use
TE	298.0 K
D1	1.50000000 sec
D11	0.03000000 sec
TD0	1
======== CHANNEL f1 =======	
NUC1	13C
P1	7.30 use
PL1	1.40 dB
PL1W	66.60105133 W
SFO1	125.7716224 MHz
======== CHANNEL f2 =======	
CPDPRG2	waltz16
NUC2	1H
PCPD2	82.00 use
PL2	1.60 dB
PL12	21.39 dB
PL13	22.60 dB
PL2W	20.50605011 W
PL12W	0.21521972 W
PL13W	0.16288532 W
SFO2	500.1320005 MHz
SI	65536
SF	125.7577890 MHz
WDW	EM
SSB	0
LB	1.00 Hz
GB	0
PC	1.40

References

1. Thomas NR, Yang, Y, Drew, W.C, inventor; Lees, K.J, assignee. Biomolecular Labelling using multifunctional biotin analogues. United Kingdom patent WO 2010/106347 A2 2010.

2. Isaad AL, Barbetti F, Rovero P, D'Ursi AM, Chelli M, Chorev M, et al. N-alpha-Fmoc-Protected omega-Azido- and omega-Alkynyl-L-amino Acids as Building Blocks for the Synthesis of "Clickable" Peptides. *Eur J Org Chem*. 2008, 5308-14.

3. Vyas GN, Shah.N.M. Quinacetophene monomethy ether. *Org. Synth*. 1963, IV, 836-9.

4. Mackenzie.A.R. MCJ. Synthesis of bacterial coenzyme methoxatin. *Tetrahedron*. 1986, 42, 3259-68.

5. Van Benthem RATM, Hiemstra H, Speckamp WN. Synthesis of *N*-Boc-protected 1-amino-3-alken-2-ols from allylic carbamates via palladium(II)-catalyzed oxidative cyclization. *J Org Chem*. 1992, 57, 6083-5.

6. Connell RD, Rein T, Aakermark B, Helquist P. An efficient, palladium-catalyzed route to protected allylic amines. *J Org Chem*. 1988, 53, 3845-9.

7. Balestra MB, H.; Chen. D; Egle, I.; Forest, J.; Frey, J.; Isaac, M.; Ma, F.; David, N.; Slassi, A.; Steeleman, G.; Sun, G.; Sundar, B.; Ukkiramapandian, R.; Urbanek, R.A.; Walsh, S.; , inventor; Astrazeneca AB, NPS Pharma Inc, assignee. Pyrazolone compounds as metabotropic glutamate receptor agonists for the treatment of neurological and psychiatric disorders patent WO 2006071730. 2006.

8. Ouellet SG, Bernardi A, Angelaud R, O'Shea PD. Regioselective S(N)Ar reactions of substituted difluorobenzene derivatives: practical synthesis of fluoroaryl ethers and substituted resorcinols. *Tetrahedron Lett*. 2009, 50, 3776-9.

9. Drewe WC, Neidle S. Click chemistry assembly of G-quadruplex ligands incorporating a diarylurea scaffold and triazole linkers. *ChemComm*. 2008, 5295-7.

10. Adachi KA, Y.; Hanano, T.; Morimoto, H.; Hisadome, M, inventor; Mitsubishi Pharma Corp., assignee. Piperazine compounds and medicinal use thereof patent US 6455528 (B1). 2002.

11. Lee JWL, B.Y.; Kim, C.S, Lee, S.K.; Song, K.S.; Lee, S.J.; Shim, W.J.; Hwang, M.S., inventor; Yuhon Corp, assignee. Pyrimidine derivatives and processes for the preparation thereof patent US 6352993. 2002.

12. Pal M, Parasuraman K, Yeleswarapu KR. Palladium-Catalyzed Cleavage of O/N-Propargyl Protecting Groups in Aqueous Media under a Copper-Free Condition. *Org Lett*. 2003, 5, 349-52.

13. Spletstoser JT, Flaherty PT, Himes RH, Georg GI. Synthesis and Anti-Tubulin Activity of a 3-(4-Azidophenyl)-3-dephenylpaclitaxel Photoaffinity Probe. *J Med Chem*. 2004, 47, 6459-65.

14. Zhu W, Ma DW. Synthesis of aryl azides and vinyl azides via proline-promoted CuI-catalyzed coupling reactions. *ChemComm*. 2004, 888-9.

15. Schaetzer JN, K.; Stoller, A.; Hall, R.G.; Wenger, J.; Bondy, S.S.; Comer, D.D.; Penzotti, J.E.; Grootenhuis, P.D.J., inventor; Syngenta Participations, assignee. Novel Herbicides patent WO 2004002981. 2004.

16. Gibson SE, Mainolfi N, Kalindjian SB, Wright PT, White ALP. A new class of non-racemic chiral macrocycles: A conformational and synthetic study. *Chem Eur J.* 2005, 11, 69-80.

17. Murer PK, Lapierre JM, Greiveldinger G, Seebach D. Synthesis and properties of first and second generation chiral dendrimers with triply branched units: A spectacular case of diastereoselectivity. *Helv Chim Acta*. 1997, 80, 1648-81.

18. Marcoux J-FD, Sven; Buchwald, Stephen L. A General Copper-Catalyzed Synthesis of Diaryl Ethers. *J Am Chem Soc.* 1997, 119, 10539–40.

19. Manoury PM, Binet JL, Rousseau J, Lefevreborg FM, Cavero IG. Synthesis of a Series of Compounds Related to Betaxolol, a New Beta-1-Adrenoceptor Antagonist with a Pharmacological and

Pharmacokinetic Profile Optimized for the Treatment of Chronic Cardiovascular-Diseases. *J Med Chem.* 1987, 30, 1003-11.
20. Nahm MR, Potnick JR, White PS, Johnson JS. Metallophosphite-catalyzed asymmetric acylation of alpha,beta-unsaturated amides. *J Am Chem Soc.* 2006, 128, 2751-6.
21. Kopp F, Linne U, Oberthur M, Marahiel MA. Harnessing the chemical activation inherent to carrier protein-bound thioesters for the characterization of lipopeptide fatty acid tailoring enzymes. *J Am Chem Soc*. 2008, 130, 2656-66.
